# *Ortho* C–H arylation of arenes at room temperature using visible light ruthenium C–H activation†

**DOI:** 10.1039/d0sc01289k

**Published:** 2020-04-07

**Authors:** Arunachalam Sagadevan, Anastasios Charitou, Fen Wang, Maria Ivanova, Martin Vuagnat, Michael F. Greaney

**Affiliations:** School of Chemistry, The University of Manchester Oxford Road Manchester M13 9PL UK michael.greaney@manchester.ac.uk

## Abstract

A ruthenium-catalyzed *ortho* C–H arylation process is described using visible light. Using the readily available catalyst [RuCl_2_(*p*-cymene)]_2_, visible light irradiation was found to enable arylation of 2-aryl-pyridines at room temperature for a range of aryl bromides and iodides.

## Abstract 2

A ruthenium-catalyzed *ortho* C–H arylation process is described using visible light.

## Graphical abstract

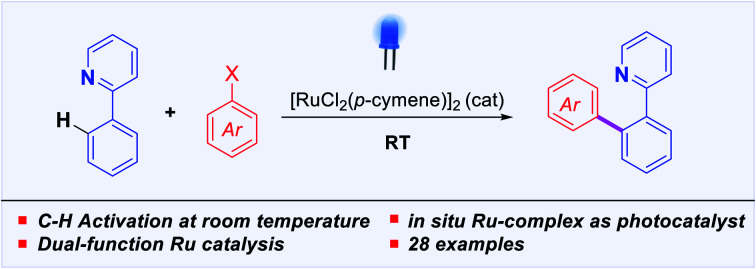

## Introduction

**Scheme 1:**
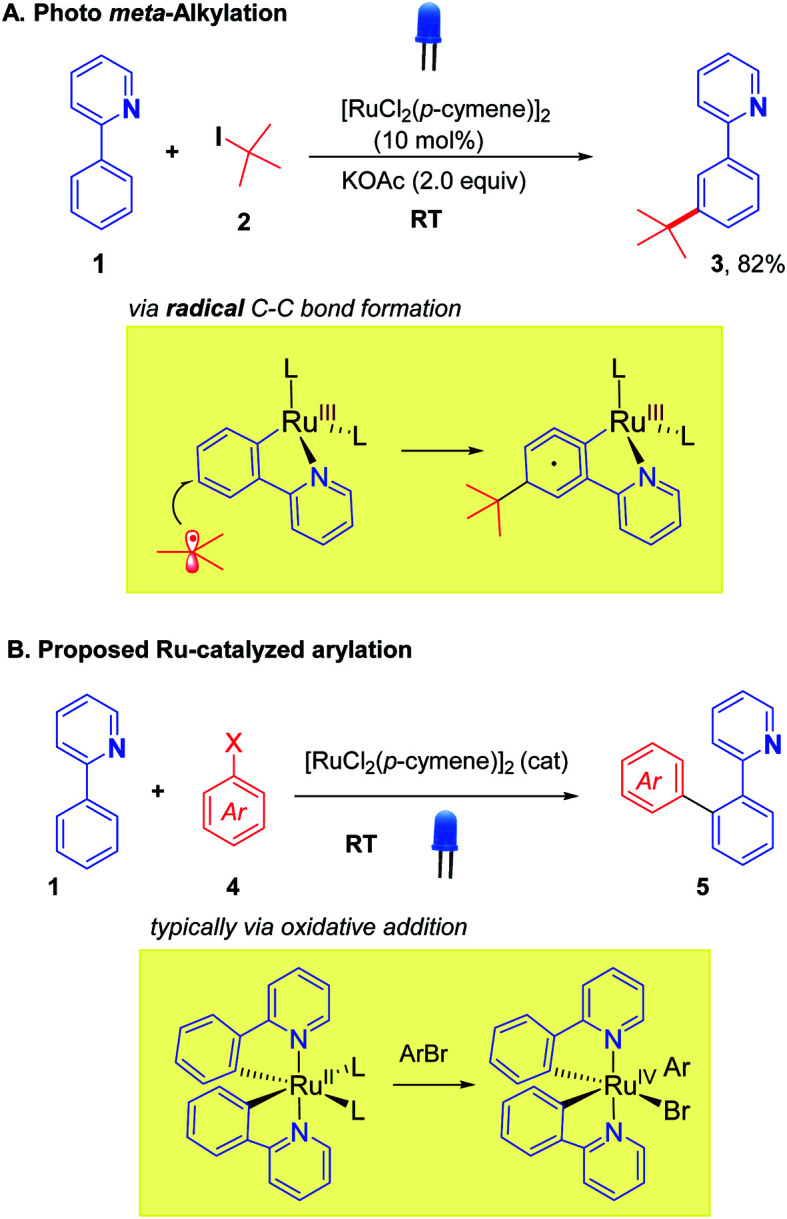
Ruthenium photocatalysis for *meta*-alkylation (A) and proposed *ortho*-arylation (B).

The integration of visible light photoredox catalysis with transition metal C–H activation catalysis creates new pathways for bond formation, that frequently operate under mild conditions. The two catalysis regimes can be meshed *via* two separate catalyst entities, with two corresponding catalysis cycles;^1^ or alternatively, a single dual-function catalyst system can be used.^2^ We are interested in this latter approach to exploit the facility of some Ru catalysts to absorb visible light, such that their native C–H activation function is enhanced in terms of improved rates, substrate scope, and environmental impact. We, along with the Ackermann group, recently demonstrated this concept for the Ru-catalysed *meta*-alkylation reaction (Scheme 1).^3^ The *tert*-butylation of 2-phenylpyridine **1**, typically carried out under thermal conditions (*ca.* 100 °C), could proceed at room temperature under blue-light irradiation using the widely employed catalyst [RuCl_2_(*p*-cymene)]_2_ to give the alkylated product **3** in good yield.

We were interested in exploring this concept of photo-Ru C–H activation in the arylation regime. Ru-catalysed *ortho*-arylation is a powerful approach to C–C bond formation that has seen extensive development in recent years.^4^ Proceeding *via* Ru(ii)/Ru(iv) catalytic cycles, the process has excellent scope for aryl halides (including aryl chlorides), is very tolerant of water and air, and the cost of Ru compares favorably with the far more expensive alternatives of Pd and Rh that are frequently used for *ortho* C–H arylation. High reaction temperatures are standard, however, when using [RuCl_2_(*p*-cymene)]_2_ as catalyst.^5,6^ A photo Ru arylation reaction could activate alternative mechanistic paths and offer the possibility of room temperature reaction.^7^ The two Ru-catalyzed processes, *meta*-alkylation and *ortho*-arylation, operate through very different frameworks in the thermal regime, with the former thought to involve discrete 2° and 3° carbon-centered radicals adding to Ru(iii) metallacycles (Scheme 1A),^8^ and the latter involving more typical oxidative additions of aryl halides to a Ru(ii) center.^9^ The role of photoexcitation on a putative arylation process was thus interesting to examine in light of this dichotomy; as the photoreductive formation of highly reactive aryl radicals in analogy to Ru-*meta* alkylation chemistry was unlikely to be a significant factor.

## Results and discussion

### Photoarylation of 2-arylazines with aryl halides

We began by examining the archetypal arylation system of 2-phenylpyridine **1a** with bromoanisole **4a** (Table 1). Using [RuCl_2_(*p*-cymene)]_2_ as catalyst in the presence of KOAc, under blue LED irradiation, we were pleased to observe successful arylation at room temperature to give a good 70% combined conversion to mono and di-*ortho*-arylated products **5a** and **5aa**. As is typical for Ru *ortho*-arylations of 2-phenyl pyridine, the monoarylated product is a superior substrate than the starting material, giving the diarylated material as the major product.

**Table 1:** Development of Ru photoarylation^a^

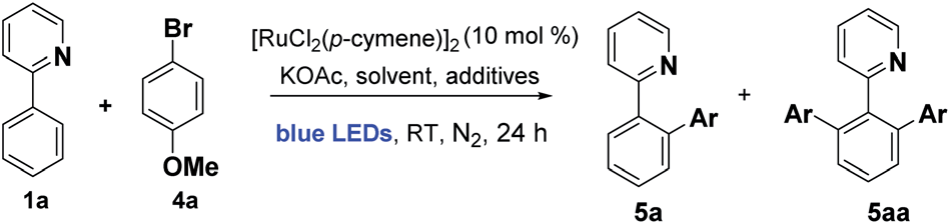				
Entry	Catalyst	Additives	Solvent	Yield^b^ [%]
1	[RuCl_2_(*p*-cymene)]_2_	MesCO_2_H	1,4-Dioxane	(70)
2	[RuCl_2_(*p*-cymene)]_2_	MesCO_2_H	DME	60
3	[RuCl_2_(*p*-cymene)]_2_	MesCO_2_H	THF	65
4	[RuCl_2_(*p*-cymene)]_2_	MesCO_2_H	2-MeTHF	(72)
5	[RuCl_2_(*p*-cymene)]_2_	N-Ac-l-isoleu	2-MeTHF	(74)
6	[RuCl_2_(*p*-cymene)]_2_	N-Ac-l-isoleu	2-MeTHF	(78)
7	[RuCl_2_(*p*-cymene)]_2_	N-Ac-l-isoleu/H_2_O	2-MeTHF	(80)
**8**	**[RuCl**_**2**_ **(*p*-cymene)]**_**2**_	**None/H**_**2**_ **O**	**2-MeTHF**	**(80)**
9	[RuCl_2_(*p*-cymene)]_2_	N-Ac-l-isoleu	MeOH, MeCN, DCM, or DMF	Trace
10	RuCl_3_·H_2_O	None	2-MeTHF	0
11	Ru(PPh_3_)_4_Cl_2_	N-Ac-l-isoleu/H_2_O	2-MeTHF	0
12	Ru(bpy)_3_Cl_2_	None	2-MeTHF	0
13	[RuCl_2_(*p*-cymene)]_2_ (5 mol%)	N-Ac-l-isoleu/H_2_O	2-MeTHF	(72)
14	No catalyst	N-Ac-l-isoleu	2-MeTHF	0
15	[RuCl_2_(*p*-cymene)]_2_ dark	N-Ac-l-isoleu/H_2_O	2-MeTHF	0
16	[RuCl_2_(*p*-cymene)]_2_ with air	N-Ac-l-isoleu/H_2_O	2-MeTHF	0

a Reaction conditions: **1a** (0.3 mmol), **4a** (0.6 mmol), [Ru] catalyst (10 mol%), additive (10 mol%), base 2.0 equiv., solvent (1 mL). The mixture was irradiated with blue LEDs (40–90 W power) for 24 h under N_2_ atmosphere.

b Yields in brackets refer to combined ^1^H NMR yield using 1,3,5-trimethylbenzene (mesitylene) as the internal standard, unbracketed yields are isolated. THF = tetrahydrofuran, 2-MeTHF = 2-methyl tetrahydrofuran, DME = 1,2-dimethoxyethane, DMF = dimethyl formamide, DCM = dichloromethane.

The reaction worked well in ethereal solvents (entries 1–4), and a screen of carboxylate and amino acid additives did not yield substantial improvements, although the acetylisoleucine derivative gave slightly improved yields and was retained for substrate screening (entry 7). Common solvents such as MeOH, DCM, MeCN, or DMF were not effective (entry 8), nor were the simple Ru salts shown in entries (10–12). Control experiments established that both light and catalyst were essential for reaction at room temp (entries 13–15), and as with our previous Ru *meta* systems,^10^ the reaction was found to be air sensitive and require inert atmospheres to proceed.

**Scheme 2:**
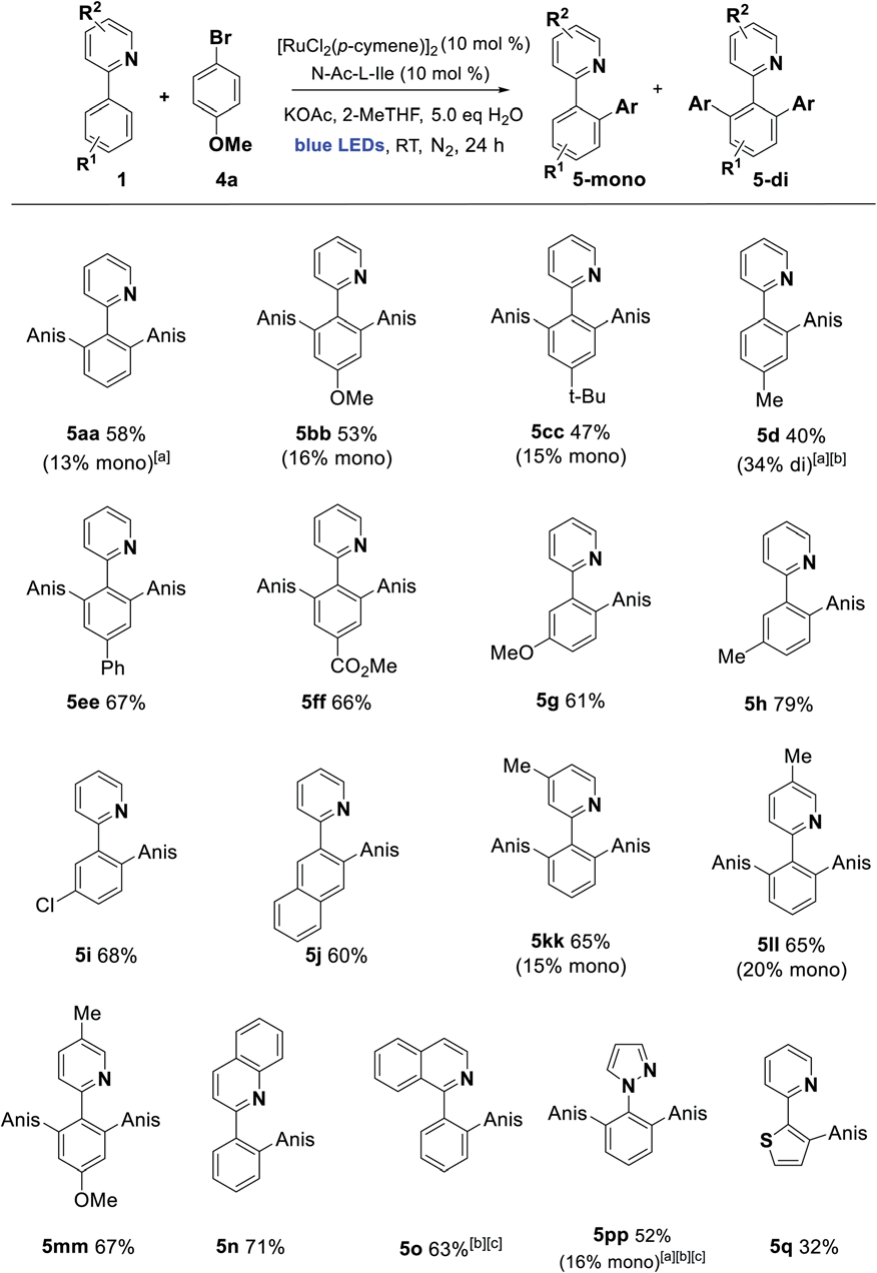
Photoarylation of 2-arylazines with *p*-bromoanisole (Anis = 4-BrC_6_H_4_). Unless otherwise noted, reaction conditions were as follows: **1** (1.0 eq., 0.3 mmol), **4a** (2.0 eq., 0.6 mmol), [Ru] catalyst (10 mol%), additive (10 mol%), base (2.0 eq.), solvent (1 mL), H_2_O (5 eq.). The mixture was irradiated with blue LEDs (40–90 W power) for 24 h under N_2_ (1 atm). The yields refer to isolated yields after purification by column chromatography on silica gel (major product illustrated). ^*a*^Reaction performed using 0.250 mmol of **1** (1.0 eq.) and 0.375 mmol of **4a** (1.5 eq.). ^*b*^Reaction performed without the addition of N-Ac-l-Ile. ^*c*^Reaction run for 40 h.

With these conditions in hand, we established the scope of the reaction with respect to the C–H component using *p*-bromoanisole as the arylating agent. Alkyl, alkoxy, carboxy, and phenyl substitution was tolerated in the 4-position of the arene, favoring the disubstituted product (**5aa–5ff**). Substitution in the 3-position with MeO, Me, Cl, and fused ring of the naphthyl group acted as a steric control element, suppressing the second *ortho* ruthenation step and yielding mono-arylated products exclusively (**5g–5j**). Some alterations to the directing group were possible, with alkylated pyridines being effective in the reaction, along with bicyclic quinoline and isoquinoline directing groups (**5kk–5o**) and the pyrazine **5pp**. Finally, changing the arene C–H to the 5-membered heteroarene thiophene C–H was partially successful in the photoarylation, affording the novel pyridyl thiophene **5q** but in diminished yield (Scheme 2).

**Scheme 3:**
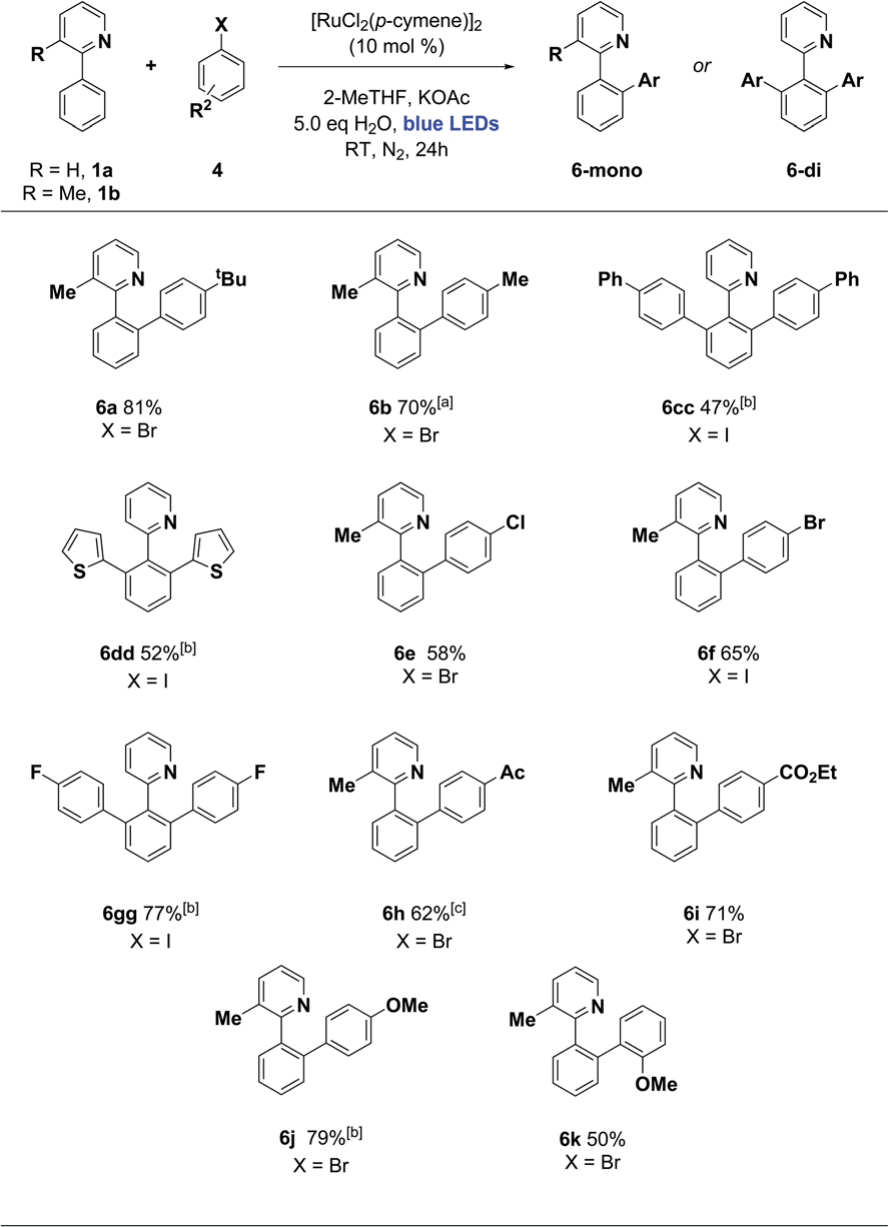
Photoarylation of 2-arylpyridines with various aryl halides. Unless otherwise noted, reaction conditions were as follows: **1** (1.0 eq., 0.25–0.30 mmol), **4** (1.5 eq., 0.375–0.45 mmol), [Ru] catalyst (10 mol%), base (2.0 eq.), solvent (1 mL), H_2_O (5 eq.). The mixture was irradiated with blue LEDs (40–90 W power) for 24 h in N_2_ (1 atm). The yields refer to isolated yields after purification by column chromatography on silica gel. ^*a*^Reaction run for 72 h. ^*b*^Reaction performed using 0.3 mmol of **1** (1.0 eq.) and 0.6 mmol of **4** (2.0 eq.) in the presence of N-Ac-l-Ile (10 mol%). ^*c*^Reaction run for 40 h.

We then turned our attention to the aryl halide coupling partner, and were pleased to find broad substrate scope across a variety of aryl bromides and iodides (Scheme 3). We used 3-methyl-2-phenylpyridine (**1b**) as the substrate in the majority of cases, to simplify the reaction pathway for monoarylation. We observed good efficiencies for 4-alkyl and moderate efficiencies for 4-phenyl aryl halides (**6a–6cc**), along with the 2-thienyliodide substrate (**6dd**). Halogens were well tolerated (**6e–6gg**), and electron poor (4-keto and 4-carboxy ester groups) along with electron rich (4-methoxy) aryl halides reacted smoothly in each case. *Ortho*-substituted aryl halides were not generally effective in this protocol, although 1-bromo-2-methoxybenzene did react successfully to give **6k** in 50% yield. This *ortho* substitution pattern is known to favour an oxidative dimerization pathway for aryl halide substrates.^11^

### Mechanistic studies

The exclusive formation of *ortho*-arylated products, allied with the high reduction potential of aryl halides (*ca.* −2.5 eV)^12^ would likely preclude any discrete aryl radical generation through SET from a Ru(ii) catalytic species in the reaction. This was supported with radical quenching experiments, where the reaction proceeded to reasonable conversion in the presence of both BHT and 1,1-diphenylethene (DHP), although TEMPO was observed to completely inhibit the reaction forming an insoluble black suspension. A competition experiment between the electron-rich bromoanisole **4a** and electron-poor bromo-benzoate afforded a 3 : 1 ratio of products in favour of the benzoate **6i**, in line with what is commonly observed in high-temperature Ru *ortho*-arylation through an oxidative addition mechanism.^13^

**Scheme 4:**
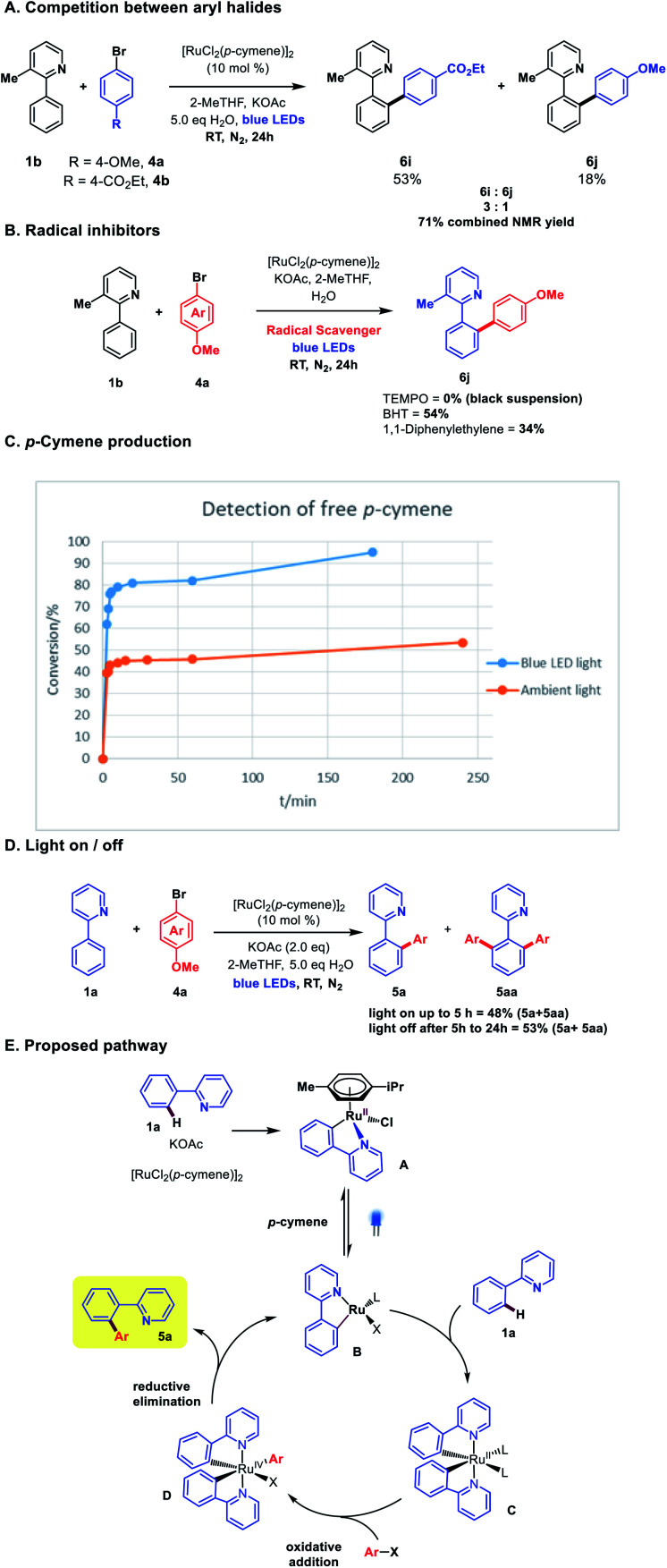
(A) Intermolecular competition experiment between aryl halides. (B) Reaction in the presence of radical inhibitors. (C) Detection of free *p*-cymene. Conversion refers to amount of *p*-cymene produced. (D) Light on/off experiment. (E) Proposed pathway. BHT = butylated hydoxytoluene.

Recent investigations from Larrosa and co-workers into the mechanism of Ru(ii)-*ortho* arylation have identified the inhibitory role of the cymene ligand in [RuCl_2_(*p*-cymene)]_2_ catalysis.^6,14^ While this ligand affords an air-stable, easy to use catalyst, it retards activity in catalytic cycles, and must de-complex to enable the formation of the active bis-cycloruthenated complex **C** (Scheme 4E). We analysed the free cymene formed in the reaction under room temperature blue light irradiation against an ambient light control. Decomplexation was observed in both cases, but with a clear rate increase under blue LED irradiation (Scheme 4C). Photo-decomplexation of η^6^-arenes such as cymene is well known, and has been used as a triggering mechanism for both chemical and biological activity in Ru(ii) complexes.^15^ However, a light/dark experiment (Scheme 4D) demonstrated that continuous irradiation was necessary for complete conversion. This suggests that either reversible cymene re-complexation can inhibit the reaction, or there is an additional role for visible light in the catalytic cycle (*e.g.* photoexcitation of complexes **C** or **D** to facilitate either oxidative addition or reductive elimination, respectively). Stern–Volmer experiments with stoichiometric pre-catalyst **A** and aryl halides did not show clear evidence of photoluminescence quenching, but we cannot rule out an additional role for visible light in subsequent steps in the catalytic cycle at this time.

## Conclusions

We have established a room temperature, ruthenium catalysed *ortho*-arylation reaction that proceeds at room temperature under the agency of visible light irradiation. The reaction encompasses a wide selection of aryl halides, producing C–H arylated products that are typically accessed at temperatures in excess of 100 °C. Initial observations point to a photo-decomplexation of the cymene ligand from the ruthenium pre-catalyst as playing a key role in the catalytic cycle. Future work will extend the photoarylation to new C–H substrate classes, and further delineate the role of visible light in the mechanism.

## Conflicts of interest

There are no conflicts to declare.

## Supplementary Material

SC-011-D0SC01289K-s001

## References

[cit1] Levin M. D., Kim S., Toste F. D. (2016). ACS Cent. Sci..

[cit2] Ding W., Lu L.-Q., Zhou Q. Q., Wei Y., Chen J.-R., Xiao W.-J. (2017). J. Am. Chem. Soc..

[cit3] Sagadevan A., Greaney M. F. (2019). Angew. Chem., Int. Ed..

[cit4] Nareddy P., Jordan F., Szostak M. (2017). ACS Catal..

[cit5] Drev M., Grošelj U., Ledinek B., Perdih F., Svete J., Štefane B., Požgan F. (2018). Org. Lett..

[cit6] Simonetti M., Cannas D. M., Just-Baringo X., Vitorica-Yrezabal I. J., Larrosa I. (2018). Nat. Chem..

[cit7] Ghosh I., Marzo L., Das A., Shaikh R., König B. (2016). Acc. Chem. Res..

[cit8] Paterson A. J., John-Campbell S. S., Mahon M. F., Press N. J., Frost C. G. (2015). Chem. Commun..

[cit9] Shan C., Zhu L., Qu L.-B., Bai R., Lan Y. (2018). Chem. Soc. Rev..

[cit10] Teskey C. J., Lui A. Y. W., Greaney M. F. (2015). Angew. Chem., Int. Ed..

[cit11] Rogge T., Ackermann L. (2019). Angew. Chem., Int. Ed..

[cit12] Andrieux C. P., Blocman C., Dumas-Bouchiat J. M., Saveant J. M. (1979). J. Am. Chem. Soc..

[cit13] Ackermann L., Vicente R., Potukuchi H. K., Pirovano V. (2010). Org. Lett..

[cit14] Marce P., Paterson A. J., Mahon M. F., Frost C. G. (2016). Catal. Sci. Technol..

[cit15] Karslyan E. E., Perekalin D. S., Petrovskii P. V., Lyssenko K. A., Kudinov A. R. (2008). Russ. Chem. Bull..

