# Supplementary material for: Ortho C–H arylation of arenes at room temperature using visible light ruthenium C–H activation
Source: Chem Sci. 2020 Apr 7;11(17):4439–43. doi: 10.1039/d0sc01289k (PMC8159458; doi:10.1039/d0sc01289k)
Supplement: SC-011-D0SC01289K-s001 [file SC-011-D0SC01289K-s001.pdf]

Electronic Supplementary Material (ESI) for Chemical Science.
This journal is © The Royal Society of Chemistry 2020

ortho C-H Arylation of Arenes at Room Temperature Using Visible Light Ruthenium C-H Activation

Arunachalam Sagadevan, Anastasios Charitou, Fen Wang, Maria Ivanova, Martin Vuagnat and Michael F. Greaney*

School of Chemistry, The University of Manchester, Oxford Road, Manchester, M13 9PL, United Kingdom. E-mail: Michael.greaney@manchester.ac.uk

Supporting Information

Table of contents

Experimental section S2

General arylation procedure S3

Radical trap experiment S4

Light on-off experiment S4

Competition experiment S5

Detection of free *p*-cymene S6

Compound characterization data S8

^{1}H NMR and ^{13}C NMR spectra S19

Experimental section

General: All reactions were conducted under an atmosphere of nitrogen with oven-dried glassware. All photochemical reactions were conducted using a blue light-emitting diode (LED) as the visible-light source (90 W blue LED lamps (Kessil KSH150B Blue LED Grow Light; 5-7 cm away, with cooling fan to maintain the reaction at room temperature, 25-30 °C). Solvents were purchased in anhydrous quality and used as received. Starting materials were commercially available (Sigma-Aldrich or Alfa-Aesar or TCI-chemicals) and used as received or starting materials, with the exception of 2-phenylpyridines[s1] being synthesized according to literature procedures. For the scope, all the yields refer to isolated compounds. Column chromatography was conducted by manual column (silica gel) or using Biotage Snap Ultra cartridges on a Biotage Isolera automated columning machine. ^{1}H, ^{13}C NMR spectra were recorded at 298 K on either 400 or 500 MHz Bruker NMR spectrometers as stated. Signal positions were recorded in δ ppm and measured from the centre of the signal, excluding multiples, which are given as a range. Splitting patterns are reported using the abbreviations s, br. s, d, t, q, quin, sept and m (or combinations thereof) denoting singlet, broad singlet, doublet, triplet, quartet, quintet, septet and multiplet respectively. The use of the app abbreviation refers to apparent. All ^{1}H NMR and 13C chemical shifts were referenced to the residual solvent peak of $CDCl_3$ (^{1}H referenced to 7.26 ppm and ^{13}C referenced to 77.16 ppm). All coupling constants, J, are quoted in Hz and reported to the nearest 0.1 Hz as observed in the spectra. Thin layer chromatography (TLC) was performed on commercially available pre-coated TLC plates (Alugram® Sil G/UV254). The visualisation was achieved under UV light at 254 nm. All mass spectrometry was carried out by the mass spectrometry service at the University of Manchester. High-resolution mass spectrometry (HRMS) was completed on a Waters QTOF micro with ESI/APCI ionisation. Low-resolution mass spectrometry was performed on Waters SQD2 Q-MS, Agilent 5975C Triple Axis GCMS or Hewlett Packard 5971 MSD.

***General arylation procedure*:**

Scheme S1

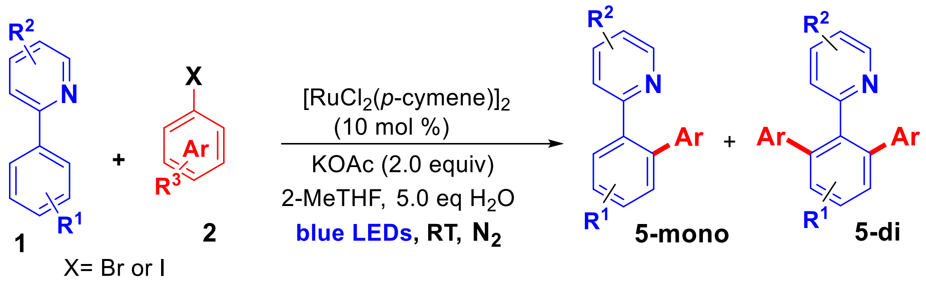

A 5 mL microwave vial was charged with a magnetic stirring bar, $[RuCl_2(p\text{-cymene})]_2$, (10 mol %), KOAc (2.0 eq), aryl halides **2** (1.5 eq), and phenylpyridines **1** (0.25 – 0.3 mmol, 1.0 eq) (*if phenylpyridine **1** is solid then added at this point before sealing the vial*). The vial was then sealed and evacuated under vacuum for 15-20 minutes and backfilled with nitrogen five times. 2-Methyl tetrahydrofuran (1 mL), H_2O (5.0 equiv) and finally 2-phenylpyridines **1** (0.25 – 0.3 mmol, 1.0 equiv) (*if phenylpyridine **1** is liquid then added last via syringe*) were added via syringe through the septum. The reaction mixture (in microwave vial) was sonicated 2-3 minutes at 25 °C and a yellow-orange suspension was formed. The reaction mixture was then irradiated with blue LEDs (90 W blue LED lamps (Kessil KSH150B Blue LED Grow Light; 5-7 cm away, with a cooling fans to maintain the reaction at room temperature, 25-30 °C) under N_2 (1 atm) until completion of the reaction (monitored by TLC). The mixture was filtered through celite and silica gel pads and washed with ethyl acetate. The filtrate was concentrated, and the residue was purified by column chromatography on silica gel to collect the expected *ortho* C-H arylation products.

Radical trap experiment

Scheme S2

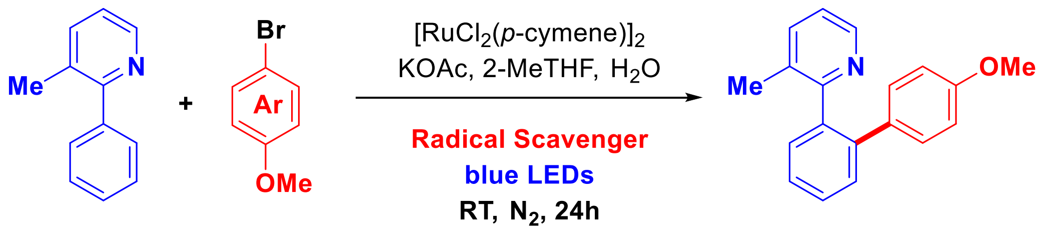

1b **2a** **6k**

TEMPO = **0% (black suspension)**
BHT = **54%**
1,1-Diphenylethylene = **34%**

A 25 mL microwave vial was charged with a magnetic stirring bar, $[RuCl_2(p\text{-cymene})]_2$, (0.025 mmol, 10 mol %, 15.3 mg), KOAc (2.0 eq, 0.51 mmol) and the radical scavenger (1.5 eq, 0.37 mmol). The vial was then sealed and evacuated under vaccum for 30 minutes and backfilled with nitrogen three times. 2-MeTHF (1 mL), H_2O (5.0 eq), *p*-bromoanisole **2a** (1.5 eq, 0.37 mmol) and 3-methyl-2-phenylpyridine (1.0 eq, 0.25 mmol) were added with a syringe through a septum. The reaction mixture was sonicated for 5 min at 25 °C and a yellow-orange suspension was formed. It was then irradiated with blue LEDs under N_2 atmosphere for 24 h. The solvent was evaporated and the residue was purified by column chromatography on silica gel.

Light on-off experiment: two separate 5 mL microwave vials were charged with a magnetic stirring bar, $[RuCl_2(p\text{-cymene})]_2$, (0.03 mmol, 10 mol %), and KOAc (2.0 eq, 0.6 mmol). The two vials were then sealed and evacuated under vaccum for 15-20 minutes and backfilled with nitrogen five times. 2-Methyltetrahydrofuran (1 mL), 4-bromoanisole **2a** (0.45 mmol, 1.5 eq), H_2O (5.0 eq) and finally 2-phenylpyridine **1a** (0.3 mmol, 1.0 eq) were added via syringe using the septum. The reaction mixtures (in microwave vials) were sonicated 2-3 minutes at 25 °C and and a yellow-orange suspension was formed. The reaction mixtures were then irradiated with blue LEDs (90 W blue LED lamps (Kessil KSH150B Blue LED Grow Light; 5-7 cm away, with the cooling fan to maintain the reaction at room temperature, 25-30 °C) under N_2 (1 atm).

After irradiation for 5 h, the lamp was switched off and the first reaction mixture was worked up and purified to give the *ortho* C-H arylation product **5a** & **5aa** in 48% total yield (column chromatography). The second vial was left stirring for a further 19 hours in the dark (30 °C), giving 24-hour total reaction time. Work-up and purification gave ortho products **5a** & **5aa** in total yield of 53% (see Scheme S3). *The*

light on-off-experiment suggests that the observed photochemical ortho-C-H action of arenes is not a chain reaction, and requires constant irradiation for complete reaction.

Scheme S3

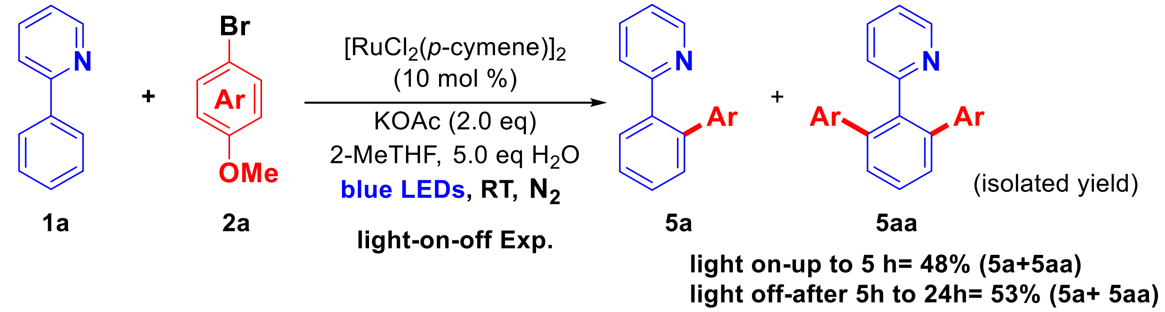

Competition experiment

Scheme S4

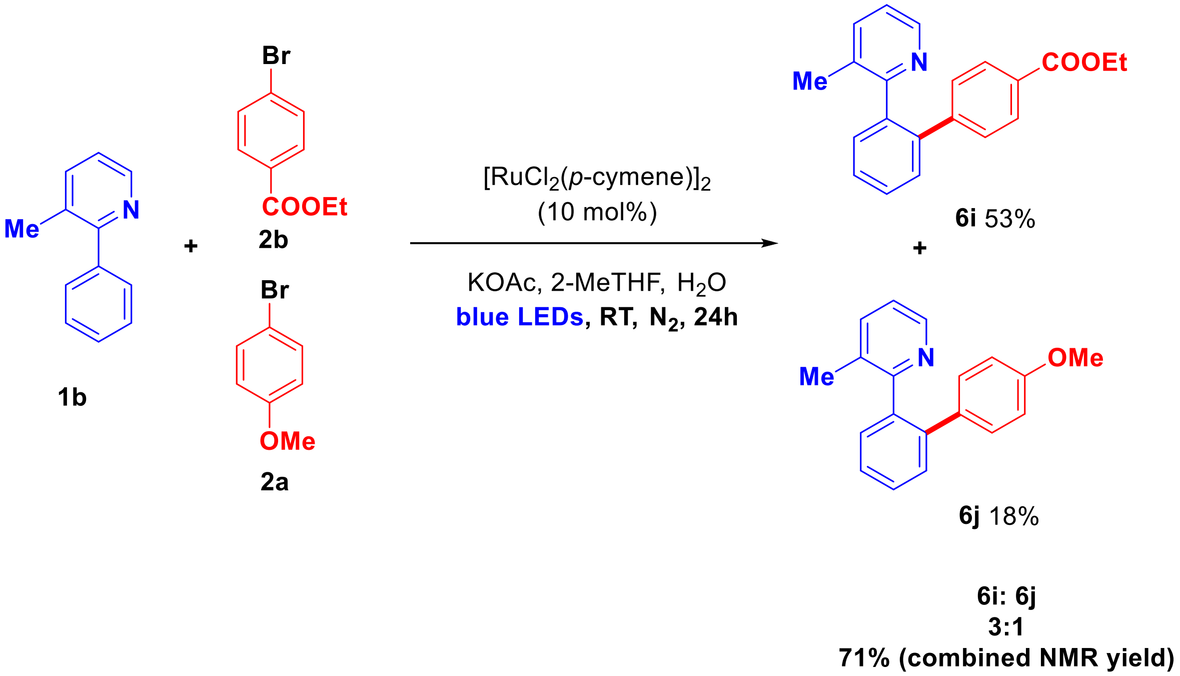

A 5 mL microwave vial was charged with a magnetic stirring bar, $[RuCl_2(p\text{-cymene})]_2$, (0.025 mmol, 10 mol %) and KOAc (2.0 eq, 0.50 mmol). The vial was then sealed and evacuated under vacuum for 15-20 minutes and backfilled with nitrogen five times. 2-Methyltetrahydrofuran (1 mL), 4-bromoanisole **2a** (0.50 mmol, 2.0 eq), ethyl 4-bromobenzoate **2b** (0.50 mmol, 2.0 eq), H_2O (5.0 eq) and finally 3-methyl-2-phenylpyridine (0.25 mmol, 1.0 eq) were added via syringe using the septum. The reaction mixture (in microwave vial) was sonicated 2-3 minutes at 25 °C and a yellow-orange suspension was formed. The

reaction mixture was then irradiated with blue LEDs (90 W blue LED lamps (Kessil KSH150B Blue LED Grow Light; 5-7 cm away, with the cooling fan to maintain the reaction at room temperature, 25-30 °C) under N_2 (1 atm) for 24 h. The solvent was evaporated and the residue was purified by column chromatography on silica gel to afford a 3:1 mixture of **6i** and **6j**. The ratio of the products was determined by ^{1}H NMR using 1,3,5-trimethylbenzene (mesitylene) as internal standard.

Detection of free *p*-cymene

Scheme S5

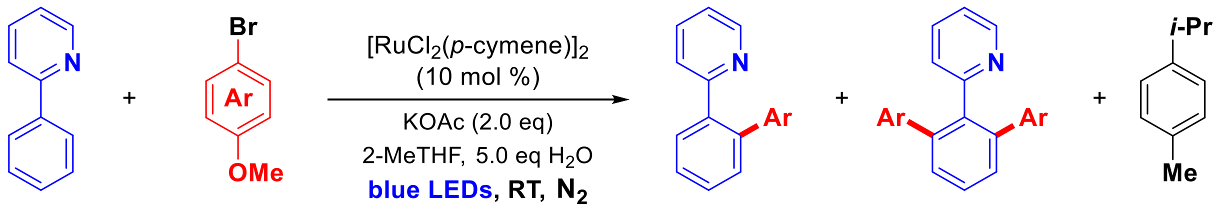

Under an atmosphere of N_2, a Schlenk-tube was charged with 2-phenylpyridine **1a** (232.8 mg, 1.50 mmol), 4-bromoanisole **2a** (420.8 mg, 2.25 mmol), $[RuCl_2(p\text{-cymene})]_2$ (92.0 mg, 150 µmol, 10.0 mol %), KOAc (294.0 mg, 3.00 mmol) and water (135 µL). 2-MeTHF (5.0 mL) was added and the reaction mixture was sonicated 2-3 minutes at 25 °C and a yellow-orange suspension was formed. The reaction mixture was then irradiated with blue LEDs. During the course of the reaction aliquots (100 µL) were removed via a syringe after, diluted with DCM, filtered through a short plug of silica gel and analyzed by gas chromatography.

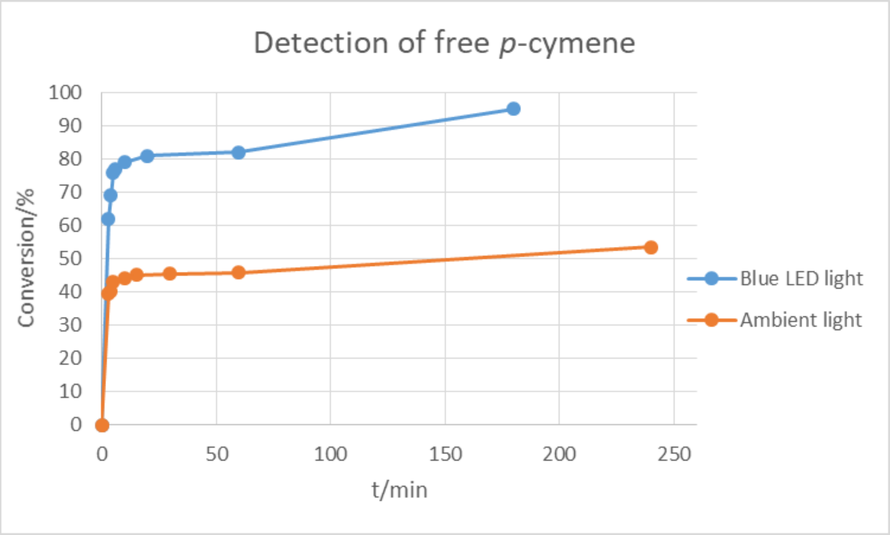

Ambient light		Blue LED light	
t/min	**[7]**,mg/ml	t/min	**[7]**,mg/ml
3	3.17	1	4.99
4	3.24	2	5.49
5	3.46	4	6.09
10	3.55	5	6.17
15	3.61	10	6.39
30	3.65	20	6.46
60	3.68	60	6.54
240	4.29	180	7.65

Spectroscopic Data

Scheme 2 products:

2-(4'-Methoxy-[1,1'-biphenyl]-2-yl)pyridine (5a) [1]

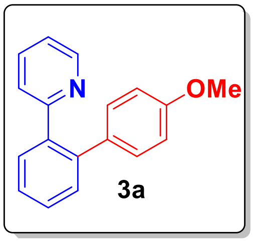

3a

White solid; ^{1}**H NMR** (400 MHz, $CDCl_3$) δ 8.64 (ddd, J = 4.9, 1.8, 0.9 Hz, 1H), 7.71 – 7.64 (m, 1H), 7.49 – 7.36 (m, 4H), 7.14 – 7.04 (m, 3H), 6.90 (dt, J = 8.0, 1.1 Hz, 1H), 6.81 – 6.74 (m, 2H), 3.79 (s, 3H). ^{13}**C NMR** (100 MHz, $CDCl_3$) δ 159.6, 158.6, 149.6, 140.3, 139.5, 135.4, 133.8, 130.9, 130.62, 130.56, 128.6, 127.4, 125.6, 121.4, 113.7, 55.3 (q, J = 5.0 Hz). **HRMS** (ESI) Calcd for $[C_{18}H_{15}ON+Na]^+$ 284.1046, Found 284.1038.

2-(4,4''-Dimethoxy-[1,1':3',1''-terphenyl]-2'-yl)pyridine (5aa) [2]

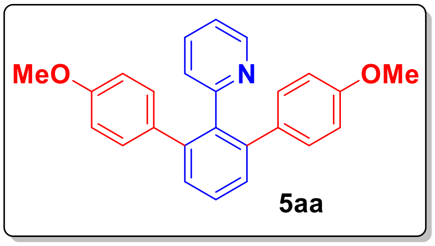

5aa

Off-white solid; ^{1}**H NMR** (400 MHz, $CDCl_3$) δ 8.35 (ddd, J = 4.9, 1.8, 1.0 Hz, 1H), 7.48 (dd, J = 8.5, 6.6 Hz, 1H), 7.43 – 7.37 (m, 2H), 7.33 (td, J = 7.7, 1.8 Hz, 1H), 7.04 – 6.98 (m, 4H), 6.93 (ddd, J = 7.6, 4.9, 1.2 Hz, 1H), 6.88 (dt, J = 7.8, 1.1 Hz, 1H), 6.72 – 6.66 (m, 4H), 3.74 (s, 6H). ^{13}**C NMR** (100 MHz, $CDCl_3$) δ 159.4, 158.2, 148.7, 141.5, 138.6, 135.1, 134.2, 130.8, 129.3, 128.2, 126.9, 120.9, 113.2, 55.3. **HRMS** (ESI) Calcd for $[C_{25}H_{21}O_2N+Na]^+$ 390.1465, Found 390.1455.

2-(4',5-Dimethoxy-[1,1'-biphenyl]-2-yl)pyridine (5b) [1,3]

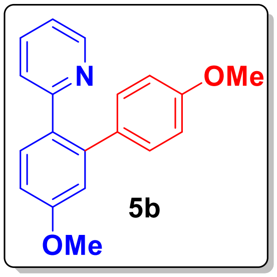

5b

Colorless pasty solid; ^{1}**H NMR** (400 MHz, $CDCl_3$) δ 8.61 (ddd, J = 4.8, 1.9, 1.0 Hz, 1H), 7.64 (dd, J = 8.5, 1.6 Hz, 1H), 7.38 – 7.34 (m, 1H), 7.10 – 7.04 (m, 3H), 6.99 (dt, J = 8.5, 2.2 Hz, 1H), 6.93 (t, J = 2.5 Hz, 1H), 6.84 – 6.81 (m, 1H), 6.80 – 6.77 (m, 2H), 3.88 (d, J = 1.8 Hz, 3H), 3.79 (d, J = 1.9 Hz, 3H). ^{13}**C NMR** (100 MHz, $CDCl_3$) δ 159.6, 159.1, 158.6, 149.3, 141.5, 135.1, 133.7, 132.2, 131.9, 130.7, 125.4, 120.8, 115.6, 113.5, 112.9, 55.4, 55.2. **HRMS** (ESI) Calcd for $[C_{19}H_{18}O_2N+K]^+$ 292.1320, Found 292.1332.

---

[1] C. Binnani, R. K. Rai, D. Tyagi, S. M. Mobin and S. K. Singh, *Eur. J. Inorg. Chem.*, **2018**, *12*, 1435-1445.
[2] C. K. Rank, T. Wall, F. Dietrich, M. Vidovic, M. P. Klein, Y. Sun, G. Niedner-Schatteburg, M. Gerhards and F. W. Patureau, *Eur. J. Inorg. Chem.*, **2018**, *12*, 1394-1398.
[3] L. Su, D.-D. Guo, B. Li, S.-H. Guo, G.-F. Pan, Y.-R. Gao and Y.-Q. Wang, Chem. *ChemCatChem.*, **2017**, *9*, 2001-2008.

2-(4,4'',5'-Trimethoxy-[1,1':3',1''-terphenyl]-2'-yl)pyridine (5bb) [1,4]

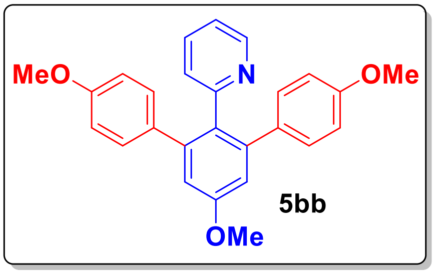

White solid; **^{1}H NMR** (400 MHz, $CDCl_3$) δ 8.32 (ddd, J = 4.9, 1.8, 0.9 Hz, 1H), 7.30 (td, J = 7.7, 1.8 Hz, 1H), 7.03 – 7.00 (m, 4H), 6.94 (s, 2H), 6.90 (ddd, J = 7.7, 4.9, 1.1 Hz, 1H), 6.84 (d, J = 7.9, 1H), 6.71 – 6.67 (m, 4H), 3.89 (s, 3H), 3.74 (s, 6H). **^{13}C NMR** (100 MHz, $CDCl_3$) δ 159.1, 158.8, 158.1, 148.5, 142.8, 134.9, 134.1, 131.6, 130.5, 127.1, 120.5, 114.5, 113.0, 55.4, 55.1. **HRMS** (ESI) Calcd for $[C_{26}H_{23}O_3N+K]^+$ 397.1662, Found 397.1672.

2-(5-(Tert-butyl)-4'-methoxy-[1,1'-biphenyl]-2-yl)pyridine (5c) [1,4]

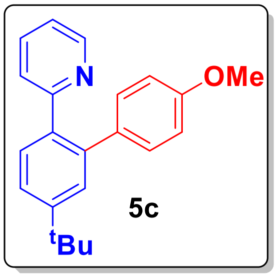

Pasty light yellow oil; **^{1}H NMR** (500 MHz, $CDCl_3$) δ 8.62 (d, J = 4.7 Hz, 1H), 7.62 (d, J = 8.1 Hz, 1H), 7.47 (d, J = 8.1 Hz, 1H), 7.41 – 7.37 (m, 2H), 7.10 – 7.07 (m, 3H), 6.89 (d, J = 7.9 Hz, 1H), 6.79 (d, J = 8.2 Hz, 2H), 3.79 (s, 3H), 1.38 (s, 9H). **^{13}C NMR** (125 MHz, $CDCl_3$) δ 159.4, 158.4, 151.5, 149.4, 139.7, 136.6, 135.1, 134.3, 130.8, 130.2, 127.5, 125.3, 124.5, 121.0, 113.5, 55.2, 34.7, 31.3. **HRMS** (ESI) Calcd for $[C_{22}H_{23}ON+K]^+$ 356.1398, Found 356.1411.

2-(5'-(Tert-butyl)-4,4''-dimethoxy-[1,1':3',1''-terphenyl]-2'-yl)pyridine (5cc) [1,4]

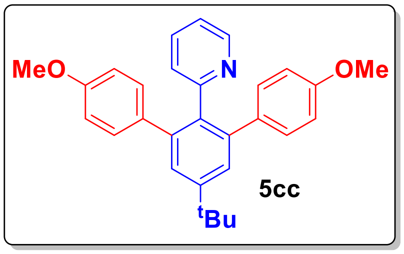

White solid; **^{1}H NMR** (500 MHz, $CDCl_3$) δ 8.35 (d, J = 4.4 Hz, 1H), 7.43 (s, 2H), 7.32 (t, J = 7.6 Hz, 1H), 7.04 (d, J = 8.3 Hz, 4H), 6.91 (t, J = 8.4 Hz, 2H), 6.71 (d, J = 8.3 Hz, 4H), 3.75 (s, 6H), 1.42 (s, 9H).). **^{13}C NMR** (125 MHz, $CDCl_3$) δ 159.4, 157.9, 150.9, 148.5, 140.9, 135.7, 134.8, 134.6, 130.7, 126.7, 126.4, 120.6, 113.0, 55.1, 34.7, 31.3. **HRMS (ESI)** Calcd for $[C_{29}H_{29}O_2N+K]^+$ 462.1816, Found 462.1830.

[4] T.-T. Zhao, W.-H. Xu, Z.-J. Zheng, P.-F. Xu and H. Wei, *J. Am. Chem. Soc.*, **2018**, *140*, 586-589.

2-(4'-Methoxy-5-methyl-[1,1'-biphenyl]-2-yl)pyridine (5d) [1,4]

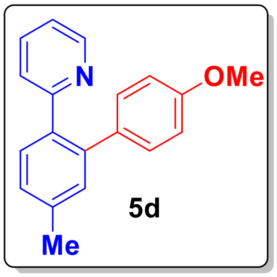

Yellow oil; ^{1}**H NMR** (400 MHz, $CDCl_3$) δ 8.63 (d, J = 4.7 Hz, 1H), 7.59 (d, J = 7.8 Hz, 1H), 7.37 (td, J = 7.7, 1.9 Hz, 1H), 7.26 – 7.19 (m, 2H), 7.11 – 7.03 (m, 3H), 6.91 – 6.83 (m, 1H), 6.82 – 6.73 (m, 2H), 3.78 (s, 3H), 2.44 (s, 3H). ^{13}**C NMR** (100 MHz, $CDCl_3$) δ 159.6, 158.6, 149.5, 140.1, 138.4, 136.8, 135.3, 134.0, 131.3, 130.9, 130.6, 128.2, 125.5, 121.2, 113.6, 55.3, 21.4. **HRMS** (ESI) Calcd for $[C_{19}H_{17}NO+Na]^+$ 298.1192, found 298.1202.

2-(4,4''-Dimethoxy-5'-methyl-[1,1':3',1''-terphenyl]-2'-yl)pyridine (5dd) [1,4]

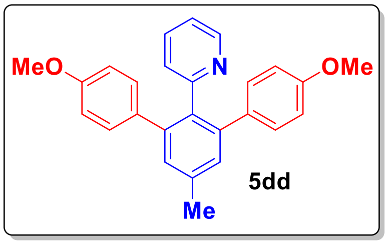

Off-white solid; ^{1}**H NMR** (400 MHz, $CDCl_3$) δ 8.34 (d, J = 4.4 Hz, 1H), 7.35 – 7.28 (m, 1H), 7.23 (s, 2H), 7.05 – 6.97 (m, 4H), 6.91 (ddd, J = 7.6, 4.9, 1.2 Hz, 1H), 6.86 (dd, J = 7.8, 1.2 Hz, 1H), 6.72 – 6.65 (m, 4H), 3.74 (s, 6H), 2.46 (s, 3H). ^{13}**C NMR** (100 MHz, $CDCl_3$) δ 159.5, 158.1, 148.7, 141.4, 137.8, 135.9, 135.0, 134.3, 130.8, 130.0, 127.0, 120.8, 113.2, 55.2, 21.4. **HRMS** (ESI) Calcd for $[C_{26}H_{23}NO_2+Na]^+$ 404.1604, found 404.1621.

2-(4,4''-Dimethoxy-5'-phenyl-[1,1':3',1''-terphenyl]-2'-yl)pyridine (5ee) [1,4]

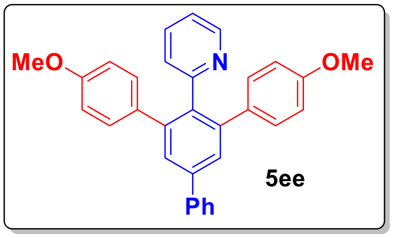

Light yellow solid; ^{1}**H NMR** (400 MHz, $CDCl_3$) δ 8.40 – 8.38 (m, 1H), 7.72 (d, J = 7.4 Hz, 2H), 7.673 (s, 1H), 7.671 (s, 1H), 7.49 – 7.45 (m, 2H), 7.41 – 7.34 (m, 2H), 7.11 – 7.07 (m, 4H), 7.00 – 6.93 (m, 2H), 6.80 – 6.68 (m, 4H), 3.76 (s, 6H).^{13}**C NMR** (100 MHz, $CDCl_3$) δ 159.0, 158.1, 148.6, 141.9, 140.8, 140.5, 137.4, 135.0, 134.0, 130.6, 128.8, 127.9, 127.5, 127.2, 126.8, 120.8, 113.1, 55.1. **HRMS (ESI)** Calcd for $[C_{31}H_{25}O_2N+K]^+$ 482.1503, Found 482.1517.

Methyl 4,4''-dimethoxy-2'-(pyridin-2-yl)-[1,1':3',1''-terphenyl]-5'-carboxylate (5ff) [1,4]

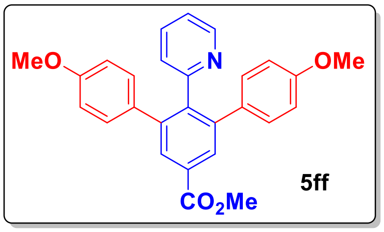

Light yellow solid; ^{1}**H NMR** (500 MHz, $CDCl_3$) δ 8.28 (d, J = 4.8 Hz, 1H), 8.00 (s, 2H), 7.26 (td, J = 7.7, 1.8 Hz, 1H), 6.95 – 6.92 (m, 4H), 6.87 (ddd, J = 7.6, 4.9, 1.2 Hz, 1H), 6.79 (d, J = 7.8, 1H), 6.62 – 6.61 (m, 4H), 3.86 (s, 3H), 3.65 (s, 6H). ^{13}**C NMR** (125 MHz, $CDCl_3$) δ 166.8, 158.4, 158.3, 148.7, 142.5, 141.8, 135.1, 133.0, 130.6, 130.1, 129.6, 126.4, 121.2, 113.2, 55.1, 52.2. **HRMS** (ESI) Calcd for $[C_{22}H_{17}ON+Na]^+$ 448.1501, Found 448.1519.

2-(4,4'-Dimethoxy-[1,1'-biphenyl]-2-yl)pyridine (5g) [1,4]

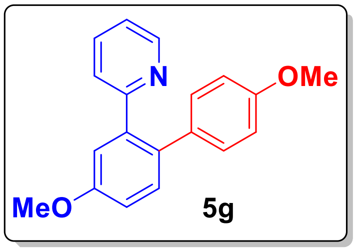

Light yellow oil; **^{1}H NMR** (400 MHz, $CDCl_3$) δ 8.65 (ddd, J = 4.9, 1.8, 1.0 Hz, 1H), 7.39 (td, J = 7.7, 1.8 Hz, 1H), 7.33 (d, J = 8.5 Hz, 1H), 7.23 (d, J = 2.7 Hz, 1H), 7.11 (ddd, J = 7.5, 4.9, 1.1 Hz, 1H), 7.05 – 6.99 (m, 3H), 6.89 (dt, J = 7.9, 1.1 Hz, 1H), 6.79 – 6.73 (m, 2H), 3.89 (s, 3H), 3.77 (s, 3H). **^{13}C NMR** (100 MHz, $CDCl_3$) δ 159.2, 158.8, 158.1, 149.3, 140.3, 135.2, 133.4, 132.8, 131.6, 130.7, 125.4, 121.4, 115.0, 114.8, 113.4, 55.4, 55.1. **HRMS (ESI)** Calcd for $[C_{19}H_{17}O_2N+K]^+$ 330.0875, Found 330.0891.

2-(4'-Methoxy-4-methyl-[1,1'-biphenyl]-2-yl)pyridine (5h) [1,4]

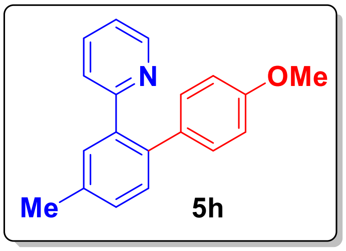

Yellow solid; **^{1}H NMR** (500 MHz, $CDCl_3$) δ 8.64 – 8.63 (m, 1H), 7.54 (s, 1H), 7.37 (td, J = 7.7, 1.9 Hz, 1H), 7.30 (d, J = 7.8 Hz, 1H), 7.26 – 7.24 (m, 1H), 7.08 (ddd, J = 7.5, 4.9, 1.2 Hz, 1H), 7.06 – 7.03 (m, 2H), 6.87 (d J = 7.9 Hz, 1H), 6.77 – 6.74 (m, 2H), 3.76 (s, 3H), 2.43 (s, 3H). **^{13}C NMR** (125 MHz, $CDCl_3$) δ 159.5, 158.3, 149.4, 139.1, 137.3, 137.0, 135.1, 133.6, 131.0, 130.7, 130.3, 129.2, 125.4, 121.2, 113.5, 55.1, 21.0. **HRMS** (ESI) Calcd for $[C_{19}H_{17}ON+K]^+$ 314.0927, Found 314.0942.

2-(4-Chloro-4'-methoxy-[1,1'-biphenyl]-2-yl)pyridine (5i) [1,4]

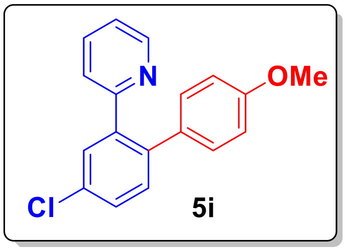

Light yellow solid; **^{1}H NMR** (500 MHz, $CDCl_3$) δ 8.61 – 8.51 (m, 1H), 7.65 – 7.56 (m, 1H), 7.36 – 7.29 (m, 2H), 7.25 (d, J = 8.2 Hz, 1H), 7.08 – 7.00 (m, 1H), 6.95 (d, J = 8.2 Hz, 2H), 6.79 (d, J = 7.9 Hz, 1H), 6.69 (d, J = 8.3 Hz, 2H), 3.69 (s, 3H). **^{13}C NMR** (125 MHz, $CDCl_3$) δ 158.7, 158.0, 149.5, 140.7, 138.6, 135.4, 133.2, 132.4, 131.7, 130.6, 130.4, 128.4, 125.2, 121.7, 113.6, 55.2. **HRMS (ESI)** Calcd for $[C_{18}H_{14}ON^{35}Cl+K]^+$ 334.0382, Found 334.0396.

2-(4'-Methoxy-[1,1'-biphenyl]-2-yl)-4-methylpyridine (5j) [1,4]

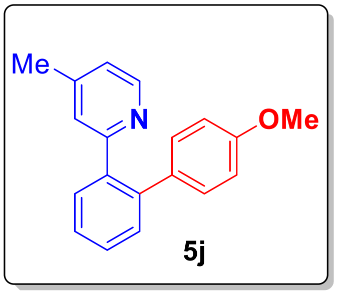

Light yellow oil; **^{1}H NMR** (500 MHz, $CDCl_3$) δ 8.47 (d, J = 5.0 Hz, 1H), 7.64 – 7.63 (m, 1H), 7.44 – 7.39 (m, 3H), 7.08 – 7.06 (m, 2H), 6.93 (d, J = 4.6, 1H), 6.78 – 6.75 (m, 3H), 3.79 (s, 3H), 2.13 (s, 3H). **^{13}C NMR** (125 MHz, $CDCl_3$) δ 159.2, 158.4, 149.0, 146.2, 140.2, 139.5, 133.8, 130.7, 130.4, 130.3, 128.3, 127.2, 126.2, 122.3, 113.4, 55.2, 20.9. **HRMS (ESI)**

Calcd for $[C_{19}H_{17}ON+K]^+$ 314.0928, Found 314.0942.

2-(4,4''-Dimethoxy-[1,1':3',1''-terphenyl]-2'-yl)-4-methylpyridine (5jj) [1,4]

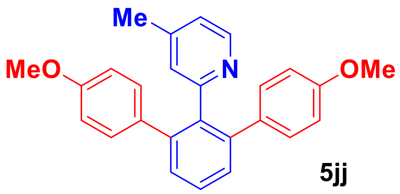

5jj

Colourless oil; ^{1}**H NMR** (400 MHz, $CDCl_3$) δ 8.23 (dd, J = 5.0, 0.8 Hz, 1H), 7.48 (dd, J = 8.6, 6.5 Hz, 1H), 7.43 – 7.38 (m, 2H), 7.09 – 7.02 (m, 4H), 6.79 – 6.75 (m, 1H), 6.75 – 6.69 (m, 5H), 3.76 (s, 6H), 2.10 (s, 3H).^{13}**C NMR** (100 MHz, $CDCl_3$) δ 158.8, 157.9, 148.1, 145.7, 141.3, 138.4, 134.1, 130.5, 129.0, 127.9, 127.6, 121.8, 112.9, 55.0, 20.8. **HRMS** (ESI) Calcd for $[C_{26}H_{23}O_2N+K]^+$ 420.134, Found 420.1360.

2-(4'-Methoxy-[1,1'-biphenyl]-2-yl)-5-methylpyridine (5k) [1,4]

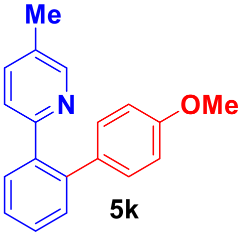

5k

Yellow oil; ^{1}**H NMR** (400 MHz, $CDCl_3$) δ 8.47 (s, 1H), 7.67 – 7.65 (m, 1H), 7.45 – 7.38 (m, 3H), 7.21 (dd, J = 8.1, 2.3 Hz, 1H), 7.10 – 7.06 (m, 2H), 6.80 – 6.76 (m, 3H), 3.80 (s, 3H), 2.30 (s, 3H). ^{13}**C NMR** (100 MHz, $CDCl_3$) δ 158.4, 156.5, 149.8, 140.1, 139.3, 135.9, 133.9, 130.7, 130.6, 130.4, 130.3, 128.2, 127.3, 124.8, 113.5, 55.2, 18.1. **HRMS** (ESI) Calcd for $[C_{19}H_{17}ON+K]^+$ 314.0927, Found 314.0942.

2-(4,4''-Dimethoxy-[1,1':3',1''-terphenyl]-2'-yl)-5-methylpyridine (5kk) [1,4]

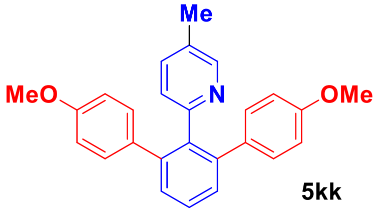

5kk

Yellow solid; ^{1}**H NMR** (400 MHz, $CDCl_3$) δ 8.20 (s, 1H), 7.48 – 7.44 (m, 1H), 7.40 (s, 1H), 7.38 – 7.37 (m, 1H), 7.14 (dd, J = 8.0, 2.3 Hz, 1H), 7.04 – 7.00 (m, 4H), 6.77 (d, J = 7.9 Hz, 1H), 6.74 – 6.66 (m, 4H), 3.75 (s, 6H), 2.20 (s, 3H). ^{13}**C NMR** (100 MHz, $CDCl_3$) δ 157.9, 156.1, 148.9, 141.4, 138.3, 135.6, 134.2, 130.6, 129.9, 129.1, 127.9, 126.1, 113.0, 55.1, 18.1. **HRMS** (ESI) Calcd for $[C_{26}H_{23}O_2N+K]^+$ 420.1346, Found 420.1360.

5-Methyl-2-(4,4'',5'-trimethoxy-[1,1':3',1''-terphenyl]-2'-yl)pyridine (5ll)

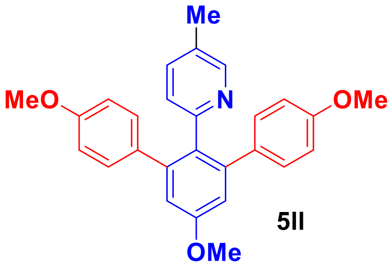

5ll

Light yellow solid; ^{1}**H NMR** (400 MHz, $CDCl_3$) δ 8.16 – 8.15 (m, 1H), 7.11 (dd, J = 7.9, 1.8 Hz, 1H), 7.04 – 7.01 (m, 4H), 6.94 (s, 2H), 6.73 (d, J = 8.0 Hz, 1H), 6.72 – 6.68 (m, 4H), 3.88 (s, 3H), 3.75 (s, 6H), 2.18 (s, 3H).^{13}**C NMR** (100 MHz, $CDCl_3$) δ 158.6, 158.0, 156.0, 148.8, 142.8, 135.6, 134.2, 131.4, 130.5, 129.6, 126.4, 114.5,

113.0, 55.3, 55.0, 18.1. **HRMS (ESI)** Calcd for $[C_{27}H_{25}O_3N+K]^+$ 450.1449, Found 450.1466.

2-(3-(4-Methoxyphenyl)naphthalen-2-yl)pyridine (5m)

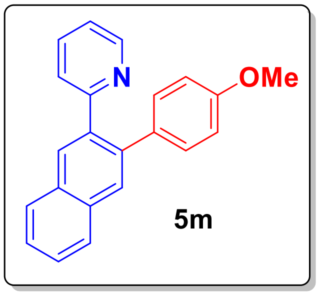

White solid; **^{1}H NMR** (400 MHz, $CDCl_3$) δ 8.69 (ddd, J = 4.9, 1.9, 1.0 Hz, 1H), 8.19 (s, 1H), 7.94 – 7.92 (m, 1H), 7.90 – 7.87 (m, 2H), 7.54 – 7.47 (m, 2H), 7.43 (td, J = 7.7, 1.9 Hz, 1H), 7.20 – 7.16 (m, 2H), 7.14 (ddd, J = 7.5, 4.9, 1.2 Hz, 1H), 6.98 (dt, J = 7.9, 1.1 Hz, 1H), 6.84 – 6.80 (m, 2H), 3.80 (s, 3H). **^{13}C NMR** (100 MHz, $CDCl_3$) δ 159.2, 158.5, 149.4, 138.1, 137.8, 135.2, 133.7, 133.2, 132.4, 130.8, 130.0, 129.2, 128.2, 127.5, 126.6, 126.1, 125.5, 121.3, 113.5, 55.2. **HRMS (ESI)** Calcd for $[C_{22}H_{17}ON+K]^+$ 350.0927, Found 350.0942.

2-(4'-Methoxy-[1,1'-biphenyl]-2-yl)quinoline (5n)

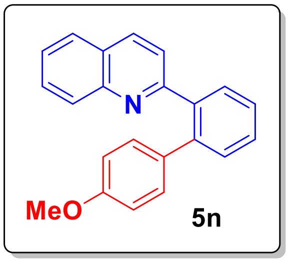

Yellow oil; **^{1}H NMR** (500 MHz, $CDCl_3$) δ 8.54 (d, J = 5.7 Hz, 1H), 7.72 (d, J = 8.3, 1H), 7.60 – 7.45 (m, 7H), 7.32 – 7.26 (m, 1H), 7.02 – 6.99 (m, 2H), 6.65 – 6.53 (m, 2H), 3.61 (s, 3H). **^{13}C NMR** (125 MHz, $CDCl_3$) δ 161.5, 158.1, 141.9, 141.1, 138.1, 136.0, 133.5, 130.6, 130.1, 129.8, 129.7, 128.7, 127.5, 127.2, 126.8, 126.7, 126.5, 119.7, 113.2, 54.93. **HRMS (ESI)** Calcd for $[C_{22}H_{17}ON+K]^+$ 350.0929, Found 350.0942.

1-(4'-Methoxy-[1,1'-biphenyl]-2-yl)isoquinoline (5o):

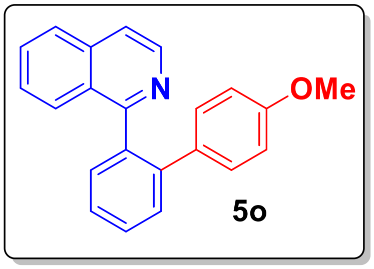

Yellow solid; **^{1}H NMR** (400 MHz, $CDCl_3$) δ 8.54 (d, J = 5.7 Hz, 1H), 7.73 (d, J = 8.3 Hz, 1H), 7.65 – 7.42 (m, 7H), 7.31 (t, J = 7.7 Hz, 1H), 7.00 (d, J = 8.2 Hz, 2H), 6.54 (d, J = 8.2 Hz, 2H), 3.63 (s, 3H). **^{13}C NMR** (100 MHz, $CDCl_3$) δ 161.6, 158.3, 142.1, 141.3, 138.2, 136.1, 133.6, 130.8, 130.2, 130.0, 129.9, 128.9, 127.6, 127.3, 127.0, 126.9, 126.7, 119.9, 113.3, 55.1 (d, J = 5.6 Hz). **HRMS** (ESI) Calcd for $[C_{22}H_{17}ON+H]^+$ 312.1383, Found 312.1374.

2-(3-(4-Methoxyphenyl)thiophen-2-yl)pyridine (5p)

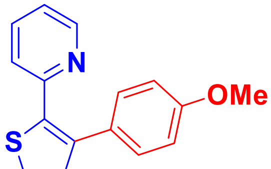

5p

Yellow oil; **^{1}H NMR** (500 MHz, $CDCl_3$) δ 8.58 (ddd, J = 4.9, 1.9, 1.1 Hz, 1H), 7.42 – 7.39 (m, 2H), 7.30 – 7.27 (m, 2H), 7.09 – 7.05 (m, 3H), 6.93 – 6.90 (m, 2H), 3.85 (s, 3H). **^{13}C NMR** (125 MHz, $CDCl_3$) δ 159.0, 153.1, 149.5, 139.6, 138.7, 135.8, 131.2, 130.2, 129.3, 126.4, 121.8. 121.5, 114.1, 55.3. **HRMS (ESI)** Calcd for $[C_{16}H_{13}ONS+K]^+$ 306.0336, Found 306.0349.

1-(4'-Methoxy-[1,1'-biphenyl]-2-yl)-1H-pyrazole (5q)[5,6]

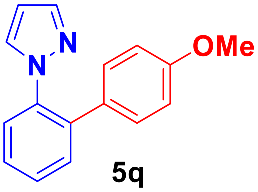

5q

Colourless oil; **^{1}H NMR** (500 MHz, $CDCl_3$) δ 7.64 (d, J = 1.9 Hz, 1H), 7.62 – 7.55 (m, 1H), 7.49 – 7.39 (m, 3H), 7.10 (d, J = 2.4 Hz, 1H), 7.07 – 6.98 (m, 2H), 6.85 – 6.78 (m, 2H), 3.80 (s, 3H). **^{13}C NMR** (125 MHz, $CDCl_3$) δ 159.1, 140.3, 138.7, 136.5, 131.5, 131.1, 131.0, 129.8, 128.4, 128.1, 126.8, 114.1, 106.5 (d, J = 1.9 Hz), 55.4 (q, J = 4.9 Hz). **HRMS** (APCI) Calcd for $[C_{16}H_{14}ON_2+H]^+$ 251.1179, Found 251.1168.

1-(4,4''-Dimethoxy-[1,1':3',1''-terphenyl]-2'-yl)-1H-pyrazole (5qq)[7]

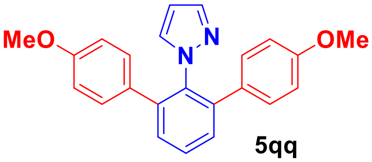

5qq

White solid (46 mg, 52%). ^{1}H NMR (400 MHz, $CDCl_3$) δ 7.57 – 7.48 (m, 1H), 7.48 – 7.37 (m, 3H), 7.09 (s, 1H), 7.02 (d, J = 8.2 Hz, 4H), 6.76 (d, J = 8.3 Hz, 4H), 6.09 (s, 1H), 3.77 (s, 6H). ^{13}C NMR (100 MHz, $CDCl_3$) δ 158.9, 140.2, 139.5, 136.4, 132.5, 131.3, 129.8, 129.5, 129.2, 113.6, 106.2, 55.3. HRMS (APCI) Calcd for $[C_{23}H_{20}O_2N_2+H]^+$ 357.1598, Found 357.1587.

---

[5] P. B. Arockiam, C. Fischmeister, C. Bruneau and P. H. Dixneuf, *Green Chem.*, **2013**, *15*, 67-71.
[6] L. Ackermann, R, Vicente, H. K. Potukuchi and V. Pirovano, *Org. Lett.*, **2010**, *12*, 5032-5035.
[7] L. Ackermann, A. Althammer and R. Born, *Tetrahedron*, **2008**, *64*, 6115-6124.

Scheme 3 products:

2-(4'-(Tert-butyl)-[1,1'-biphenyl]-2-yl)-3-methylpyridine (6a)[8]

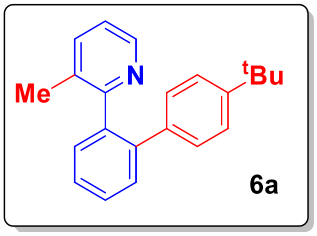

Colorless oil; ^{1}**H NMR** (400 MHz, $CDCl_3$) δ 8.51 (d, J = 4.7 Hz, 1H), 7.49 – 7.36 (m, 4H), 7.30 (d, J = 7.8 Hz, 1H), 7.18 (d, J = 8.4, 1.8 Hz, 2H), 7.14 – 7.08 (m, 1H), 7.04 (d, J = 7.3 Hz, 2H), 1.73 (s, 3H), 1.26 (s, 9H). ^{13}**C NMR** (100 MHz, $CDCl_3$) δ 159.7, 149.6, 146.6, 140.6, 139.5, 138.1, 137.5, 131.8, 130.1, 129.8, 128.9, 128.4, 127.3, 124.8, 122.2, 34.5, 31.4, 19.0. **HRMS** (ESI) Calcd for $(C_{22}H_{23}N+H)^+$ 302.1903, Found 302.1897.

3-Methyl-2-(4'-methyl-[1,1'-biphenyl]-2-yl)pyridine (6b)

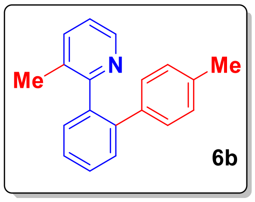

Yellow oil; ^{1}**H NMR** (400 MHz, $CDCl_3$) δ 8.51 (d, J = 4.9 Hz, 1H), 7.50 – 7.36 (m, 4H), 7.30 (d, J = 7.6 Hz, 1H), 7.10 (dd, J = 7.7, 4.8 Hz, 1H), 6.99 (q, J = 8.0 Hz, 4H), 2.27 (s, 3H), 1.75 (s, 3H). ^{13}**C NMR** (125 MHz, $CDCl_3$) δ 159.8, 146.7, 140.7, 139.5, 138.3, 137.6, 136.4, 131.8, 130.0, 129.8, 129.2, 128.7, 128.4, 127.3, 122.2, 21.2, 19.0. **HRMS** (APCI) Calcd for $[C_{19}H_{17}N+H]^+$ 260.1434, Found 260.1428.

2-([1,1':4',1'':3'',1''':4''',1''''-Quinquephenyl]-2''-yl)pyridine (6cc)

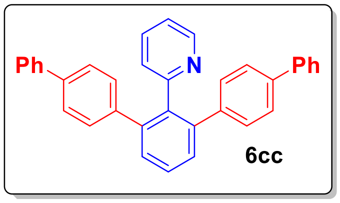

White soild; ^{1}**H NMR** (400 MHz, $CDCl_3$) δ 8.44 – 8.32 (m, 1H), 7.62 – 7.58 (m, 7H), 7.46 – 7.42 (m, 8H), 7.38 – 7.33 (m, 3H), 7.22 – 7.21 (m, 4H), 7.00 – 6.95 (m, 2H).^{13}**C NMR** (100 MHz, $CDCl_3$) δ 158.9, 148.6, 141.5, 140.6, 140.5, 138.9, 138.5, 135.0, 130.0, 129.5, 128.7, 128.3, 127.2, 126.9, 126.8, 126.3, 121.0. **HRMS (ESI)** Calcd for $[C_{35}H_{25}N+K]^+$ 498.1602, Found 498.1619.

2-(2,6-Di(thiophen-2-yl)phenyl)pyridine (6dd)

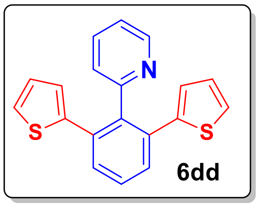

White soild; ^{1}**H NMR** (400 MHz, $CDCl_3$) δ 8.56 (ddd, J = 4.9, 1.8, 1.0 Hz, 1H), 7.59 – 7.57 (m, 2H), 7.52 – 7.44 (m, 2H), 7.16 – 7.13 (m, 3H), 7.09 (dt, J = 7.7, 1.1 Hz, 1H), 6.82 (dd, J = 5.1, 3.6 Hz, 2H), 6.65 (dd, J = 3.6, 1.2 Hz, 2H).^{13}**C NMR** (100 MHz, $CDCl_3$) δ 158.5, 148.8, 142.6, 138.4, 135.7, 134.6, 130.0, 128.4,

[8] C. K. Seigerman, T. M. Micyus, S. R. Neufeldt and M. S. Sanford, *Tetrahedron*, **2013**, *69*, 5580-5587.

127.1, 126.7, 126.2, 125.7, 122.1. **HRMS (ESI)** Calcd for $[C_{19}H_{13}NS_2+K]^+$ 358.0106, Found 358.0121.

2-(4'-Chloro-[1,1'-biphenyl]-2-yl)-3-methylpyridine (6e)[9]

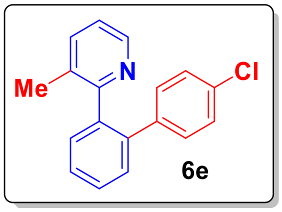

Yellow solid; ^{1}**H NMR** (500 MHz, $CDCl_3$) δ 8.49 (dd, J = 4.8, 1.5 Hz, 1H), 7.48 – 7.44 (m, 2H), 7.44 – 7.37 (m, 2H), 7.33 (dd, J = 7.8, 1.6 Hz, 1H), 7.17 – 7.09 (m, 3H), 7.08 – 6.99 (m, 2H), 1.77 (s, 3H). ^{13}**C NMR** (100 MHz, $CDCl_3$) δ 159.2, 146.8, 139.7, 139.5 (d, J = 2.1 Hz), 137.8, 132.9, 131.6, 130.6, 130.1, 129.6, 128.6, 128.2, 127.9, 122.4, 19.0. **HRMS** (APCI) Calcd for $[C_{18}H_{14}N^{35}Cl+H]^+$ 280.0888, Found 280.0881.

2-(4'-Bromo-[1,1'-biphenyl]-2-yl)-3-methylpyridine (6f)

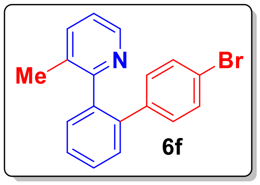

Yellow solid; ^{1}**H NMR** (500 MHz, $CDCl_3$) δ 8.48 (dd, J = 4.8, 1.7 Hz, 1H), 7.50 – 7.43 (m, 2H), 7.43 – 7.37 (m, 2H), 7.33 (dd, J = 7.8, 1.6 Hz, 1H), 7.31 – 7.26 (m, 2H), 7.12 (dd, J = 7.7, 4.8 Hz, 1H), 7.01 – 6.95 (m, 2H), 1.77 (s, 3H). ^{13}**C NMR** (100 MHz, $CDCl_3$) δ 159.2, 146.8, 140.2, 139.51, 139.46, 137.8, 131.6, 131.1, 131.0, 130.1, 129.6, 128.6, 127.9, 122.4, 121.2, 19.0. **HRMS** (ESI) Calcd for $[C_{18}H_{14}N^{79}Br+H]^+$ 324.0382, Found 324.0377.

2-(4,4''-Difluoro-[1,1':3',1''-terphenyl]-2'-yl)pyridine (6gg)

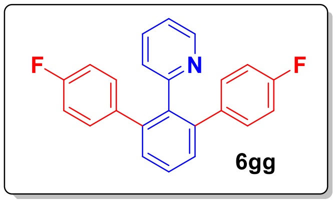

White solid; ^{1}**H NMR** (400 MHz, , $CDCl_3$) δ 8.34 (ddd, J = 4.9, 1.8, 1.0 Hz, 1H), 7.51 (dd, J = 8.5, 6.8 Hz, 1H), 7.43 – 7.41 (m, 2H), 7.34 (td, J = 7.7, 1.8 Hz, 1H), 7.08 – 7.02 (m, 4H), 6.95 (ddd, J = 7.6, 4.9, 1.2 Hz, 1H), 6.88 – 6.80 (m, 5H). ^{13}**C NMR** (125 MHz, $CDCl_3$) δ 161.5 (d, $J_{C\text{-}F}$ = 244.3 Hz), 158.6, 148.7, 140.8, 138.6, 137.3 (d, $J_{C\text{-}F}$ = 3.4 Hz), 135.1, 131.1 (d, $J_{C\text{-}F}$ = 7.9 Hz), 129.5, 128.3, 126.7, 121.1, 114.6 (d, $J_{C\text{-}F}$ = 21.1 Hz). **HRMS** (ESI) Calcd for $[C_{23}H_{16}NF_2+K]^+$ 344.1240, Found 344.1245.

---

[9] D. Kalyani, N. R. Deprez, L. V. Desai and M. S. Sanford, *J. Am. Chem. Soc.*, **2005**, *127*, 7330-7331.

1-(2'-(3-Methylpyridin-2-yl)-[1,1'-biphenyl]-4-yl)ethan-1-one (6h)[10]

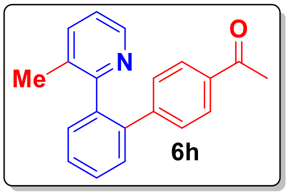

6h

Yellow-brown solid; ^{1}**H NMR** (400 MHz, $CDCl_3$) δ 8.51 – 8.45 (m, 1H), 7.81 – 7.70 (m, 2H), 7.55 – 7.39 (m, 4H), 7.32 (dd, J = 7.6, 0.9 Hz, 1H), 7.24 – 7.18 (m, 2H), 7.11 (dd, J = 7.7, 4.8 Hz, 1H), 2.54 (s, 3H), 1.78 (s, 3H). ^{13}**C NMR** (125 MHz, acetone-d_6) δ 197.6, 160.1, 147.6, 146.9, 141.0, 140.6, 138.4, 136.5, 132.2, 131.1, 130.6, 130.3, 129.4, 128.9, 128.8, 123.4, 26.8 (q, J = 4.4 Hz), 19.2 (d, J = 1.4 Hz). HRMS (ESI) Calcd for $[C_{20}H_{17}NO+H]^+$ 288.1383, Found 288.1373. **HRMS** (ESI) Calcd for $[C_{20}H_{17}NO+H]^+$ 288.1383, Found 288.1373.

Ethyl 2'-(3-methylpyridin-2-yl)-[1,1'-biphenyl]-4-carboxylate (6i)

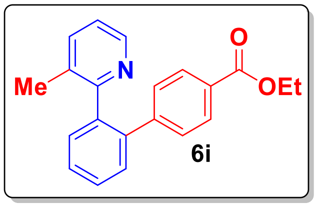

6i

Yellow-brown solid; ^{1}**H NMR** (400 MHz, $CDCl_3$) δ 8.47 (d, J = 4.8 Hz, 1H), 7.84 (d, J = 7.9 Hz, 2H), 7.56 – 7.37 (m, 4H), 7.29 (d, J = 7.7 Hz, 1H), 7.18 (d, J = 8.0 Hz, 2H), 7.10 (dd, J = 7.7, 4.9 Hz, 1H), 4.32 (q, J = 7.1 Hz, 2H), 1.75 (s, 3H), 1.35 (t, J = 7.1 Hz, 3H). ^{13}**C NMR** (100 MHz, $CDCl_3$) δ 166.6, 159.0, 146.7, 145.8, 139.7, 139.5, 137.8, 131.6, 130.1, 129.7, 129.3, 129.2, 128.7, 128.6, 128.2, 122.4, 61.0, 18.9, 14.4 (d, J = 3.3 Hz). **HRMS** (ESI) Calcd for $[C_{21}H_{19}O_2N+H]^+$ 340.1308, Found 340.1295.

2-(4'-Methoxy-[1,1'-biphenyl]-2-yl)-3-methylpyridine (6j)[1,4]

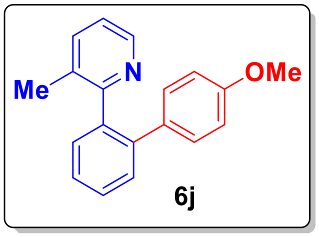

6j

White solid; ^{1}**H NMR** (400 MHz, $CDCl_3$) δ 8.50 (dd, J = 4.9, 1.6 Hz, 1H), 7.47 – 7.36 (m, 4H), 7.31 – 7.29 (m, 1H), 7.10 (dd, J = 7.7, 4.8 Hz, 1H), 7.05 – 7.02 (m, 2H), 6.72 – 6.68 (m, 2H), 3.74 (s, 3H), 1.74 (s, 3H). ^{13}**C NMR** (100 MHz, $CDCl_3$) δ 159.6, 158.3, 146.5, 140.2, 139.2, 137.4, 133.5, 131.6, 130.2, 129.8, 129.5, 128.3, 127.0, 122.0, 113.2, 55.1, 18.8. **HRMS (ESI)** Calcd for $[C_{19}H_{17}ON+K]^+$ 314.0928, Found 314.0942.

---

[10] N. R. Deprez and M. S. Sanford, *J. Am. Chem. Soc.*, **2009**, *131*, 11234-11241.

2-(2'-Methoxy-[1,1'-biphenyl]-2-yl)-3-methylpyridine (6k)

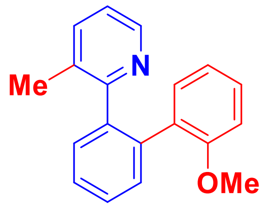

6k

Yellow oil; ^{1}**H NMR** (400 MHz, $CDCl_3$) δ 8.42 – 8.33 (d, 1H), 7.46 – 7.40 (m, 4H), 7.33 – 7.29 (m, 1H), 7.15 (ddd, *J* = 8.2, 7.4, 1.8 Hz, 1H), 7.02 (dt, *J* = 7.7, 5.3 Hz, 2H), 6.81 – 6.75 (m, 1H), 6.72 (d, *J* = 8.2 Hz, 1H), 3.53 (s, 3H), 1.92 (s, 3H). ^{13}**C NMR** (100 MHz, $CDCl_3$) δ 159.6, 156.2, 146.2, 140.3, 137.7, 137.4, 132.3, 131.7, 131.1, 130.0, 129.7, 128.5, 127.8, 127.3, 121.7, 120.1, 110.4, 55.0, 19.0. **HRMS** (ESI) Calcd for $[C_{19}H_{17}NO+Na]^+$ 298.1202, found 298.1192.

NMR Spectra

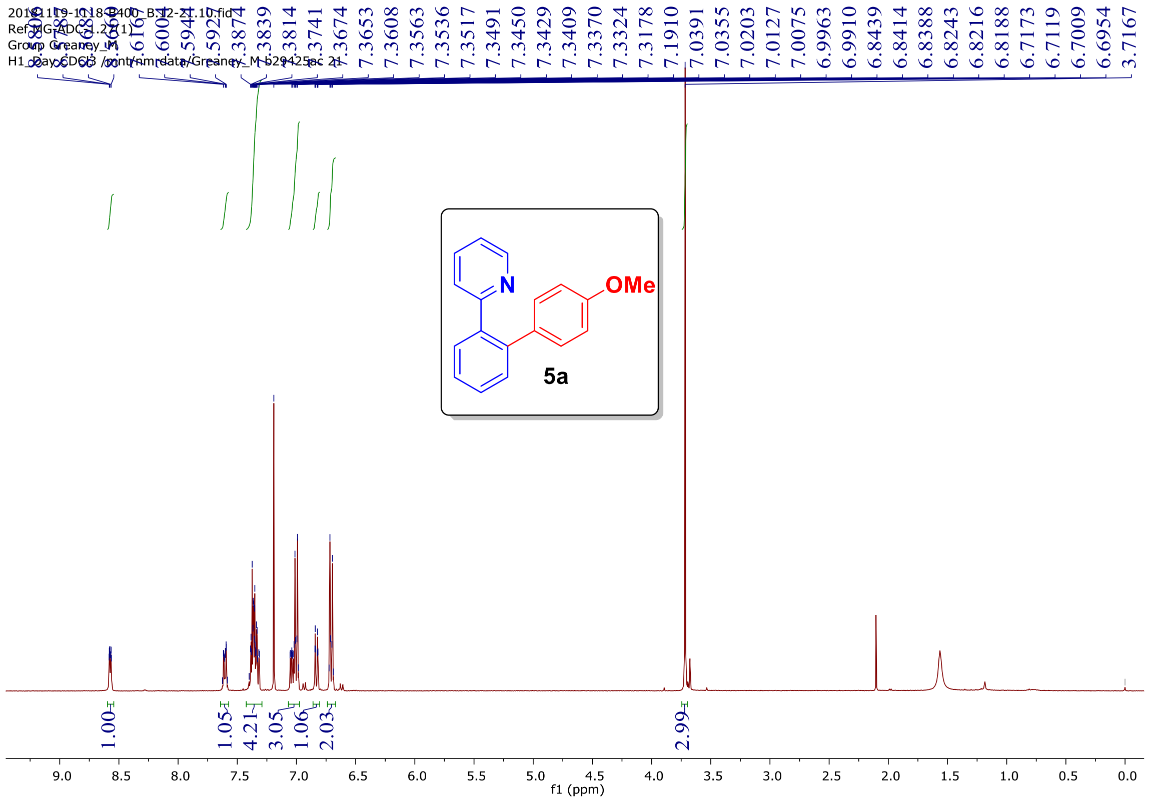

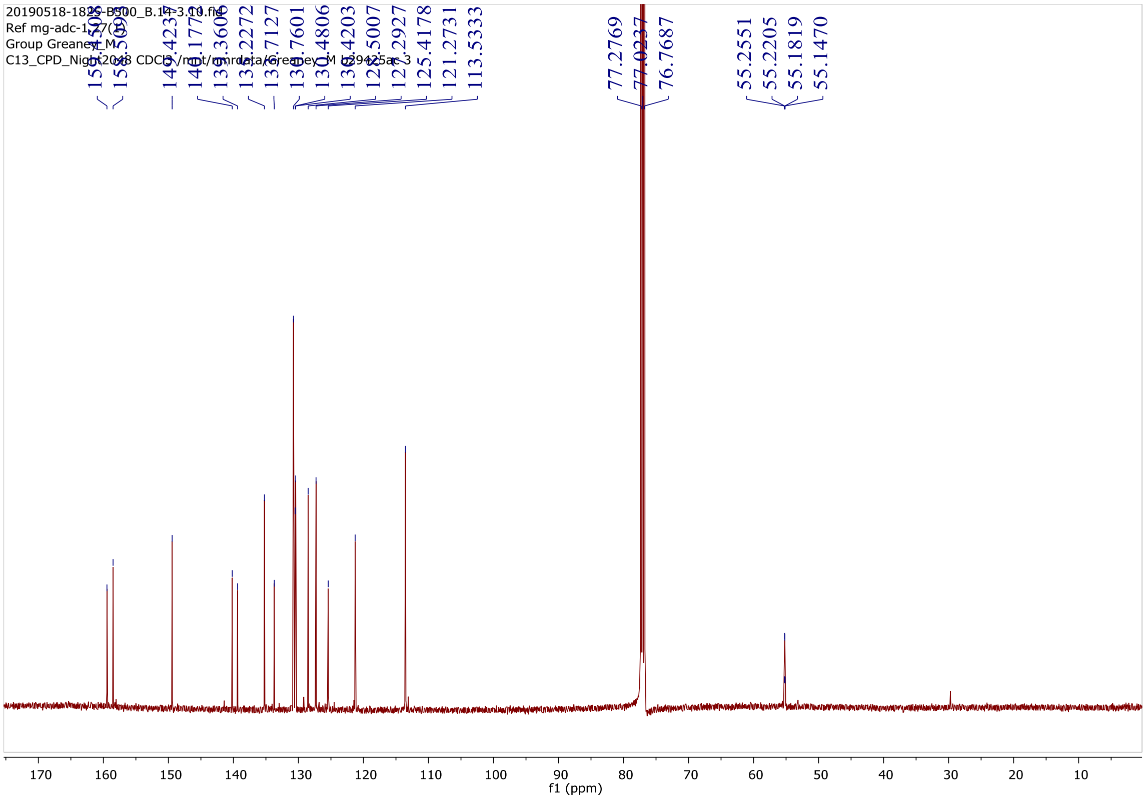

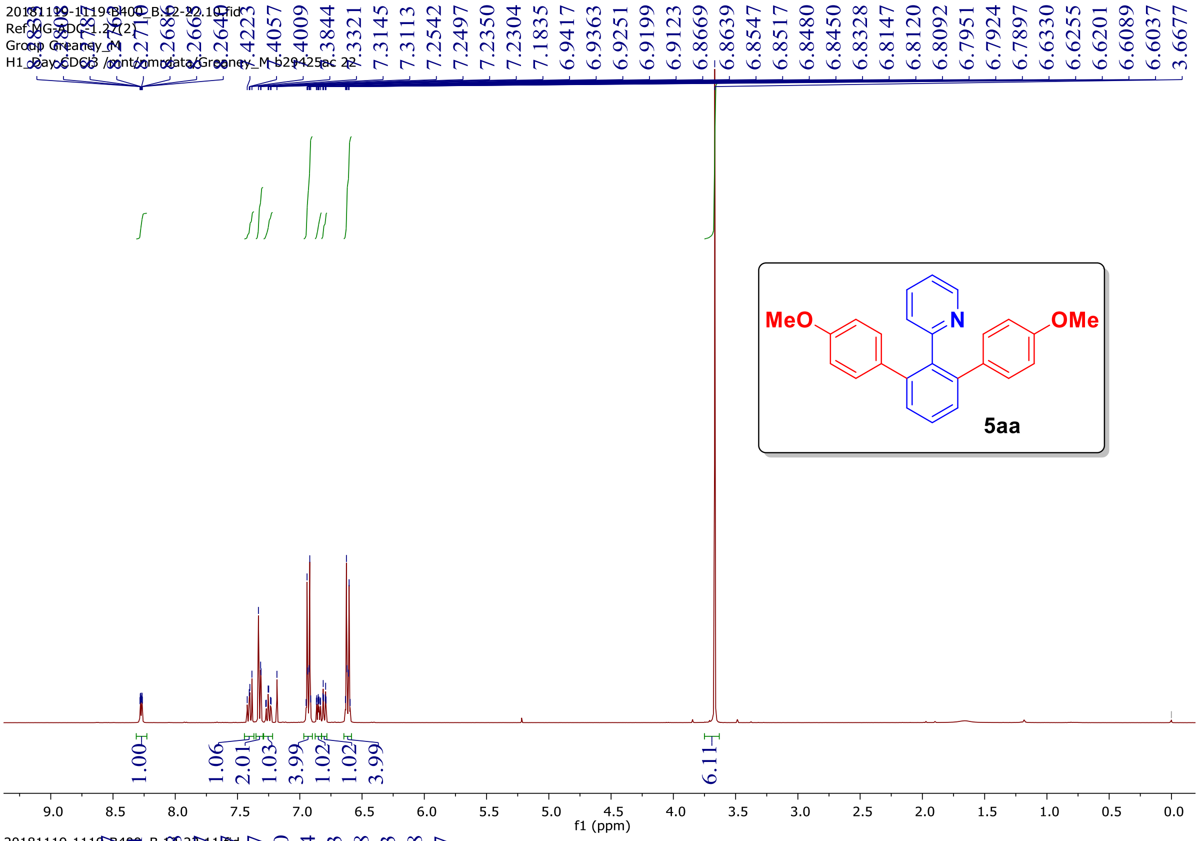
20181119-1119-B400_B.12-22.10.fid
Ref MG-ADC-1.27(2)
Group Greaney_M
H1_Day CDCl3 /mnt/nmrdata/Greaney_M b29425ac 22
7.4223
7.4057
7.4009
7.3844
7.3321
7.3145
7.3113
7.2542
7.2497
7.2350
7.2304
7.1835
6.9417
6.9363
6.9251
6.9199
6.9123
6.8669
6.8639
6.8547
6.8517
6.8480
6.8450
6.8328
6.8147
6.8120
6.8092
6.7951
6.7924
6.7897
6.6330
6.6255
6.6201
6.6089
6.6037
3.6677
MeO
N
OMe
5aa
1.00
1.06
2.01
1.03
3.99
1.02
1.02
3.99
6.11
f1 (ppm)

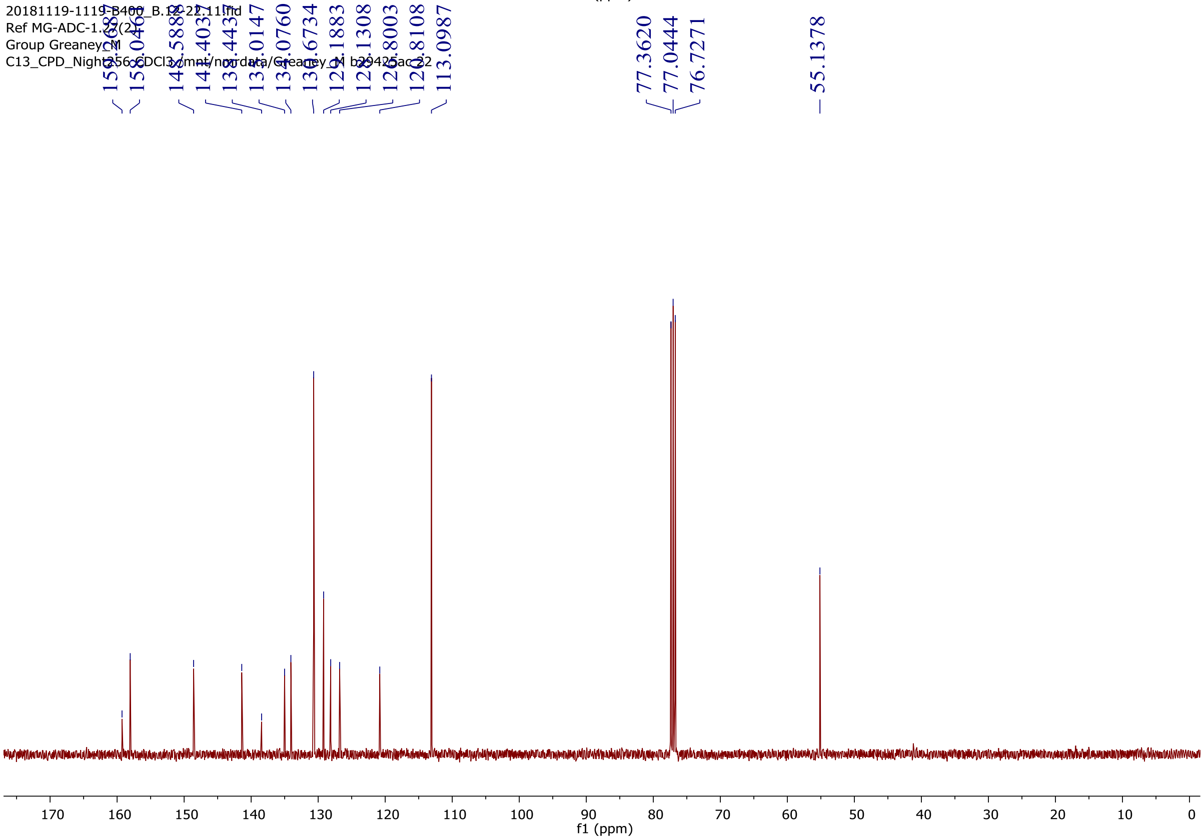
20181119-1119-B400_B.12-22.11.fid
Ref MG-ADC-1.27(2)
Group Greaney_M
C13_CPD_Night256 CDCl3 /mnt/nmrdata/Greaney_M b29425ac 22
77.3620
77.0444
76.7271
55.1378
f1 (ppm)

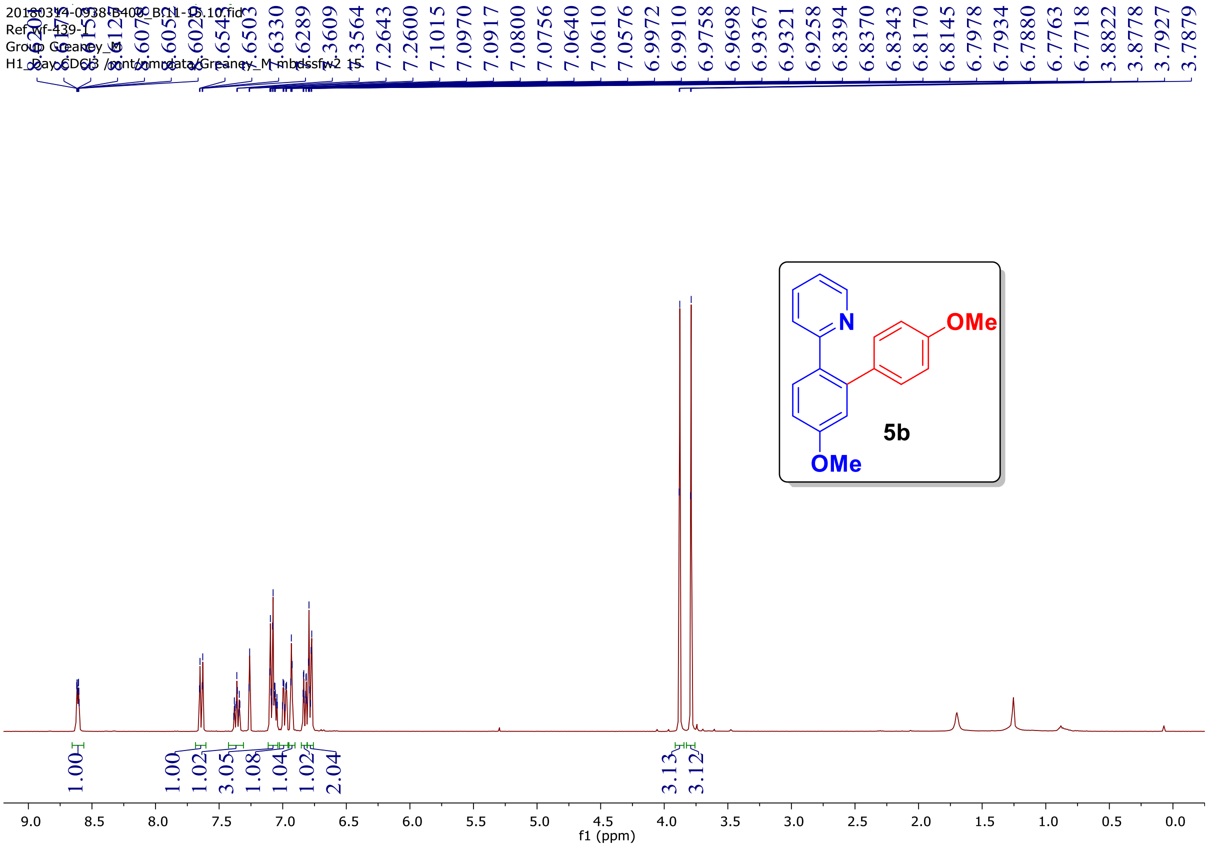

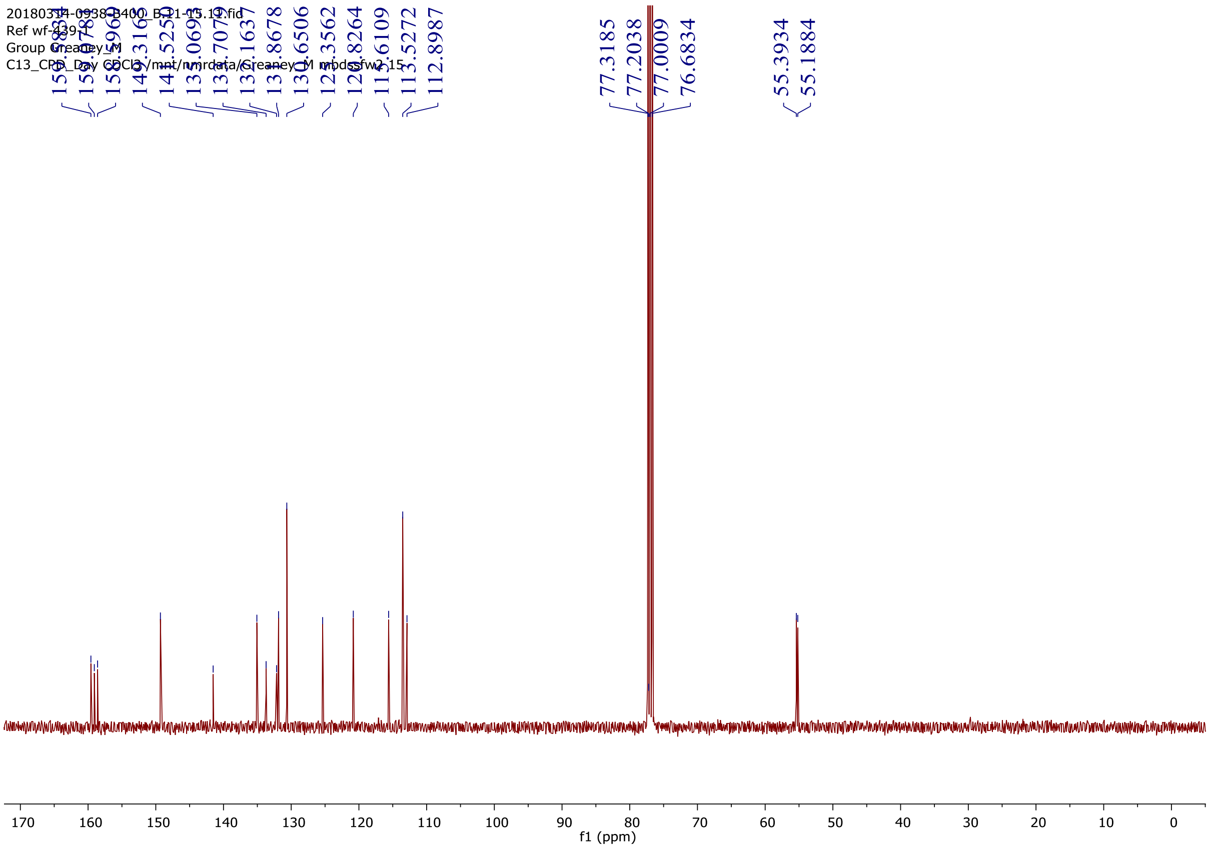

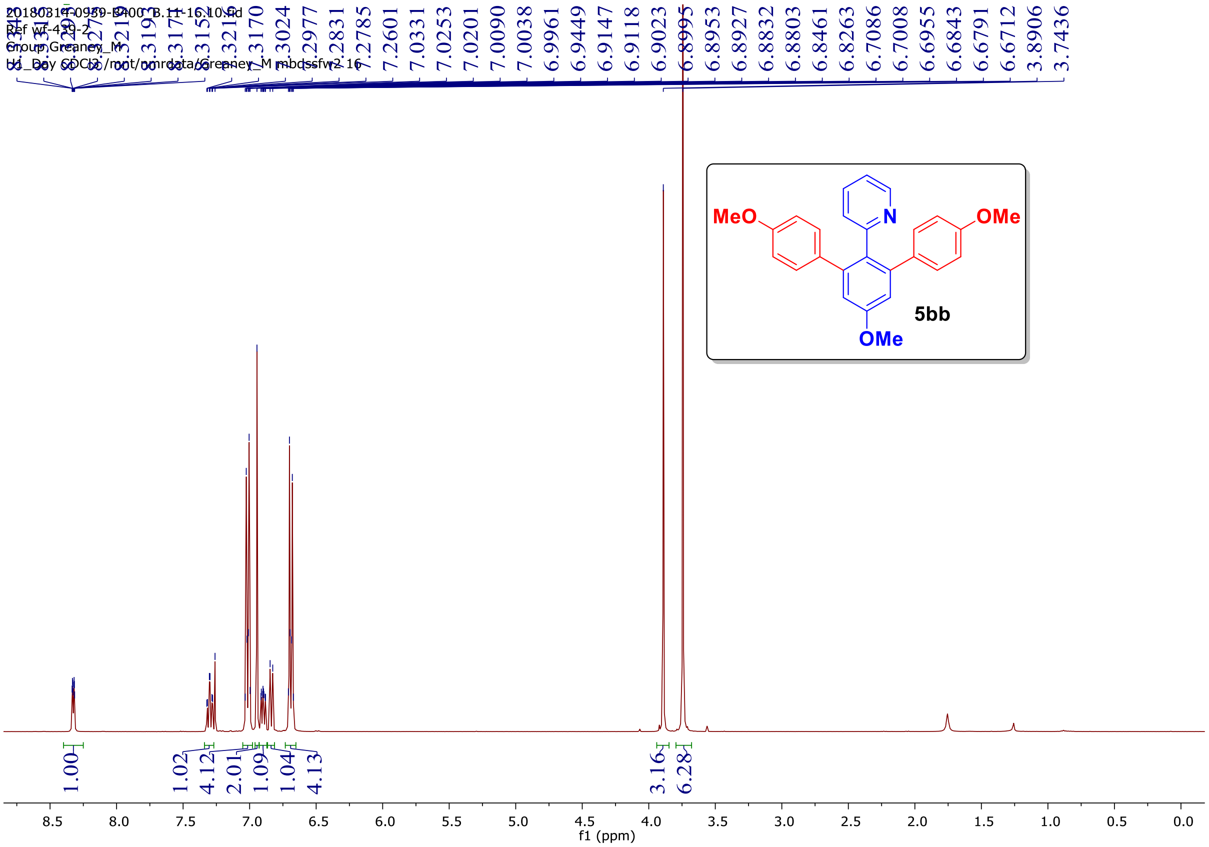
20180314-0939-B400_B.11-16.10.fid
Ref wf-439-2
Group Greaney_M
H1_Day CDCl3 /mnt/nmrdata/Greaney_M mbdssfw2 16
7.3024
7.2977
7.2831
7.2785
7.2601
7.0331
7.0253
7.0201
7.0090
7.0038
6.9961
6.9449
6.9147
6.9118
6.9023
6.8995
6.8953
6.8927
6.8832
6.8803
6.8461
6.8263
6.7086
6.7008
6.6955
6.6843
6.6791
6.6712
3.8906
3.7436
MeO
N
OMe
OMe
5bb
1.00
1.02
4.12
2.01
1.09
1.04
4.13
3.16
6.28
f1 (ppm)

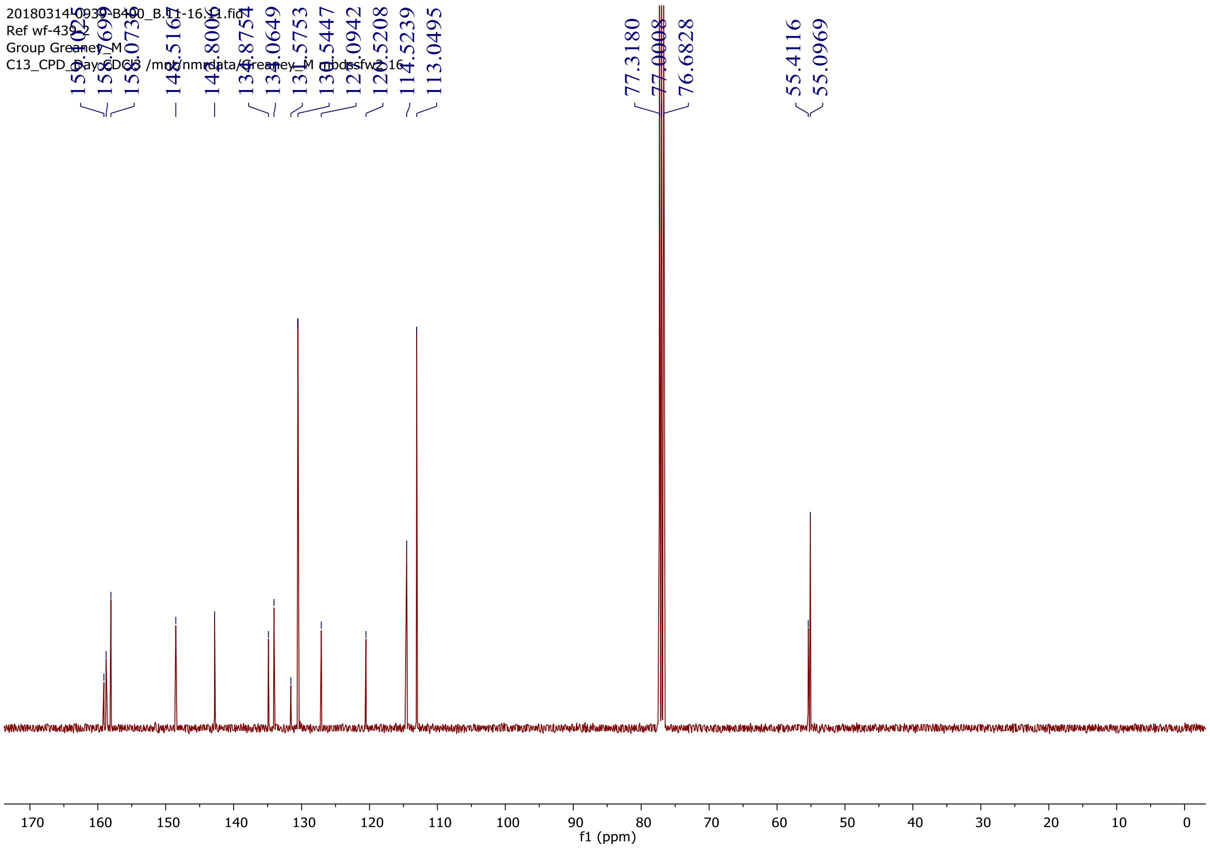
20180314-0939-B400_B.11-16.11.fid
Ref wf-439-2
Group Greaney_M
C13_CPD_Day CDCl3 /mnt/nmrdata/Greaney_M mbdssfw2 16
134.8754
134.0649
130.5447
127.0942
120.5208
114.5239
113.0495
77.3180
77.0008
76.6828
55.4116
55.0969
f1 (ppm)

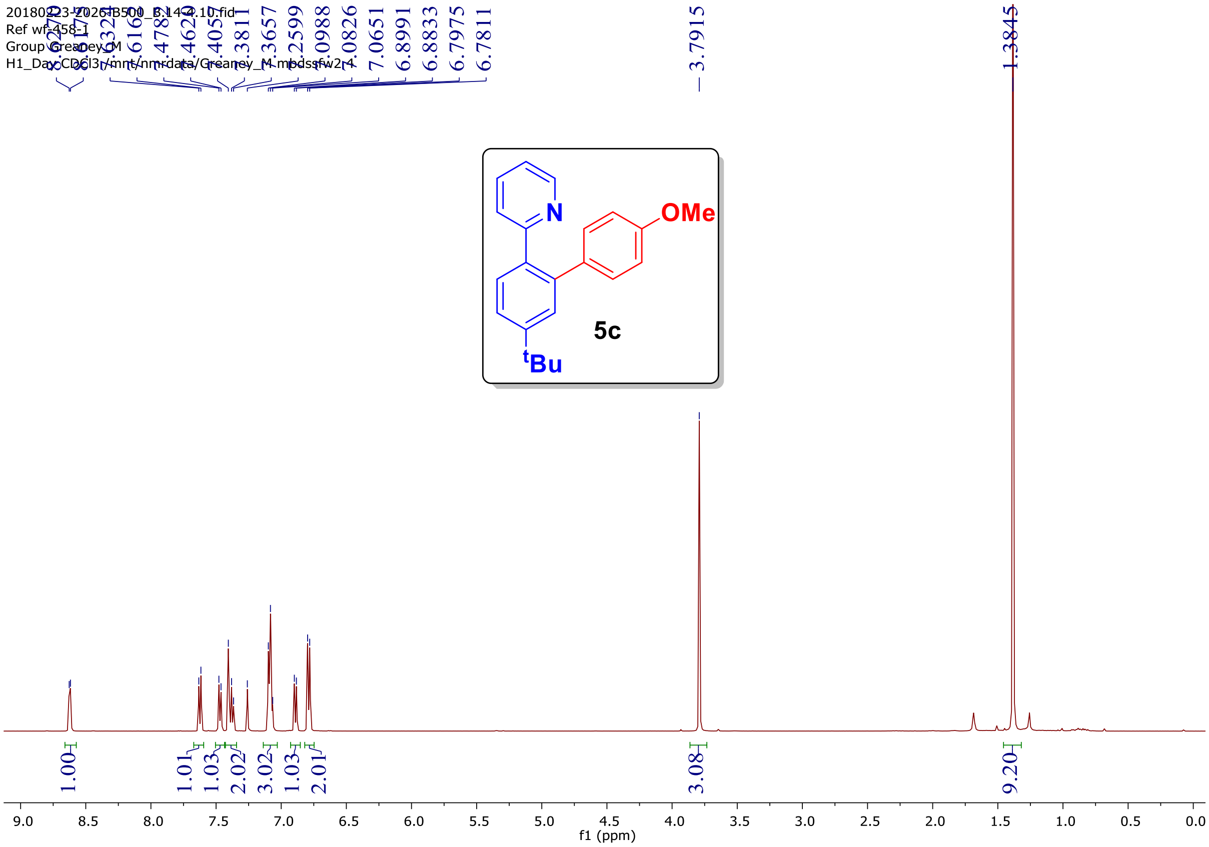
20180223-2026-B500_B.14-4.10.fid
Ref wf-458-1
Group Greaney_M
H1_Day CDCl3 /mnt/nmrdata/Greaney_M mbdssfw2 4
7.3811
7.3657
7.2599
7.0988
7.0826
7.0651
6.8991
6.8833
6.7975
6.7811
3.7915
1.3845
N
OMe
tBu
5c
1.00
1.01
1.03
2.02
3.02
1.03
2.01
3.08
9.20
f1 (ppm)

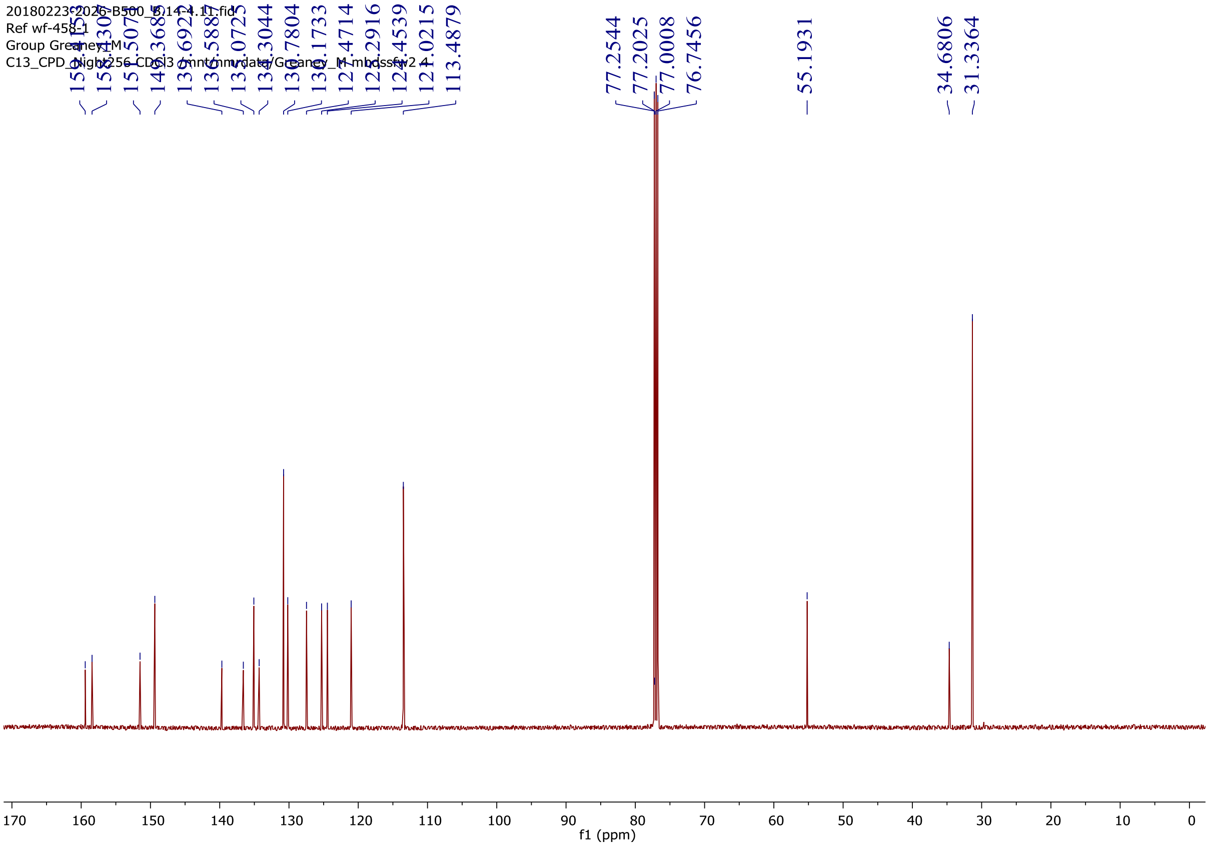
20180223-2026-B500_B.14-4.11.fid
Ref wf-458-1
Group Greaney_M
C13_CPD_Night256 CDCl3 /mnt/nmrdata/Greaney_M mbdssfw2 4
134.3044
130.7804
130.1733
127.4714
125.2916
124.4539
121.0215
113.4879
77.2544
77.2025
77.0008
76.7456
55.1931
34.6806
31.3364
f1 (ppm)

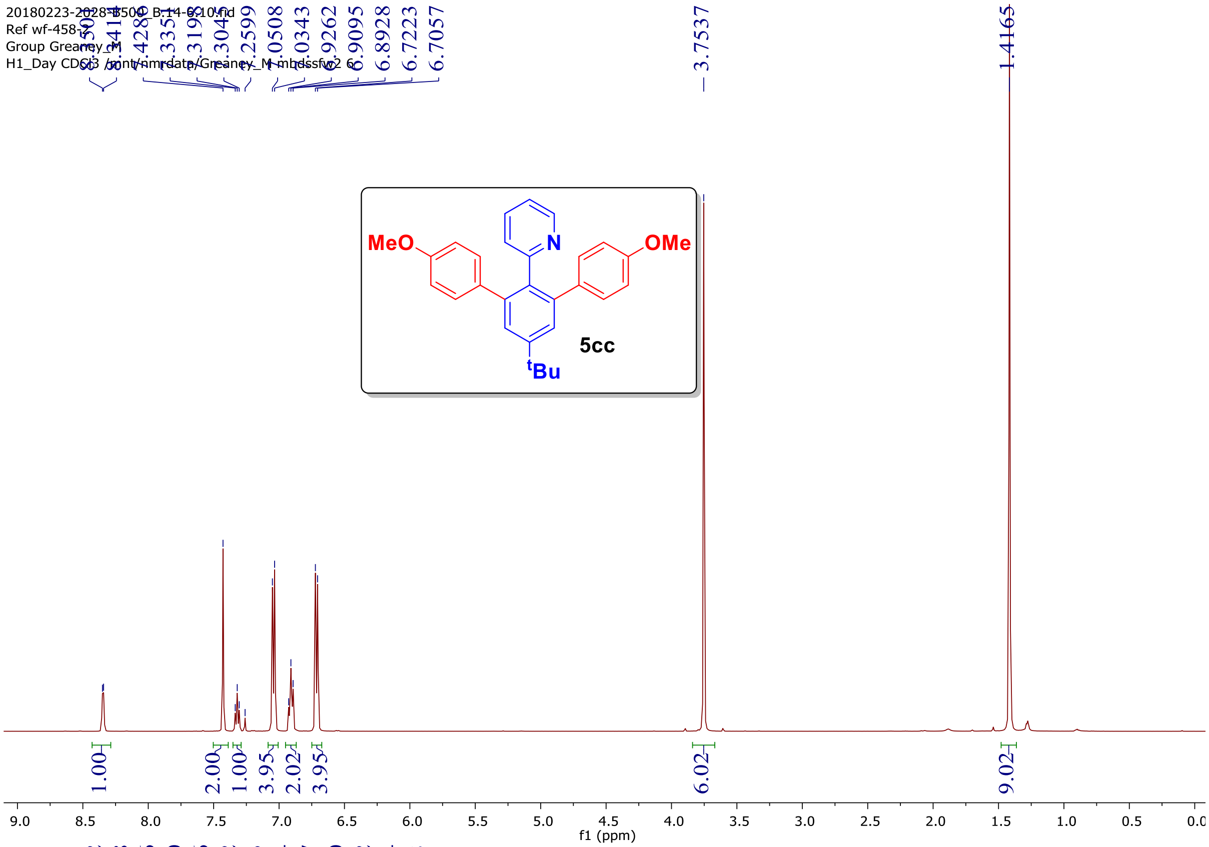
Ref wf-458-2
Group Greaney_M
3.7537
1.4165
6.8928
6.7223
6.7057
MeO
OMe
N
5cc
tBu
1.00
2.00
1.00
3.95
2.02
3.95
6.02
9.02
f1 (ppm)

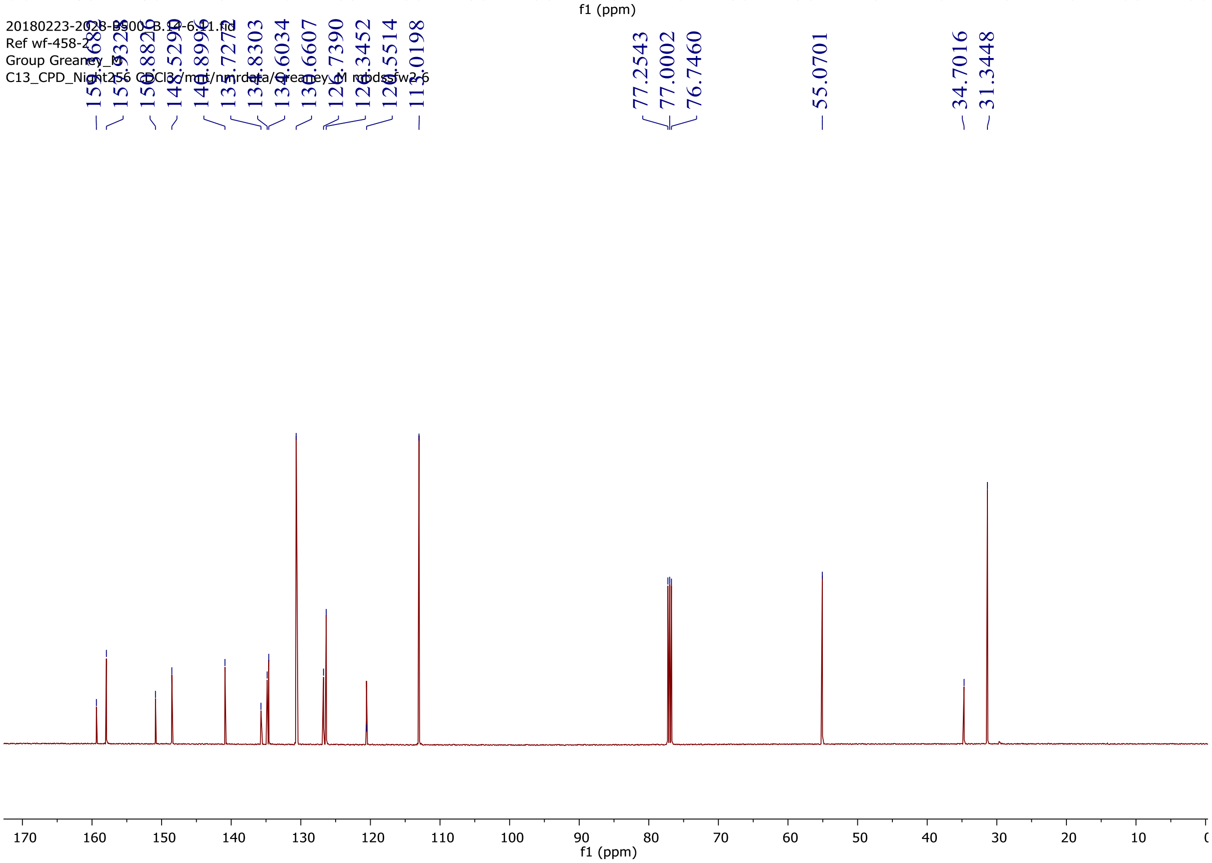
Ref wf-458-2
Group Greaney_M
77.2543
77.0002
76.7460
55.0701
34.7016
31.3448
f1 (ppm)

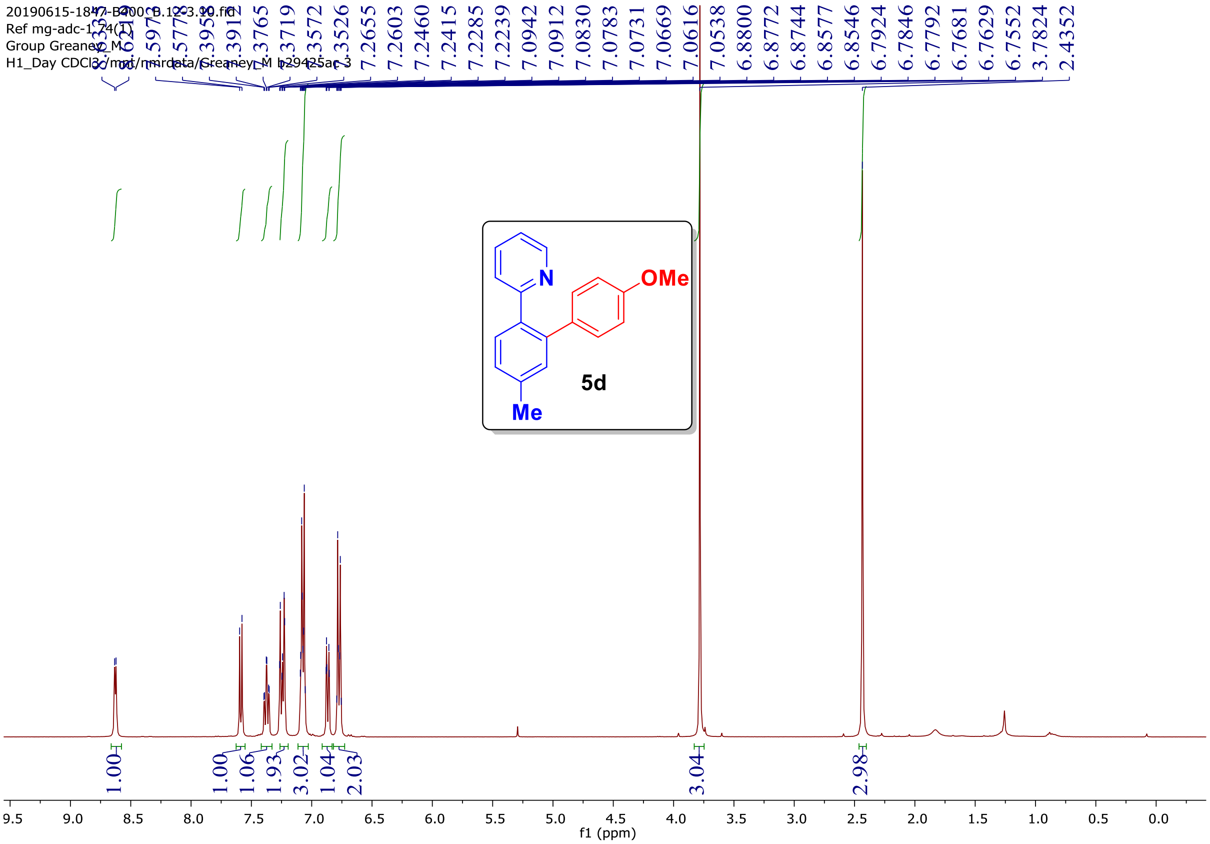
Ref mg-adc-1_74(1)
Group Greaney_M
7.3765
7.3719
7.3572
7.3526
7.2655
7.2603
7.2460
7.2415
7.2285
7.2239
7.0942
7.0912
7.0830
7.0783
7.0731
7.0669
7.0616
7.0538
6.8800
6.8772
6.8744
6.8577
6.8546
6.7924
6.7846
6.7792
6.7681
6.7629
6.7552
3.7824
2.4352
N
OMe
5d
Me
1.00
1.00
1.06
1.93
3.02
1.04
2.03
3.04
2.98
9.5
9.0
8.5
8.0
7.5
7.0
6.5
6.0
5.5
5.0
4.5
4.0
3.5
3.0
2.5
2.0
1.5
1.0
0.5
0.0
f1 (ppm)

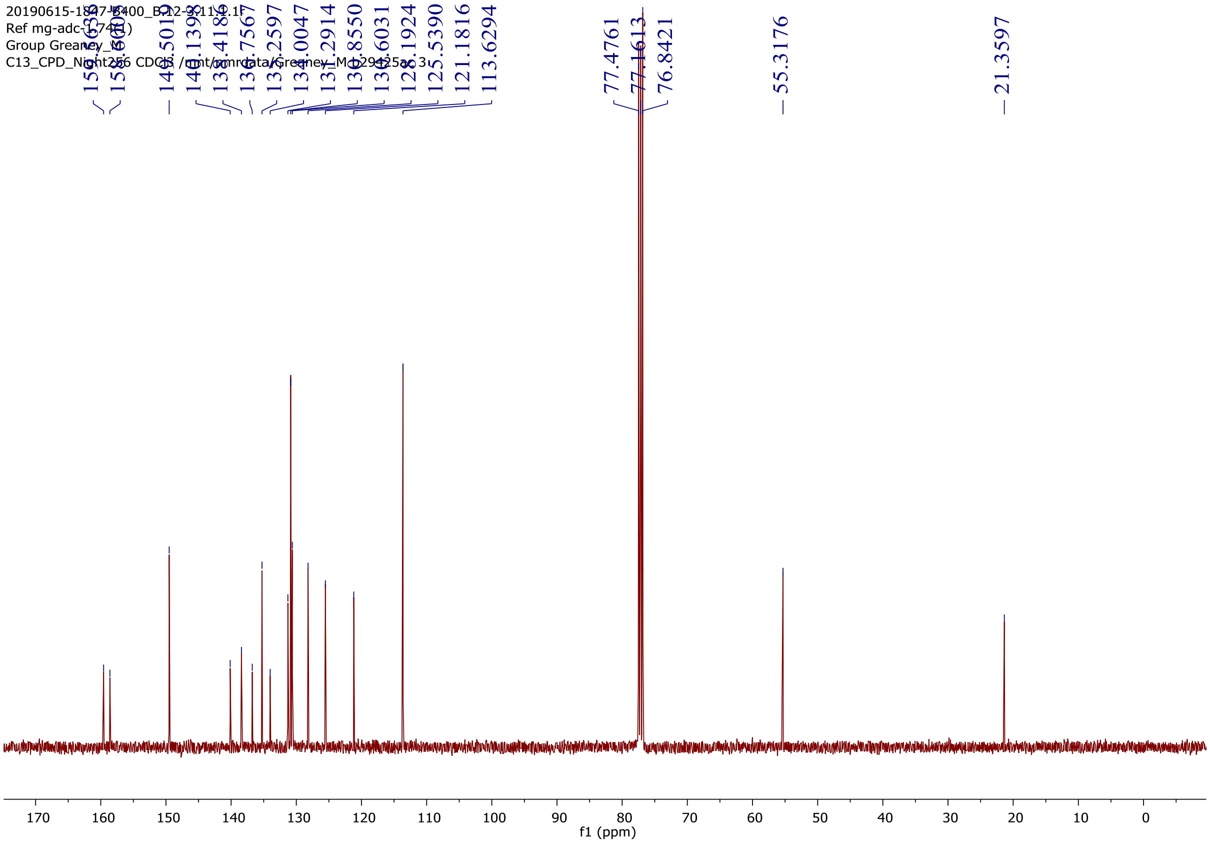
Group Greaney_M
77.4761
77.1613
76.8421
55.3176
21.3597
113.6294
121.1816
125.5390
170
160
150
140
130
120
110
100
90
80
70
60
50
40
30
20
10
0
f1 (ppm)

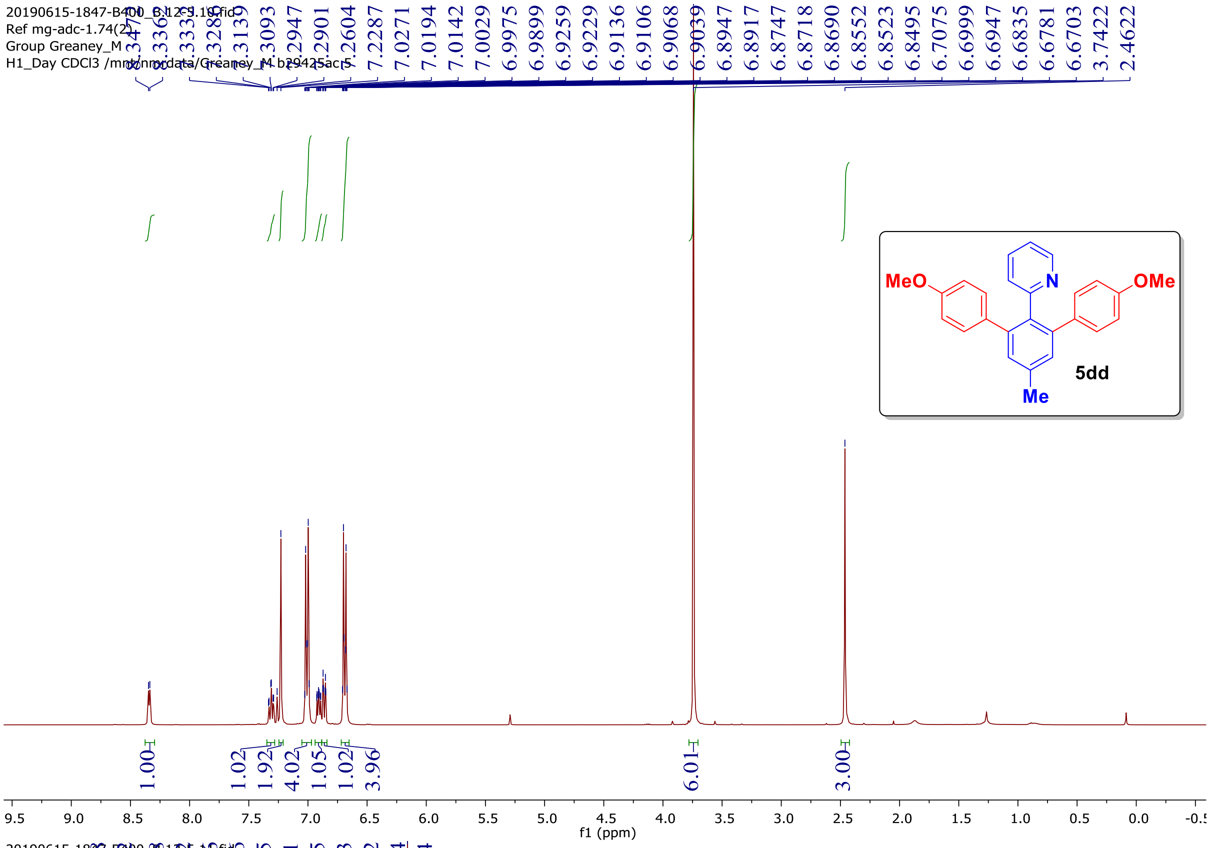
20190615-1847-B400_B.12-5.10.fid
Ref mg-adc-1.74(2)
Group Greaney_M
H1_Day CDCl3 /mnt/nmrdata/Greaney_M.b29425ac 5
7.3093
7.2947
7.2901
7.2604
7.2287
7.0271
7.0194
7.0142
7.0029
6.9975
6.9899
6.9259
6.9229
6.9136
6.9106
6.9068
6.9039
6.8947
6.8917
6.8747
6.8718
6.8690
6.8552
6.8523
6.8495
6.7075
6.6999
6.6947
6.6835
6.6781
6.6703
3.7422
2.4622
MeO
N
OMe
5dd
Me
1.00
1.02
1.92
4.02
1.05
1.02
3.96
6.01
3.00
f1 (ppm)

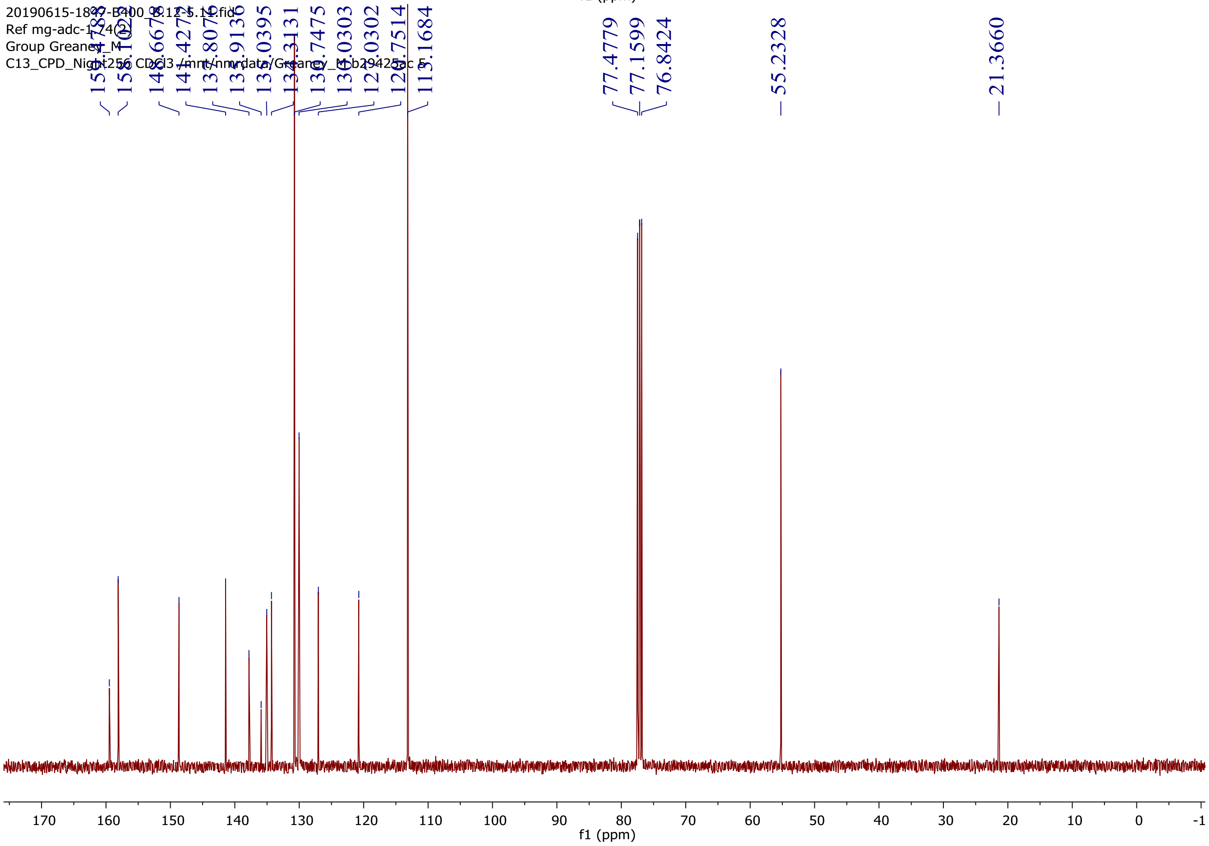
20190615-1847-B400_B.12-5.11.fid
Ref mg-adc-1.74(2)
Group Greaney_M
C13_CPD_Night256 CDCl3 /mnt/nmrdata/Greaney_M.b29425ac 5
77.4779
77.1599
76.8424
55.2328
21.3660
f1 (ppm)

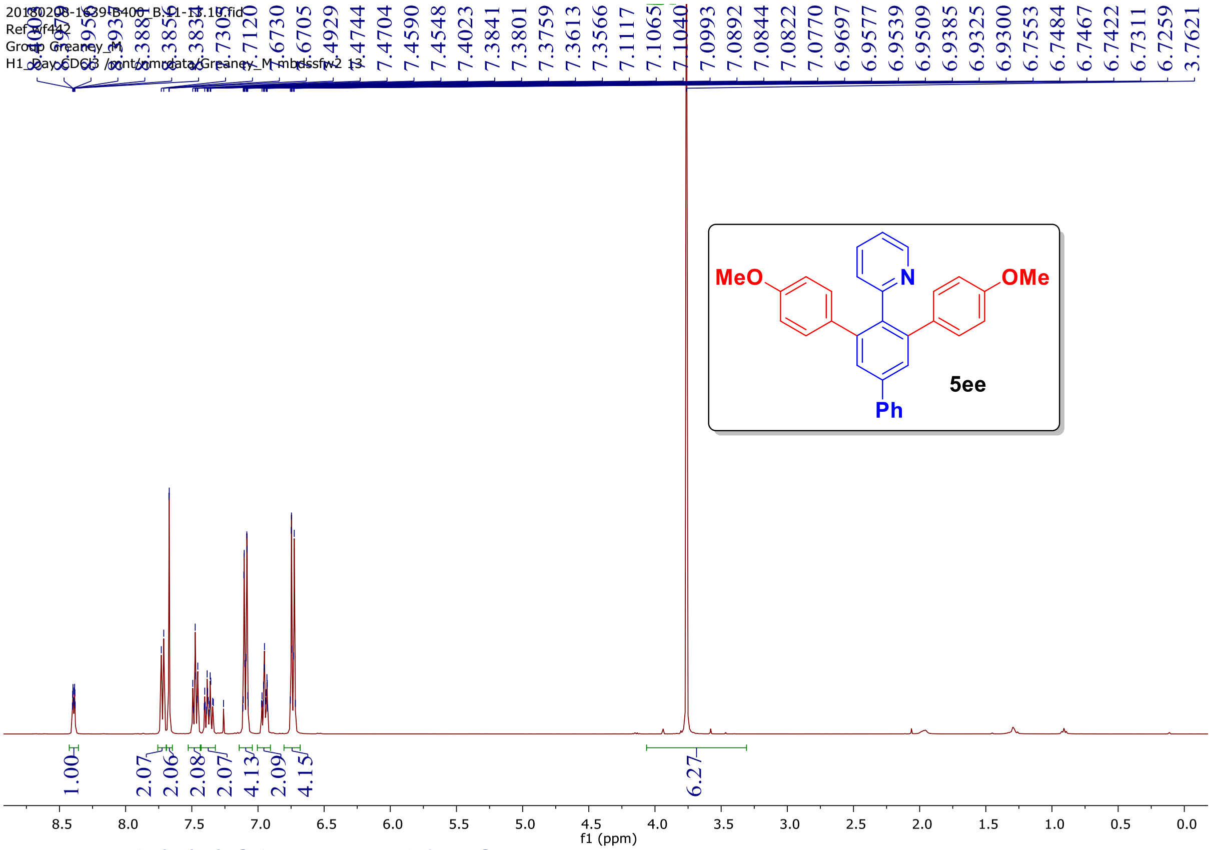

Ref wf442
Group Greaney_M
7.7120
7.6730
7.6705
7.4929
7.4744
7.4704
7.4590
7.4548
7.4023
7.3841
7.3801
7.3759
7.3613
7.3566
7.1117
7.1065
7.1040
7.0993
7.0892
7.0844
7.0822
7.0770
6.9697
6.9577
6.9539
6.9509
6.9385
6.9325
6.9300
6.7553
6.7484
6.7467
6.7422
6.7311
6.7259
3.7621
MeO
N
OMe
5ee
Ph
1.00
2.07
2.06
2.08
2.07
4.13
2.09
4.15
6.27
f1 (ppm)

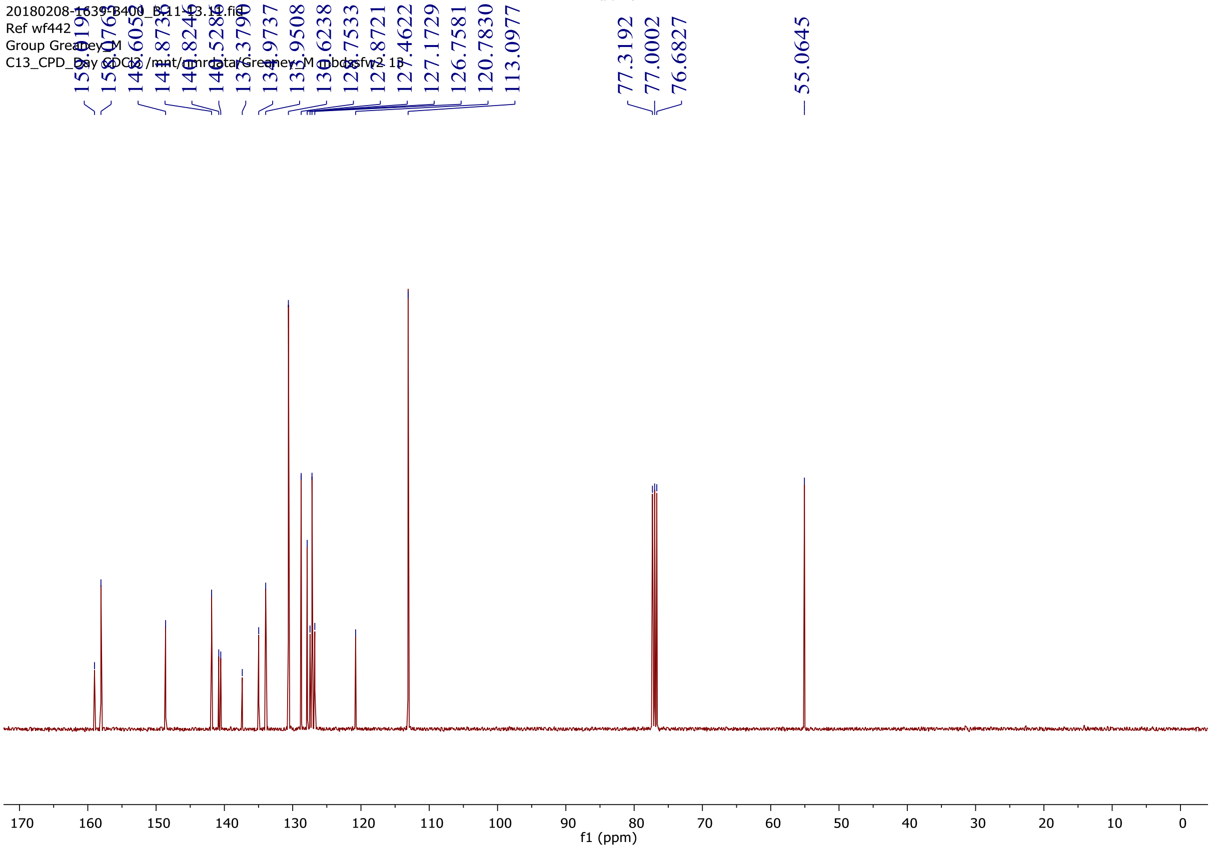

Ref wf442
133.9737
133.9508
130.6238
128.7533
127.8721
127.4622
127.1729
126.7581
120.7830
113.0977
77.3192
77.0002
76.6827
55.0645
f1 (ppm)

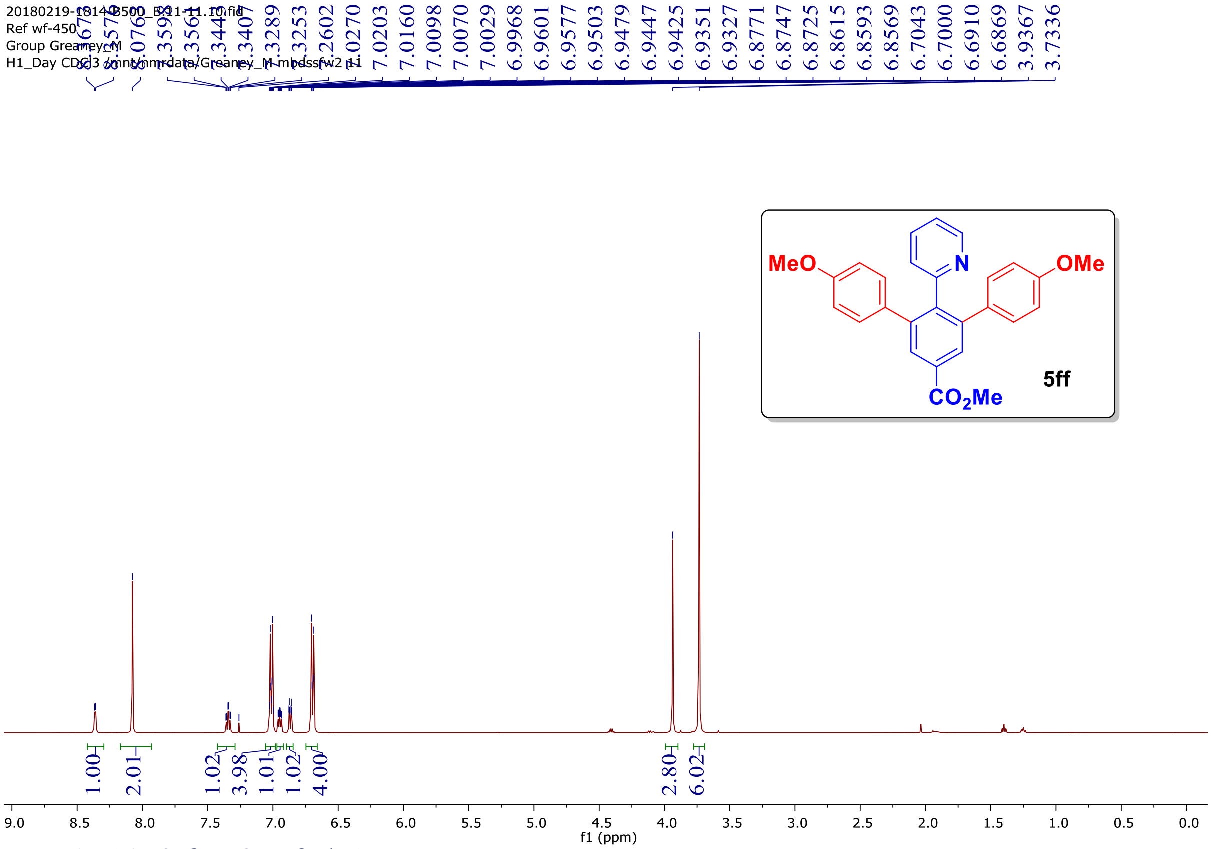

20180219-1814-B500_B11-11.10.fid
Ref wf-450
Group Greaney_M
H1_Day CDCl3 /mnt/nmrdata/Greaney_M mbdssfw2 11
7.3289
7.3253
7.2602
7.0270
7.0203
7.0160
7.0098
7.0070
7.0029
6.9968
6.9601
6.9577
6.9503
6.9479
6.9447
6.9425
6.9351
6.9327
6.8771
6.8747
6.8725
6.8615
6.8593
6.8569
6.7043
6.7000
6.6910
6.6869
3.9367
3.7336
MeO
N
OMe
CO_2Me
5ff
1.00
2.01
1.02
3.98
1.01
1.02
4.00
2.80
6.02
f1 (ppm)

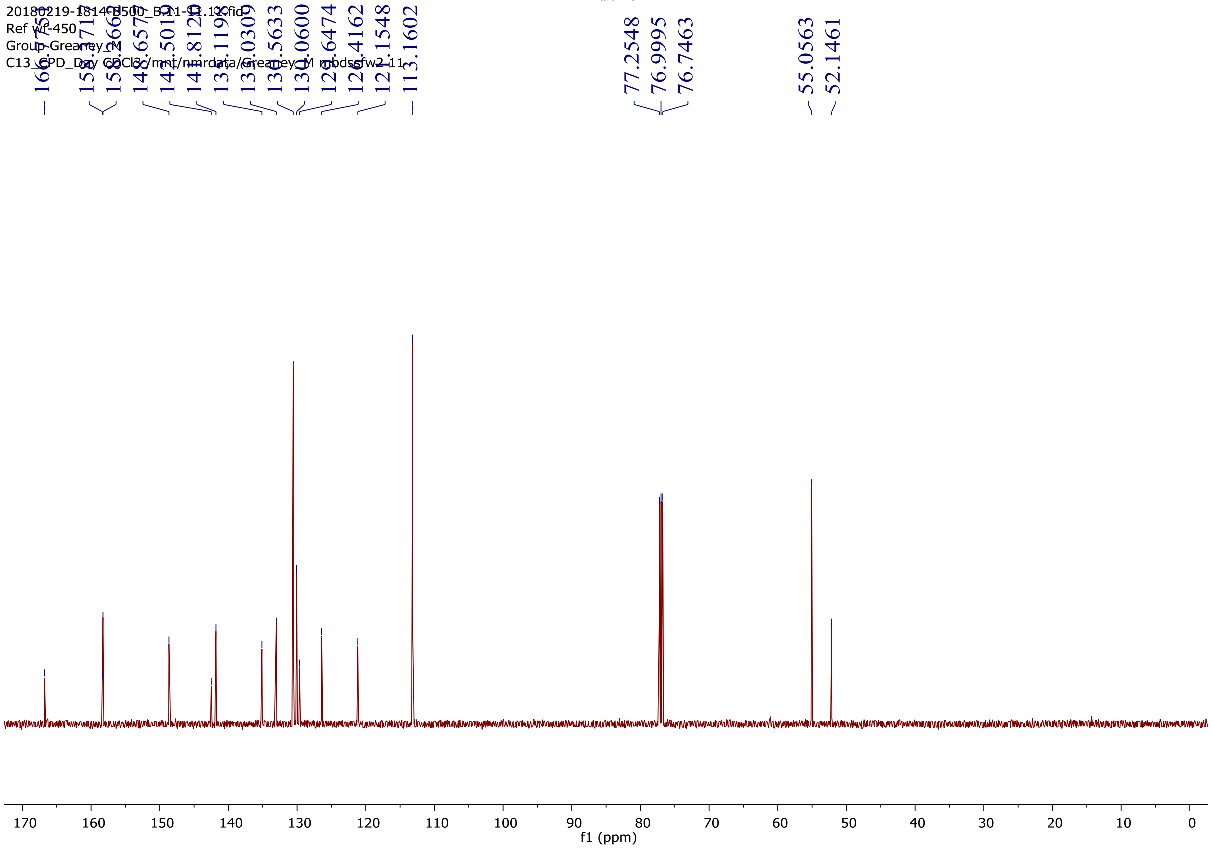

20180219-1814-B500_B11-11.11.fid
Ref wf-450
Group Greaney_M
C13_CPD_Day CDCl3 /mnt/nmrdata/Greaney_M mbdssfw2-11
130.0600
129.6474
121.4162
121.1548
113.1602
77.2548
76.9995
76.7463
55.0563
52.1461
f1 (ppm)

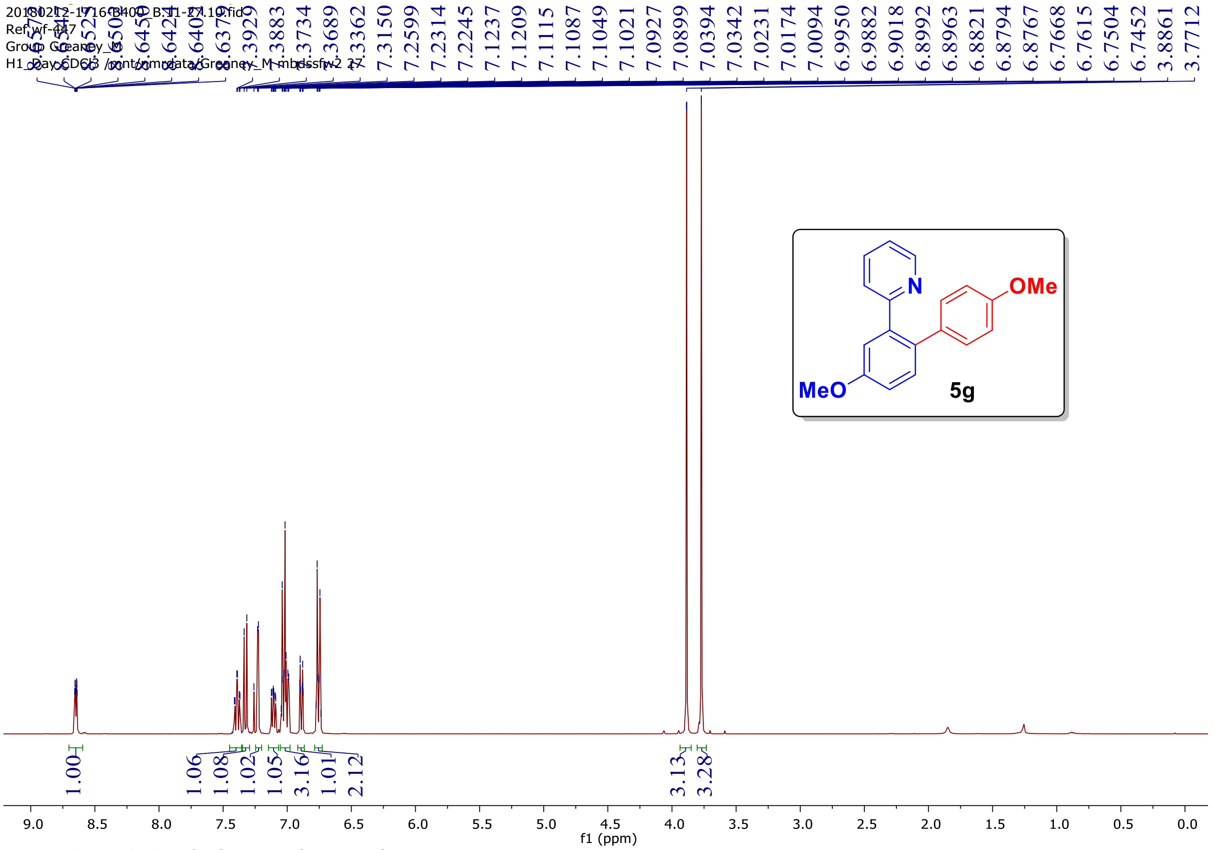
20180212-1716-B400_B.11-27.10.fid
Ref wf-447
Group Greaney_M
H1_Day CDCl3 /mnt/nmrdata/Greaney_M mbdssfw2 27
7.3929
7.3883
7.3734
7.3689
7.3362
7.3150
7.2599
7.2314
7.2245
7.1237
7.1209
7.1115
7.1087
7.1049
7.1021
7.0927
7.0899
7.0394
7.0342
7.0231
7.0174
7.0094
6.9950
6.9882
6.9018
6.8992
6.8963
6.8821
6.8794
6.8767
6.7668
6.7615
6.7504
6.7452
3.8861
3.7712
MeO
N
OMe
5g
1.00
1.06
1.08
1.02
1.05
3.16
1.01
2.12
3.13
3.28
f1 (ppm)

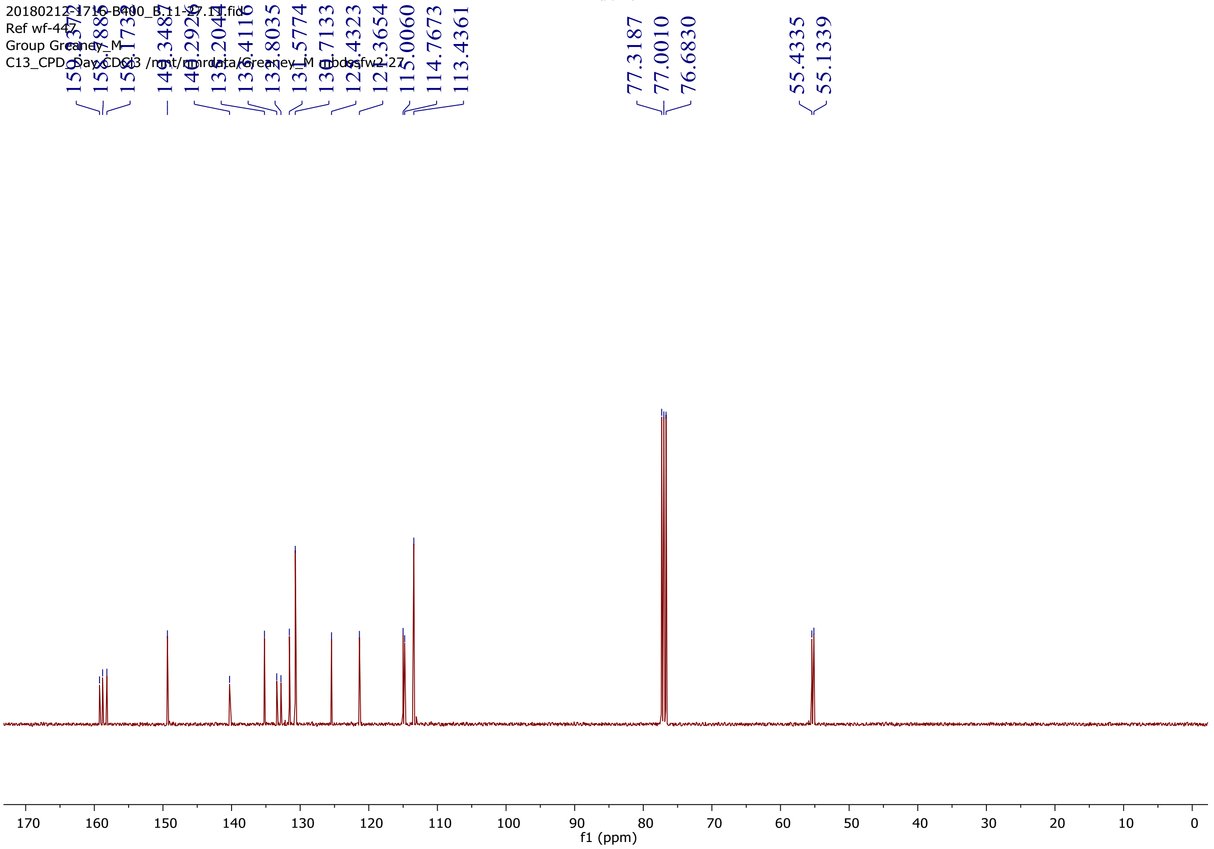
20180212-1716-B400_B.11-27.11.fid
Ref wf-447
Group Greaney_M
C13_CPD_Day CDCl3 /mnt/nmrdata/Greaney_M mbdssfw2 27
77.3187
77.0010
76.6830
55.4335
55.1339
115.0060
114.7673
113.4361
f1 (ppm)

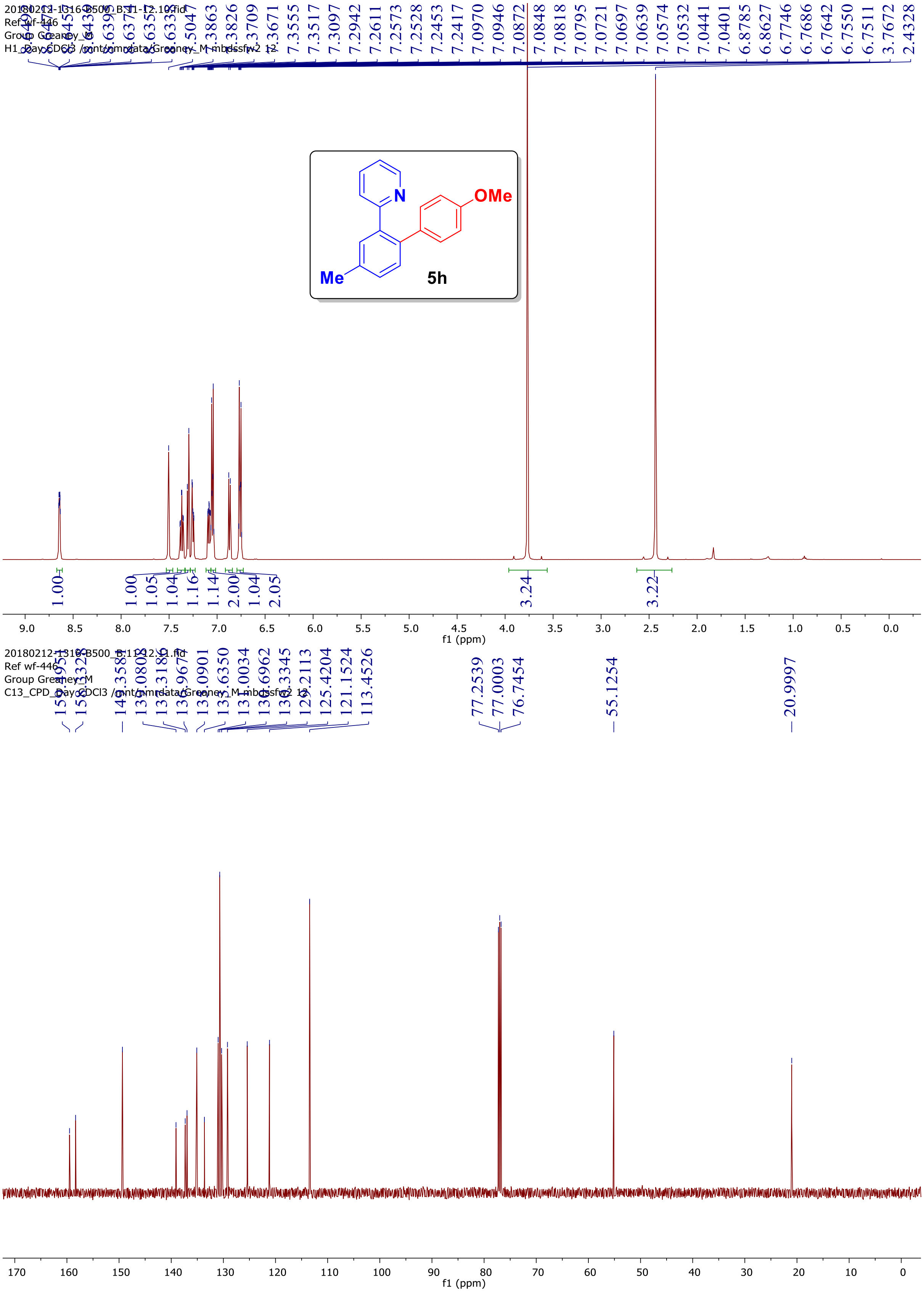
20180212-1316-B500_B.11-12.10.fid
Ref wf-446
Group Greaney_M
H1_Day CDCl3 /mnt/nmrdata/Greaney_M mbdssfw2 12
N
OMe
Me
5h
f1 (ppm)
20180212-1316-B500_B.11-12.11.fid
Ref wf-446
Group Greaney_M
C13_CPD_Day CDCl3 /mnt/nmrdata/Greaney_M mbdssfw2 12
77.2539
77.0003
76.7454
55.1254
20.9997
f1 (ppm)

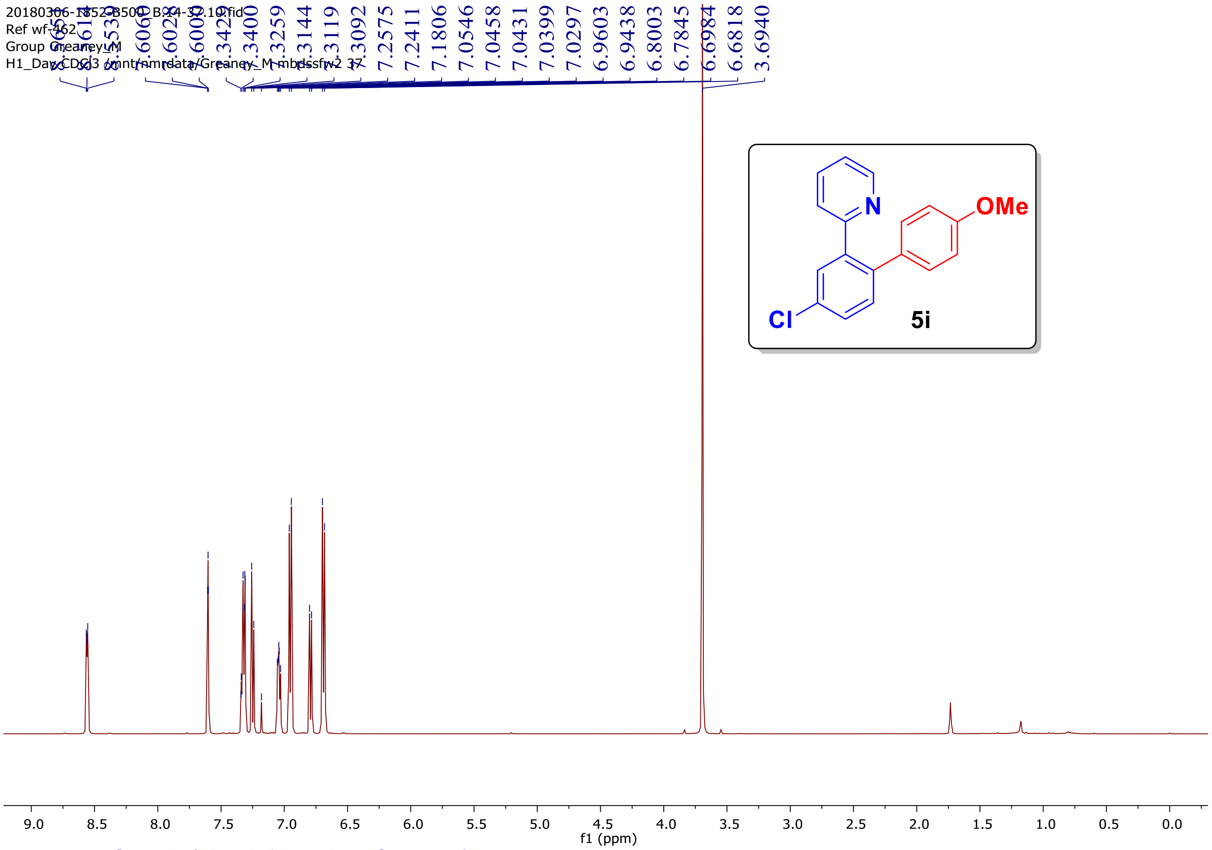
20180306-1852-B500_B 14-37.10.fid
Ref wf-462
Group Greaney_M
H1_Day CDCl3 /mnt/nmrdata/Greaney_M mbdssfw2 37
7.2575
7.2411
7.1806
7.0546
7.0458
7.0431
7.0399
7.0297
6.9603
6.9438
6.8003
6.7845
6.6984
6.6818
3.6940
OMe
N
Cl
5i
f1 (ppm)

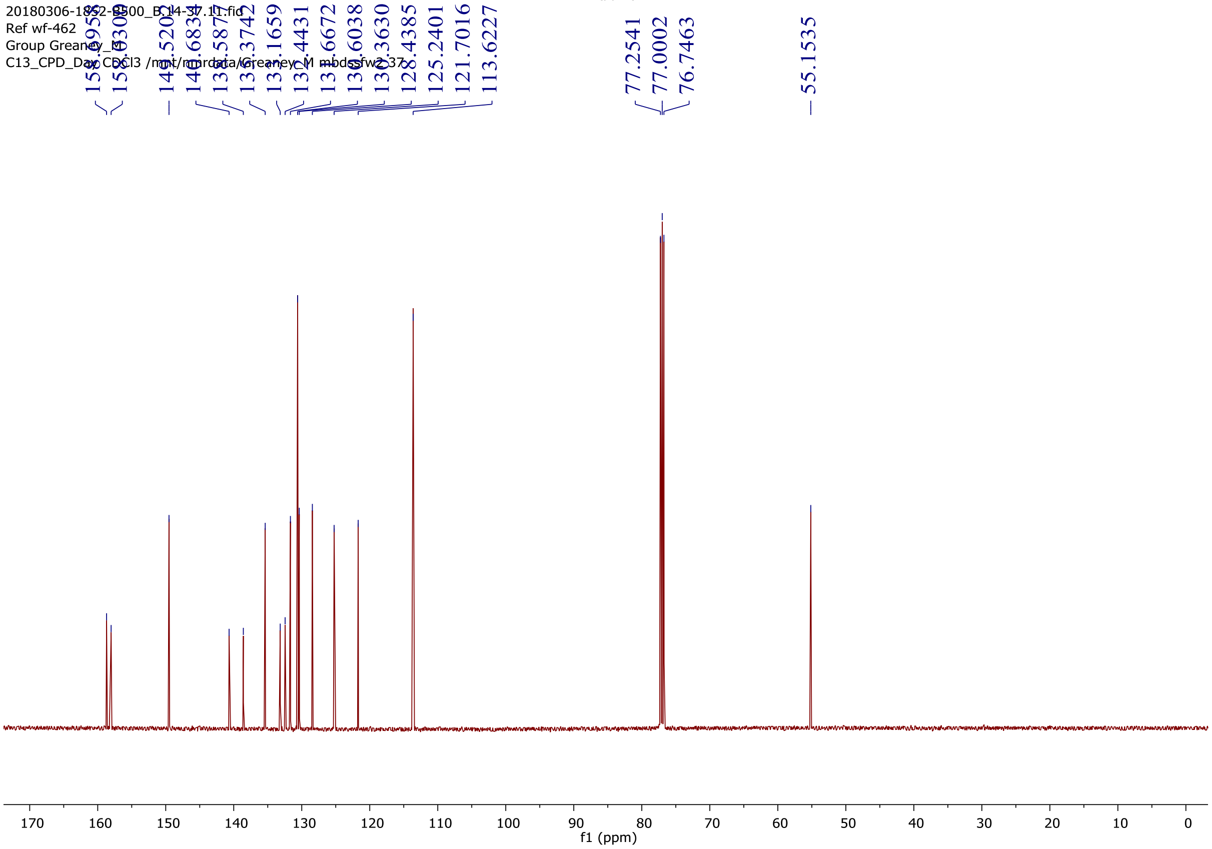
20180306-1852-B500_B 14-37.11.fid
Ref wf-462
Group Greaney_M
C13_CPD_Day CDCl3 /mnt/nmrdata/Greaney_M mbdssfw2 37
128.4385
125.2401
121.7016
113.6227
77.2541
77.0002
76.7463
55.1535
f1 (ppm)

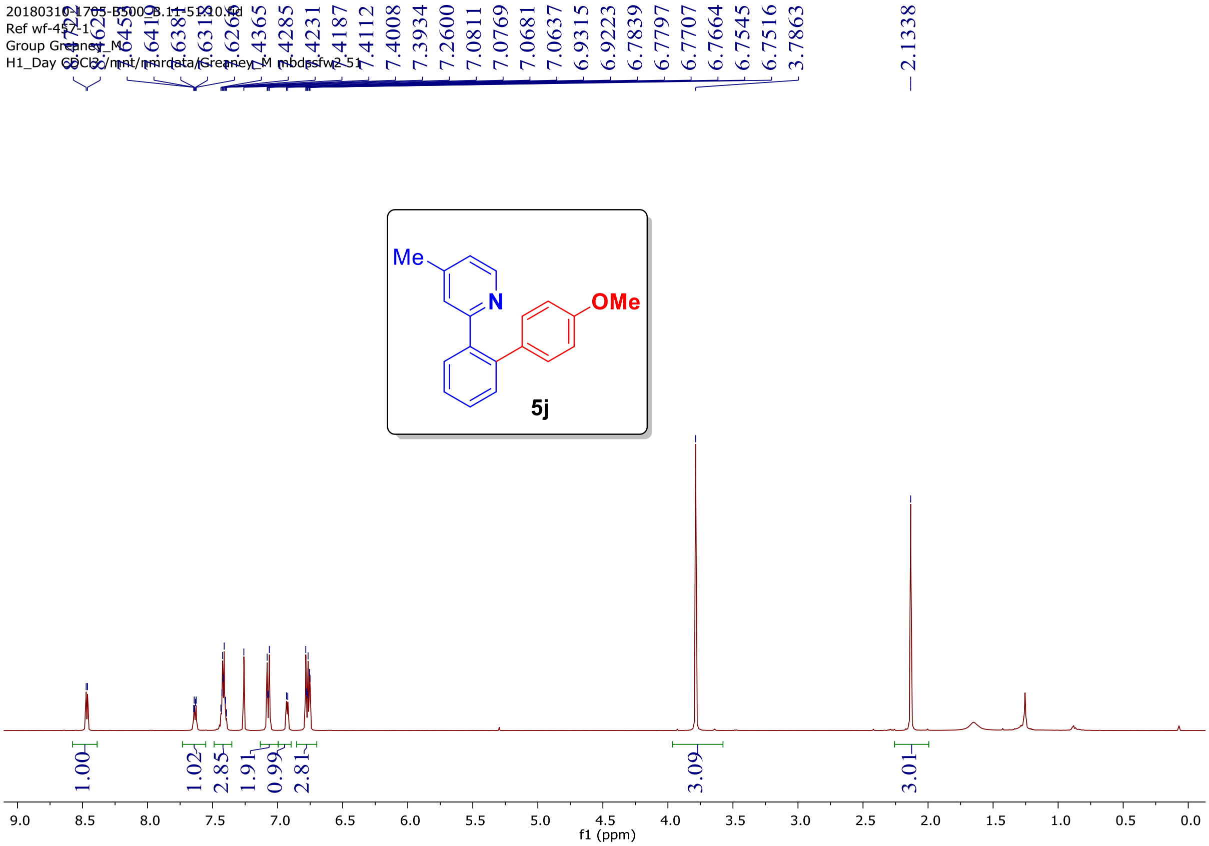
Ref wf-457-1
Group Greaney_M
7.6459
7.4365
7.4285
7.4231
7.4187
7.4112
7.4008
7.3934
7.2600
7.0811
7.0769
7.0681
7.0637
6.9315
6.9223
6.7839
6.7797
6.7707
6.7664
6.7545
6.7516
3.7863
2.1338
Me
N
OMe
5j
1.00
1.02
2.85
1.91
0.99
2.81
3.09
3.01
9.0 8.5 8.0 7.5 7.0 6.5 6.0 5.5 5.0 4.5 4.0 3.5 3.0 2.5 2.0 1.5 1.0 0.5 0.0
f1 (ppm)

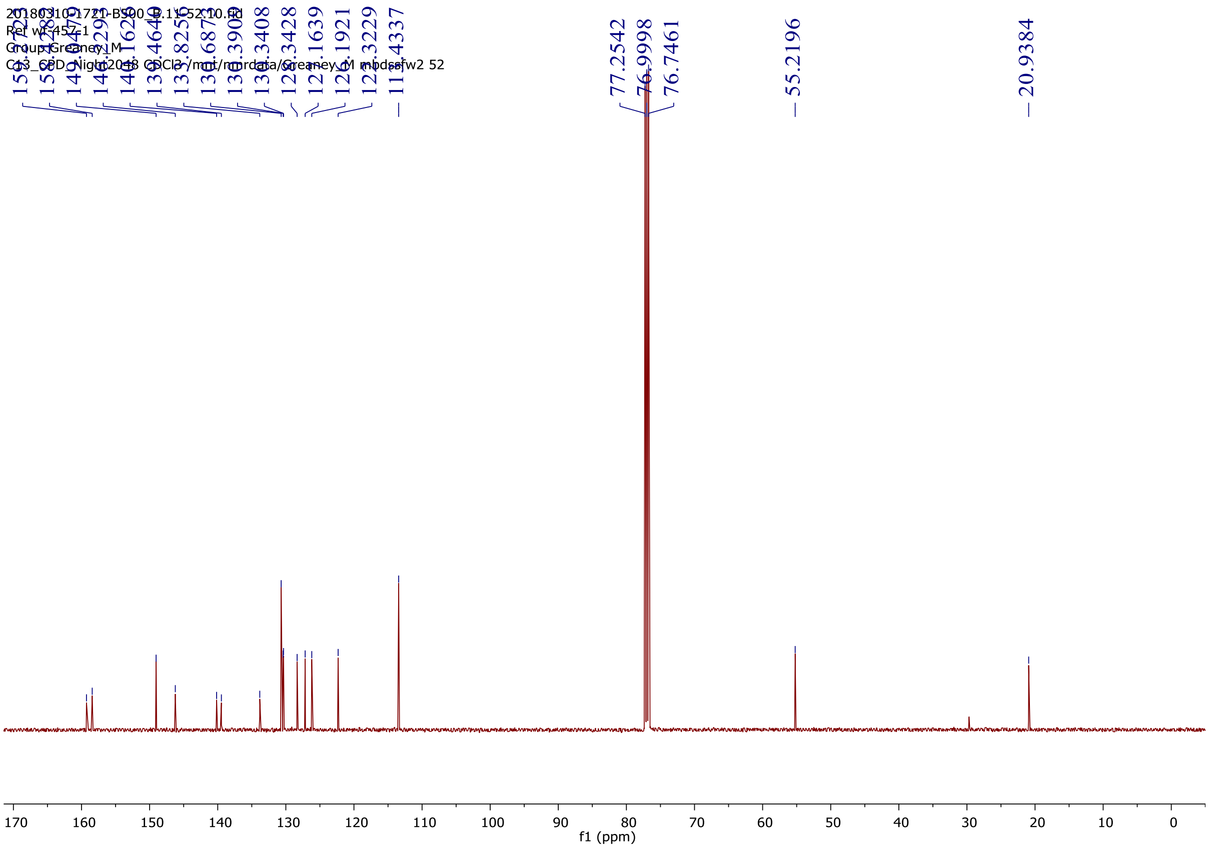
Ref wf-457-1
Group Greaney_M
77.2542
76.7461
55.2196
20.9384
170 160 150 140 130 120 110 100 90 80 70 60 50 40 30 20 10 0
f1 (ppm)

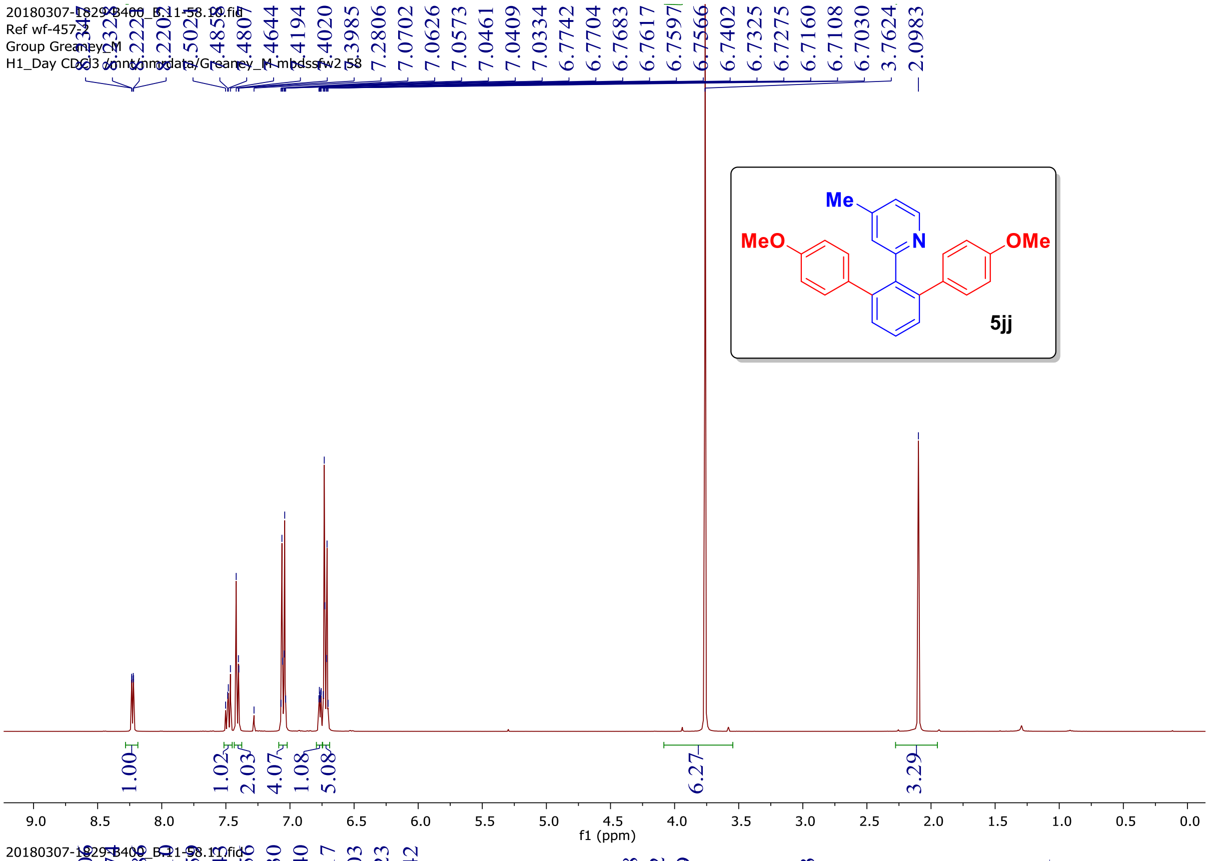

20180307-1829-B400_B.11-58.10.fid
Ref wf-457-2
Group Greaney_M
H1_Day CDCl3 /mnt/nmrdata/Greaney_M mbdssfw2 58
7.4644
7.4194
7.4020
7.3985
7.2806
7.0702
7.0626
7.0573
7.0461
7.0409
7.0334
6.7742
6.7704
6.7683
6.7617
6.7597
6.7566
6.7402
6.7325
6.7275
6.7160
6.7108
6.7030
3.7624
2.0983
Me
N
MeO
OMe
5jj
1.00
1.02
2.03
4.07
1.08
5.08
6.27
3.29
f1 (ppm)

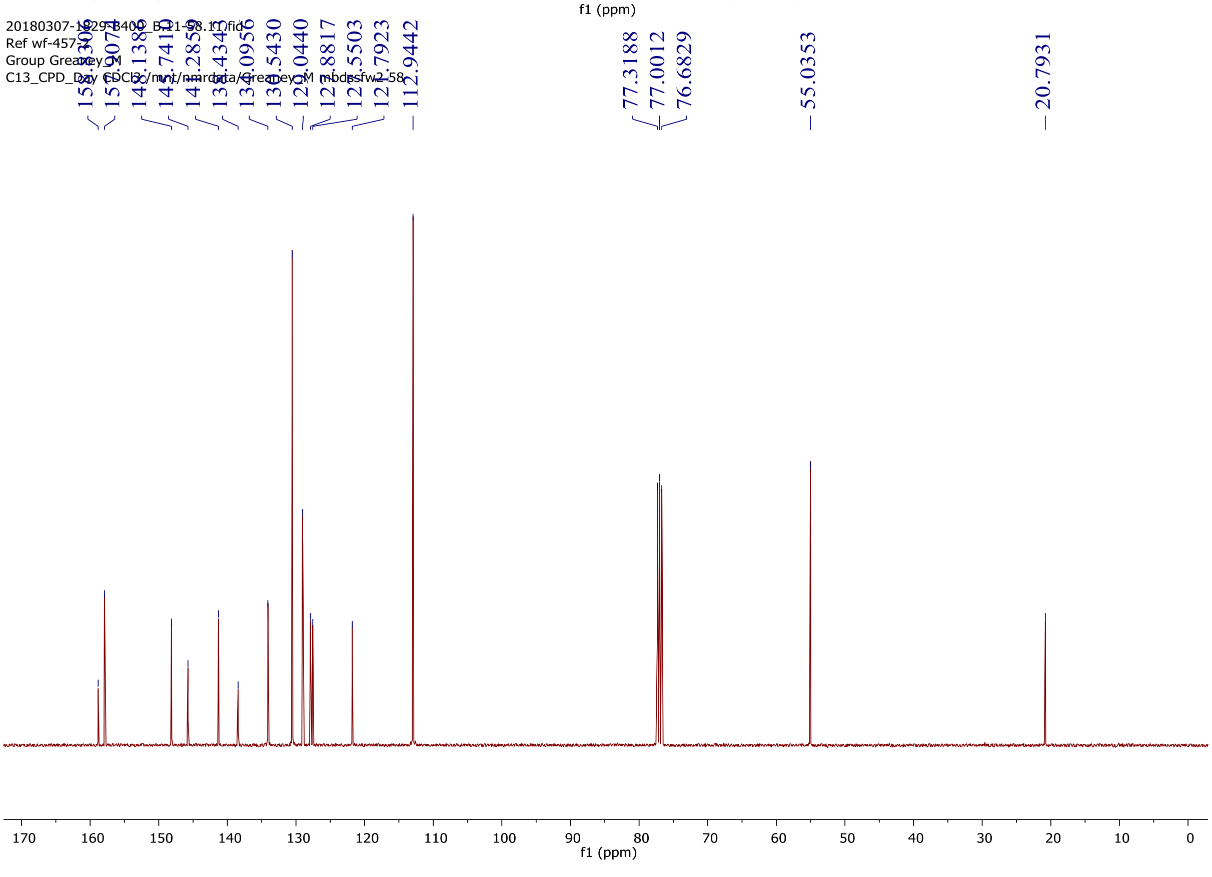

20180307-1829-B400_B.11-58.11.fid
Ref wf-457-2
Group Greaney_M
C13_CPD_Day CDCl3 /mnt/nmrdata/Greaney_M mbdssfw2 58
130.5430
77.3188
77.0012
76.6829
55.0353
20.7931
f1 (ppm)

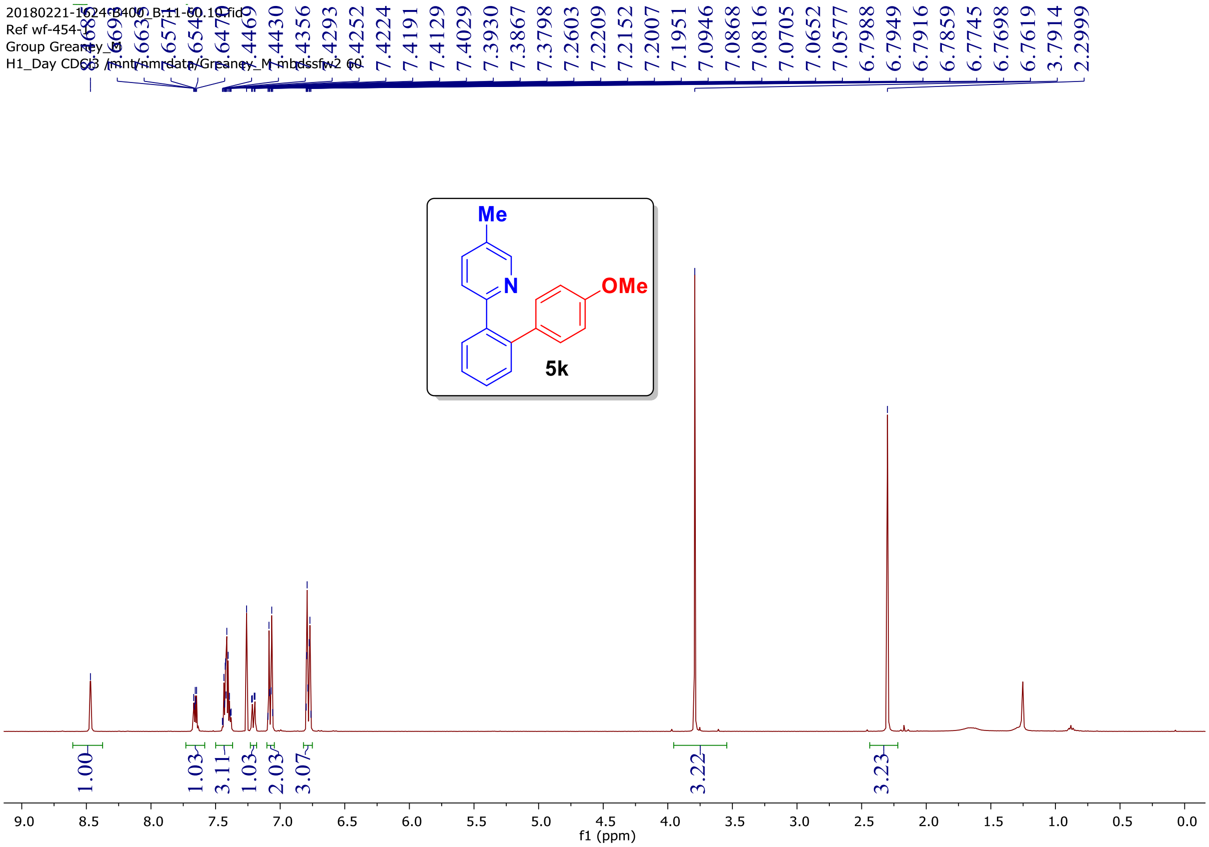
20180221-1624-B400_B-11-60.10.fid
Ref wf-454-
Group Greaney_M
H1_Day CDCl3 /mnt/nmrdata/Greaney_M mbdssfw2 60
7.6631
7.6577
7.6540
7.6479
7.4469
7.4430
7.4356
7.4293
7.4252
7.4224
7.4191
7.4129
7.4029
7.3930
7.3867
7.3798
7.2603
7.2209
7.2152
7.2007
7.1951
7.0946
7.0868
7.0816
7.0705
7.0652
7.0577
6.7988
6.7949
6.7916
6.7859
6.7745
6.7698
6.7619
3.7914
2.2999
Me
N
OMe
5k
1.00
1.03
3.11
1.03
2.03
3.07
3.22
3.23
f1 (ppm)

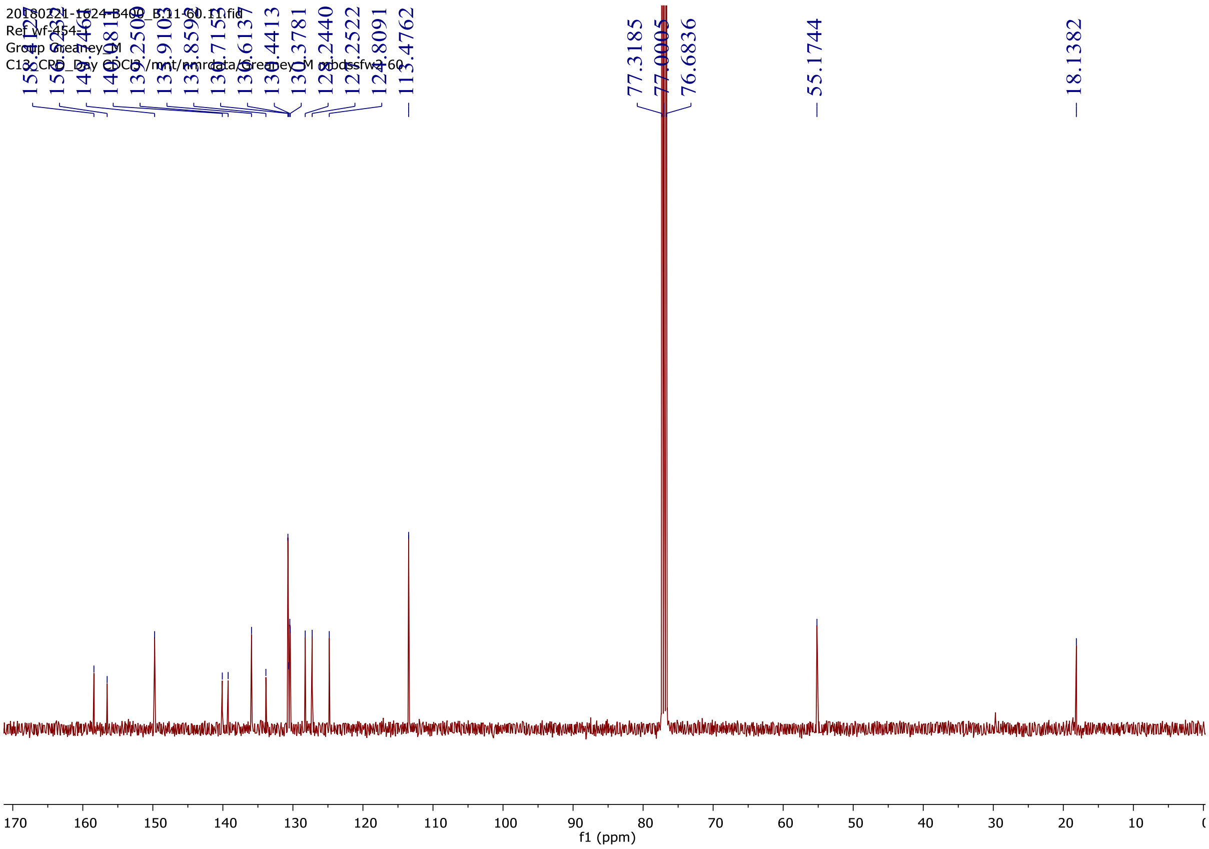
20180221-1624-B400_B-11-60.11.fid
Ref wf-454-
Group Greaney_M
C13_CPD_Day CDCl3 /mnt/nmrdata/Greaney_M mbdssfw2 60
113.4762
77.3185
77.0005
76.6836
55.1744
18.1382
f1 (ppm)

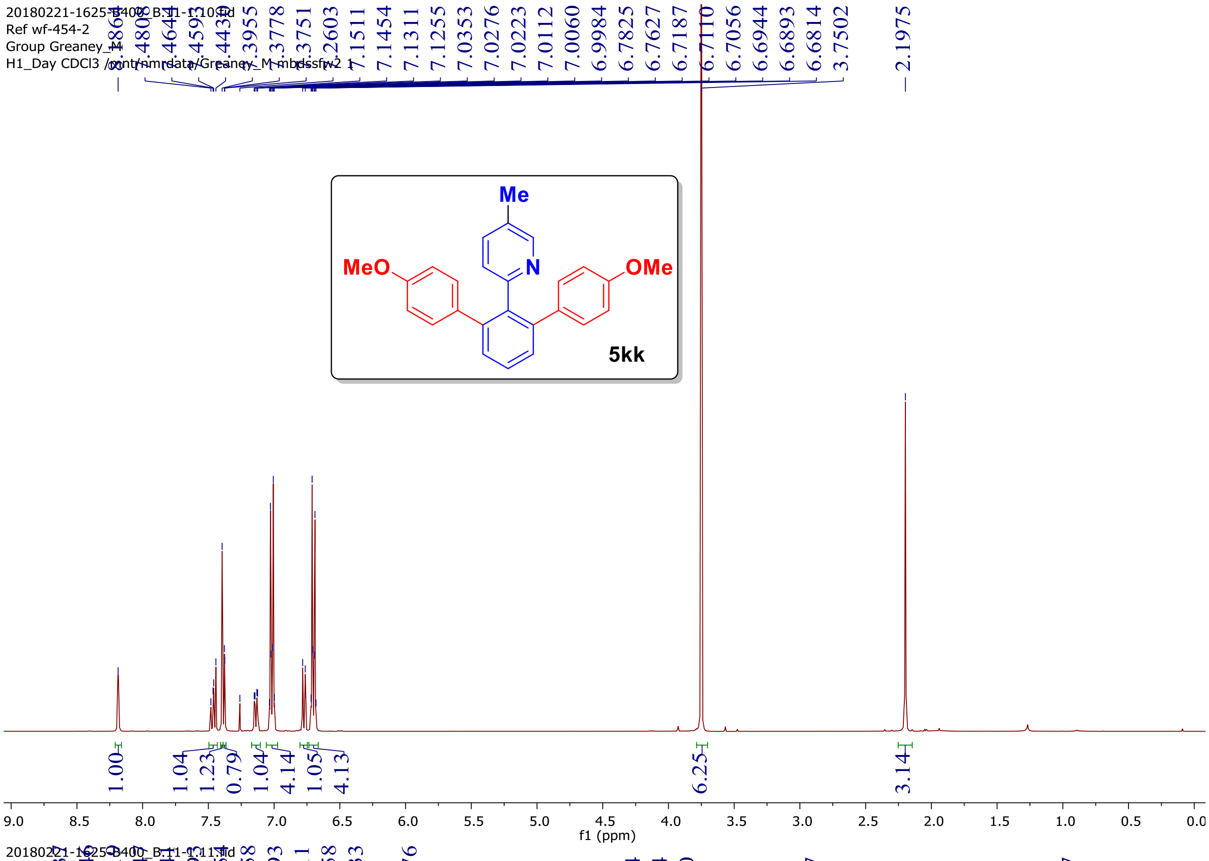
20180221-1625-B400_B.11-1.10.fid
Ref wf-454-2
Group Greaney_M
H1_Day CDCl3 /mnt/nmrdata/Greaney_M mbdssfw2 1
7.3955
7.3778
7.3751
7.2603
7.1511
7.1454
7.1311
7.1255
7.0353
7.0276
7.0223
7.0112
7.0060
6.9984
6.7825
6.7627
6.7187
6.7110
6.7056
6.6944
6.6893
6.6814
3.7502
2.1975
Me
N
MeO
OMe
5kk
1.00
1.04
1.23
0.79
1.04
4.14
1.05
4.13
6.25
3.14
f1 (ppm)

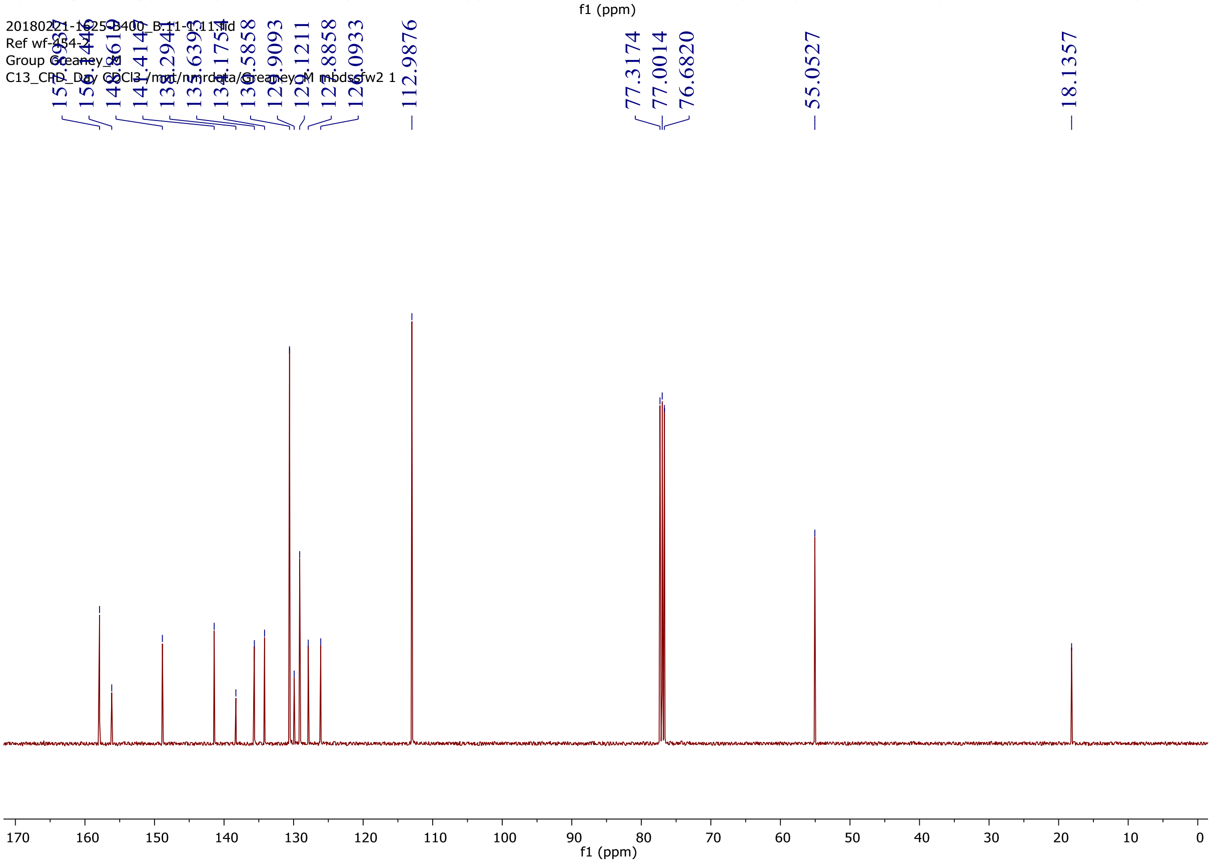
20180221-1625-B400_B.11-1.11.fid
Ref wf-454-2
Group Greaney_M
C13_CPD_Day CDCl3 /mnt/nmrdata/Greaney_M mbdssfw2 1
112.9876
77.3174
77.0014
76.6820
55.0527
18.1357
f1 (ppm)

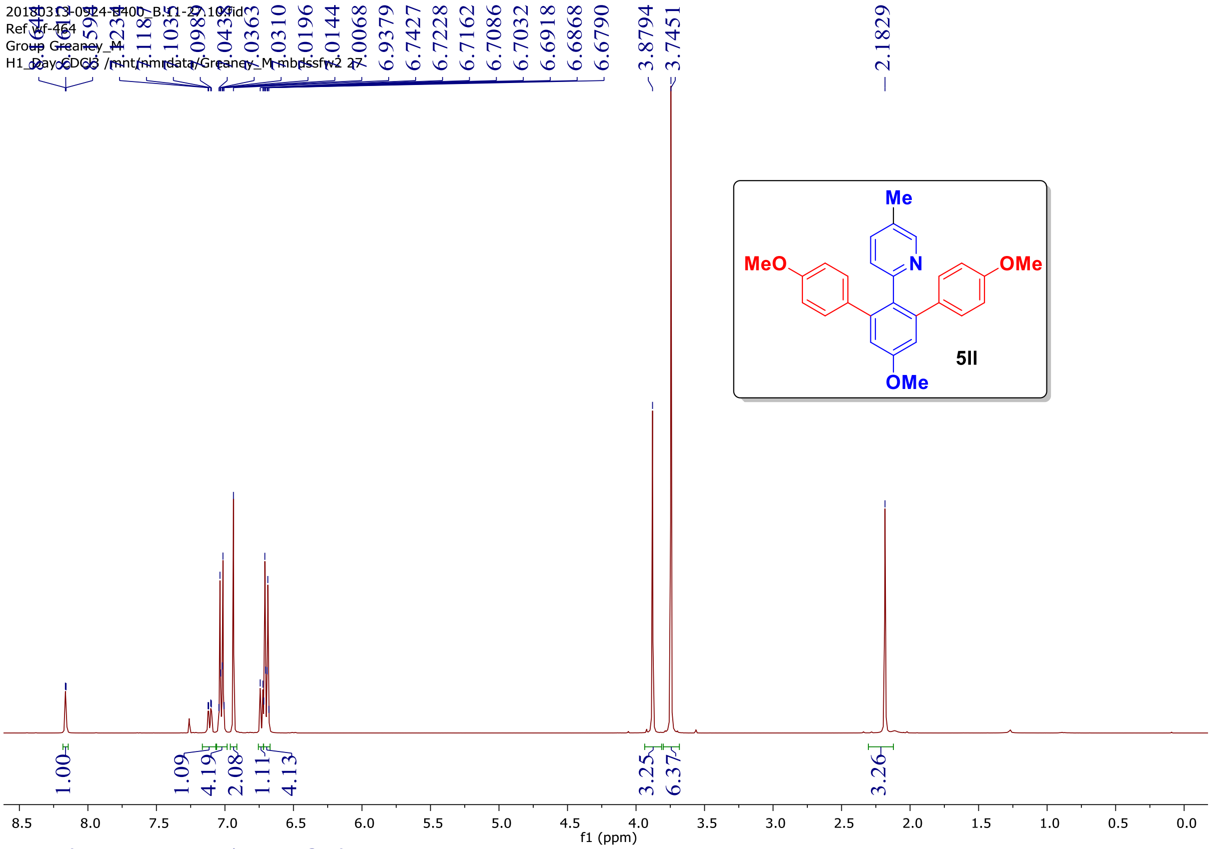
Ref wf-464
Group Greaney_M
6.9379
6.7427
6.7228
6.7162
6.7086
6.7032
6.6918
6.6868
6.6790
3.8794
3.7451
2.1829
Me
N
MeO
OMe
OMe
5ll
1.00
1.09
4.19
2.08
1.11
4.13
3.25
6.37
3.26
f1 (ppm)

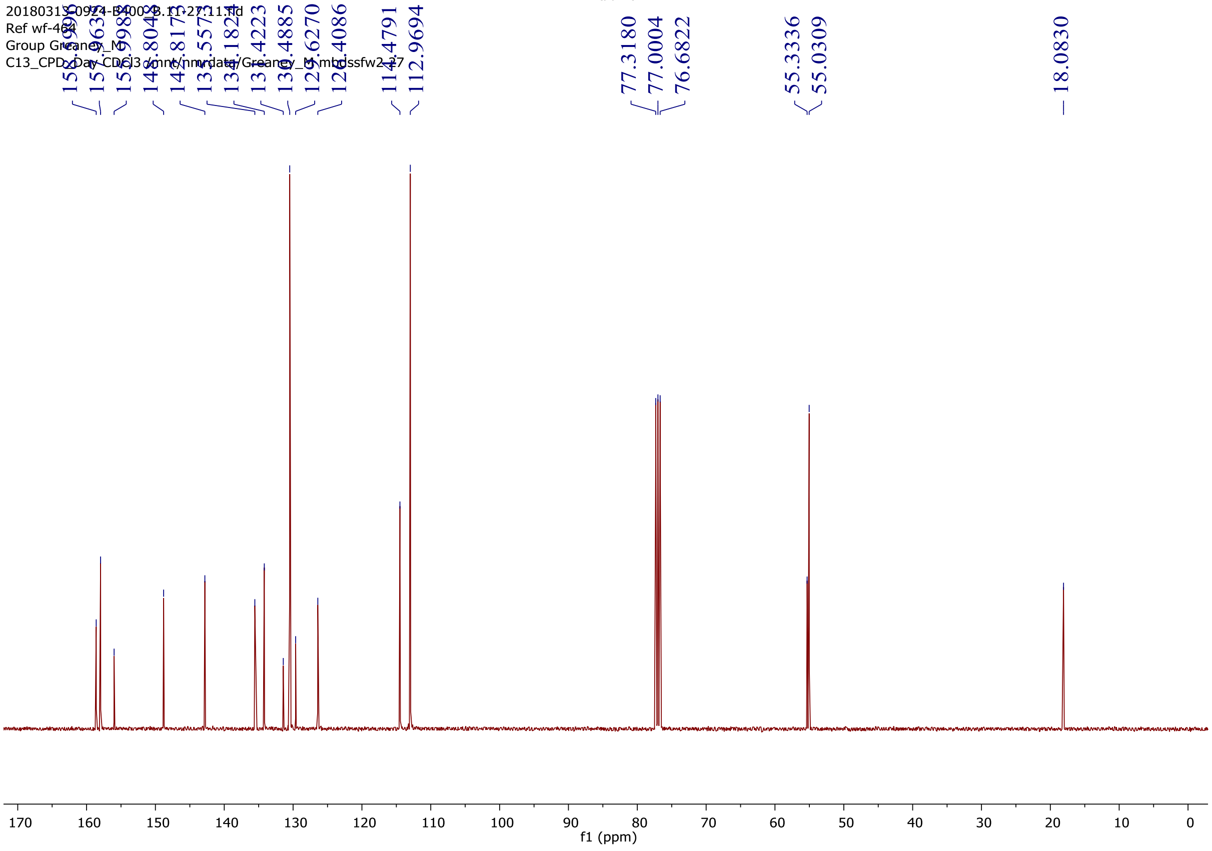
Ref wf-464
Group Greaney_M
77.3180
77.0004
76.6822
55.3336
55.0309
18.0830
f1 (ppm)

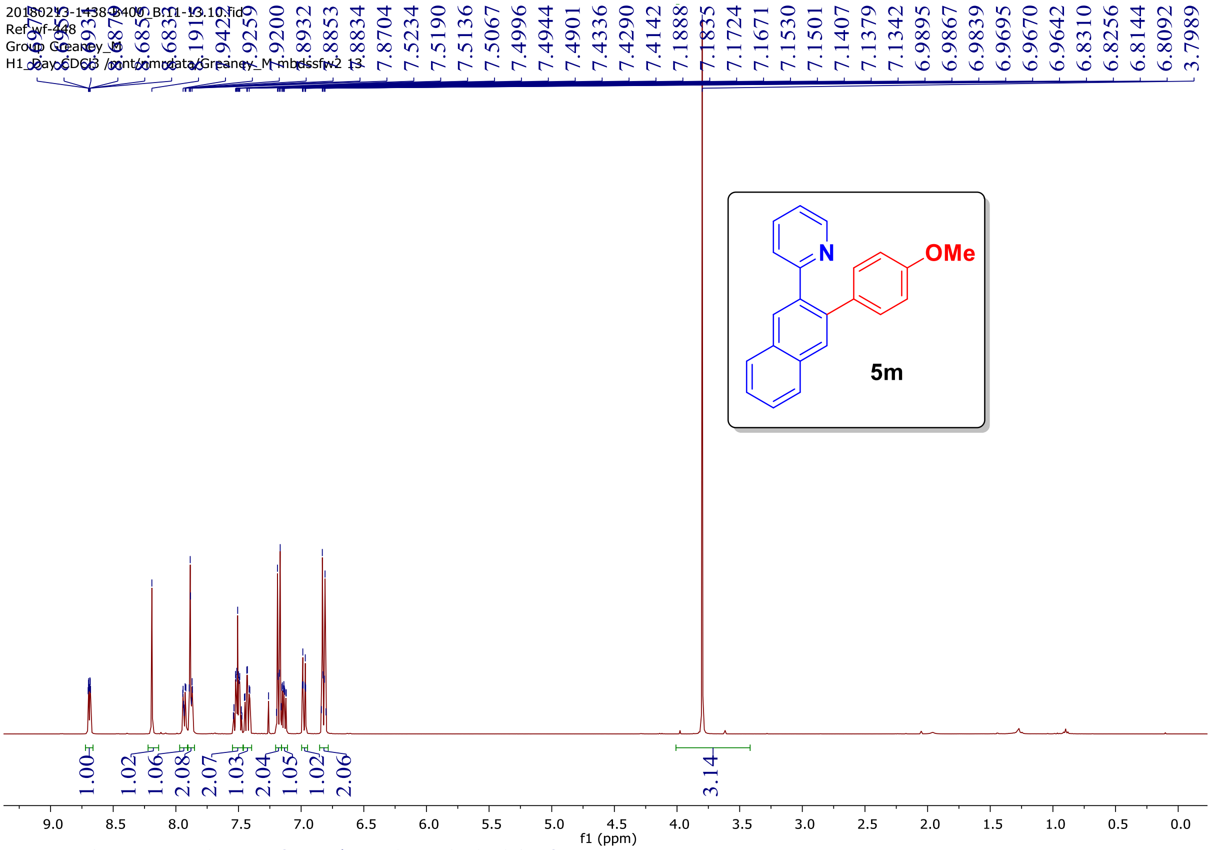
20180213-1438-B400_B.11-13.10.fid
Ref wf-448
Group Greaney_M
H1_Day CDCl3 /mnt/nmrdata/Greaney_M mbdssfw2 13
7.8704 7.5234 7.5190 7.5136 7.5067 7.4996 7.4944 7.4901 7.4336 7.4290 7.4142 7.1888 7.1835 7.1724 7.1671 7.1530 7.1501 7.1407 7.1379 7.1342 6.9895 6.9867 6.9839 6.9695 6.9670 6.9642 6.8310 6.8256 6.8144 6.8092 3.7989
OMe
N
5m
1.00 1.02 1.06 2.08 2.07 1.03 2.04 1.05 1.02 2.06 3.14
f1 (ppm)

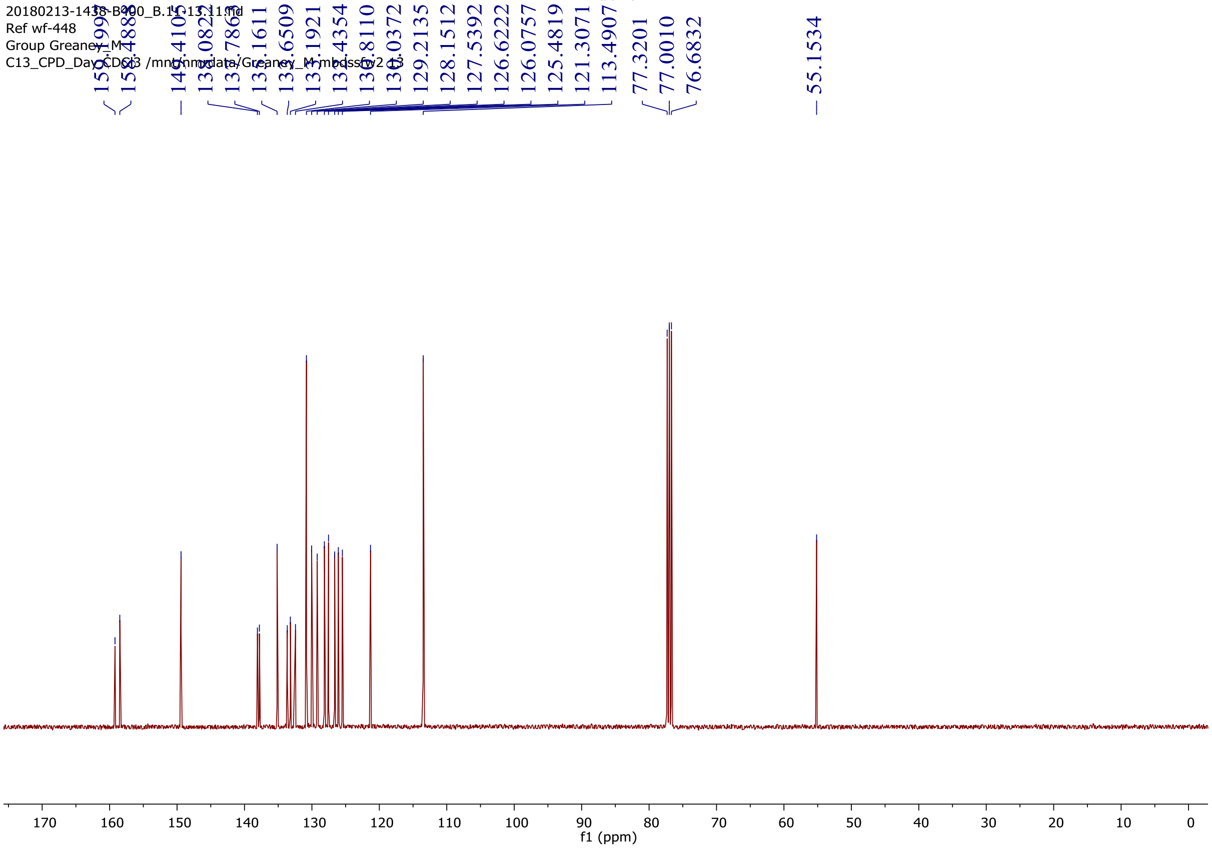
20180213-1438-B400_B.11-13.11.fid
Ref wf-448
Group Greaney_M
C13_CPD_Day CDCl3 /mnt/nmrdata/Greaney_M mbdssfw2 13
129.2135 128.1512 127.5392 126.6222 126.0757 125.4819 121.3071 113.4907 77.3201 77.0010 76.6832 55.1534
f1 (ppm)

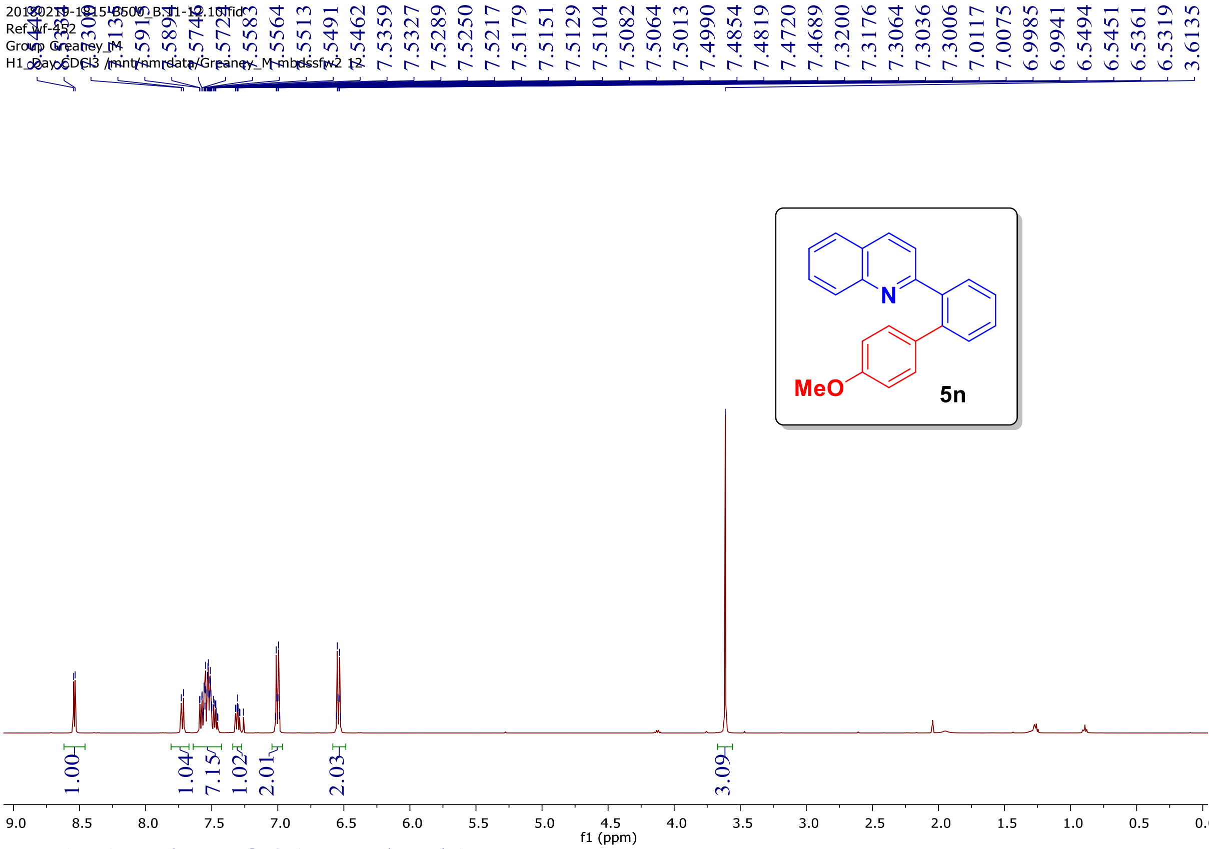
Ref wf-452
Group Greaney_M
7.5359
7.5327
7.5289
7.5250
7.5217
7.5179
7.5151
7.5129
7.5104
7.5082
7.5064
7.5013
7.4990
7.4854
7.4819
7.4720
7.4689
7.3200
7.3176
7.3064
7.3036
7.3006
7.0117
7.0075
6.9985
6.9941
6.5494
6.5451
6.5361
6.5319
3.6135
N
MeO
5n
1.00
1.04
7.15
1.02
2.01
2.03
3.09
f1 (ppm)

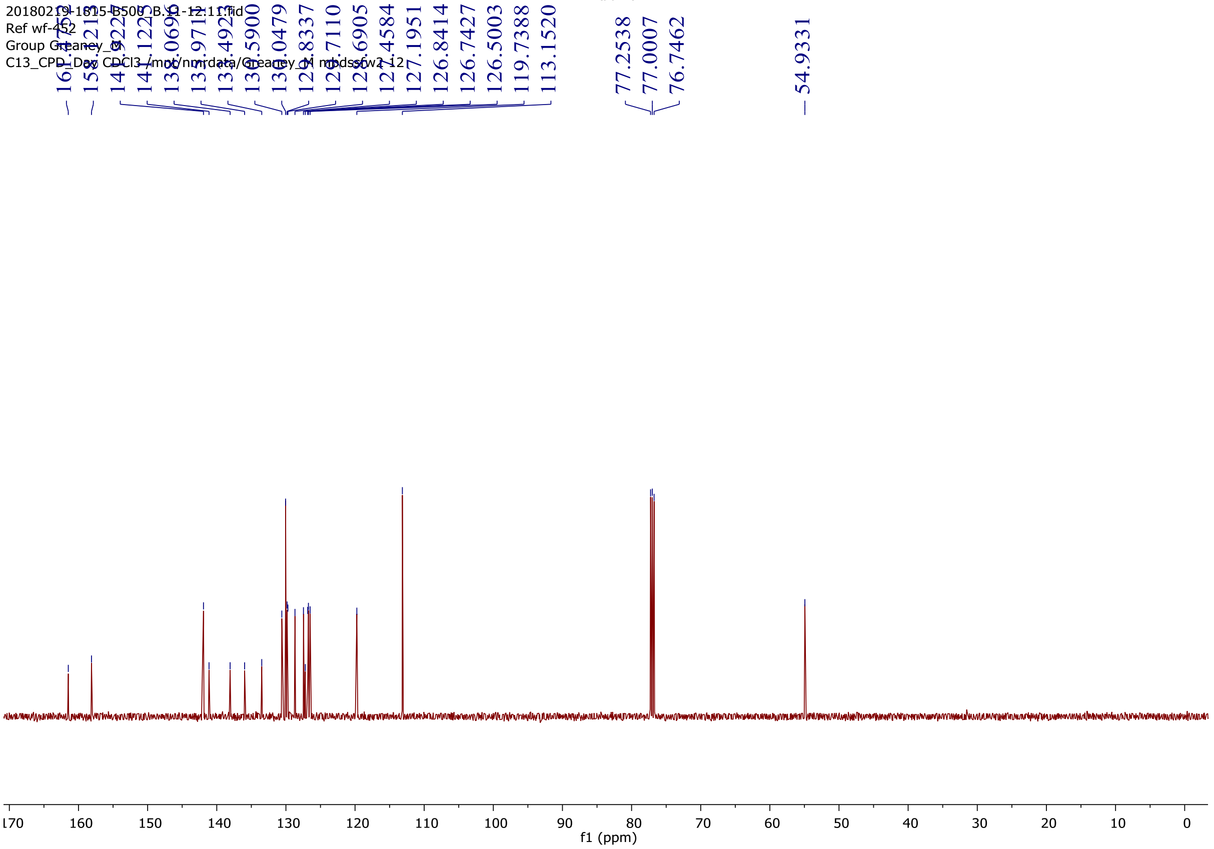
Ref wf-452
127.1951
126.8414
126.7427
126.5003
119.7388
113.1520
77.2538
77.0007
76.7462
54.9331
f1 (ppm)

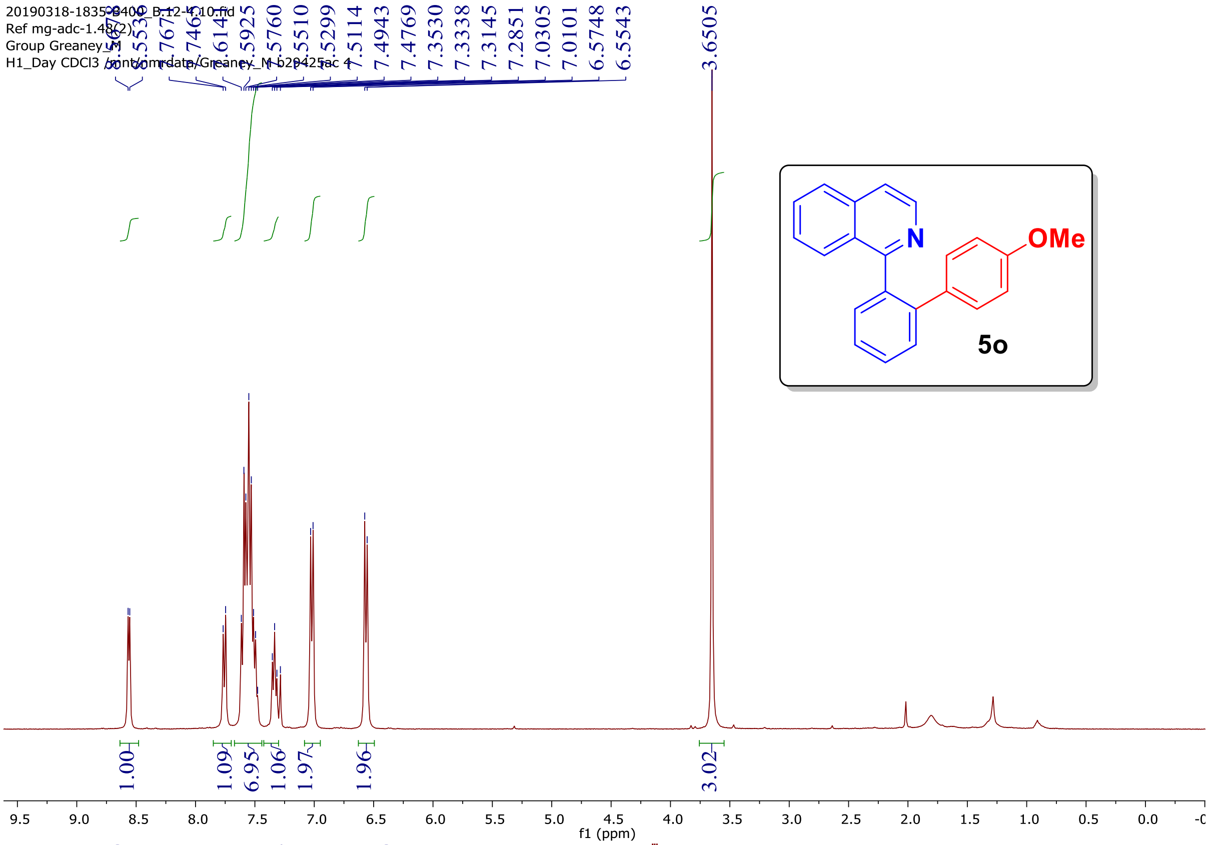
20190318-1835-B400_B.12-4.10.fid
Ref mg-adc-1.48(2)
Group Greaney_M
H1_Day CDCl3 /mnt/nmrdata/Greaney_M b29425ac 4
7.5925
7.5760
7.5510
7.5299
7.5114
7.4943
7.4769
7.3530
7.3338
7.3145
7.2851
7.0305
7.0101
6.5748
6.5543
3.6505
1.00
1.09
6.95
1.06
1.97
1.96
3.02
f1 (ppm)
N
OMe
5o

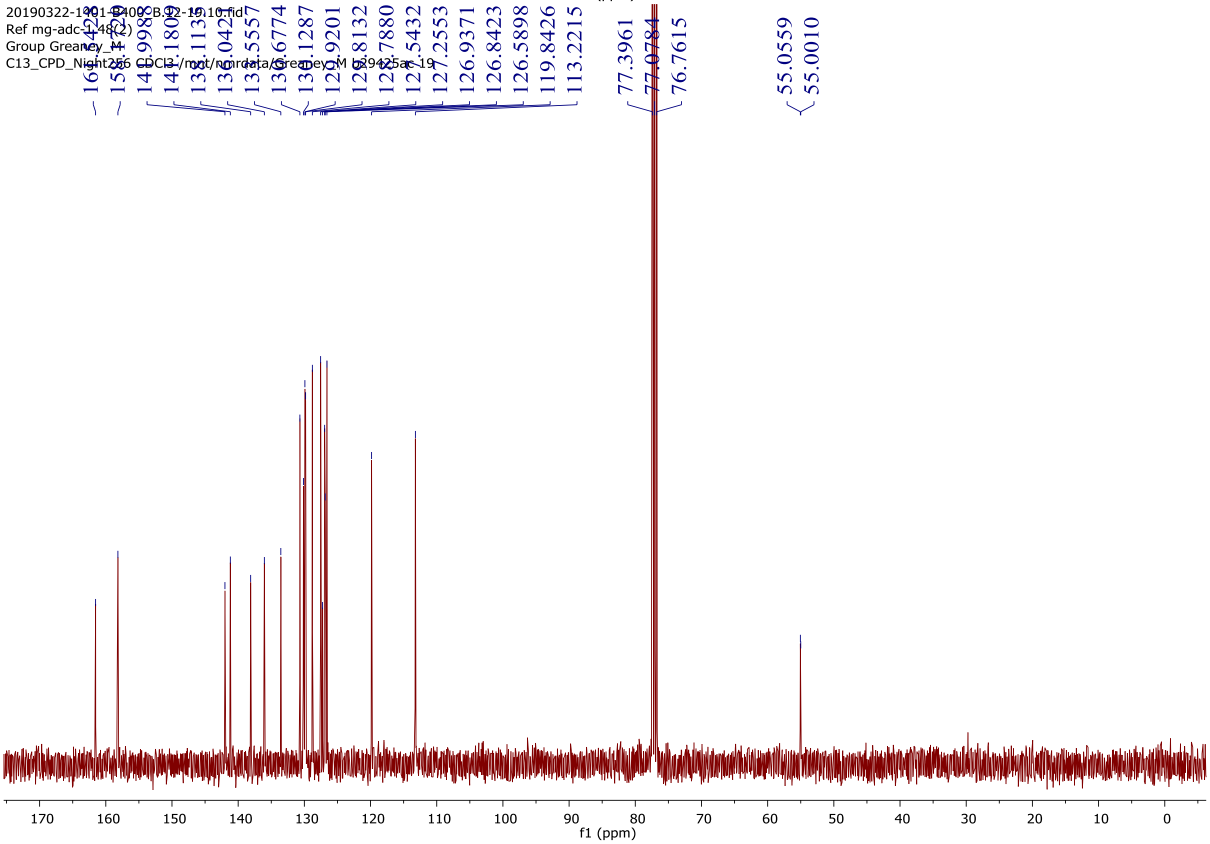
Ref mg-adc-1.48(2)
Group Greaney_M
130.1287
129.9201
128.8132
127.7880
127.5432
127.2553
126.9371
126.8423
126.5898
119.8426
113.2215
77.3961
76.7615
55.0559
55.0010
f1 (ppm)

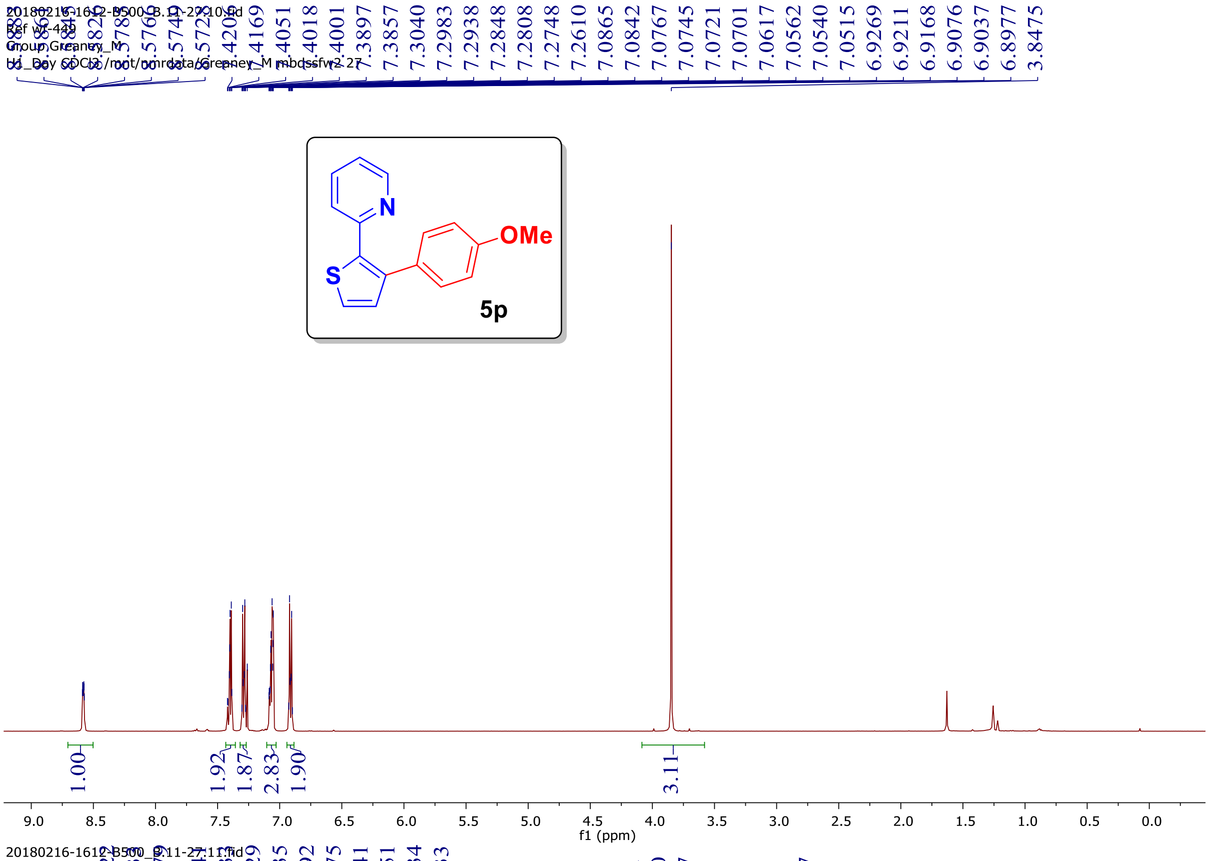
Ref wf-449
7.4169
7.4051
7.4018
7.4001
7.3897
7.3857
7.3040
7.2983
7.2938
7.2848
7.2808
7.2748
7.2610
7.0865
7.0842
7.0767
7.0745
7.0721
7.0701
7.0617
7.0562
7.0540
7.0515
6.9269
6.9211
6.9168
6.9076
6.9037
6.8977
3.8475
OMe
N
S
5p
1.00
1.92
1.87
2.83
1.90
3.11
f1 (ppm)

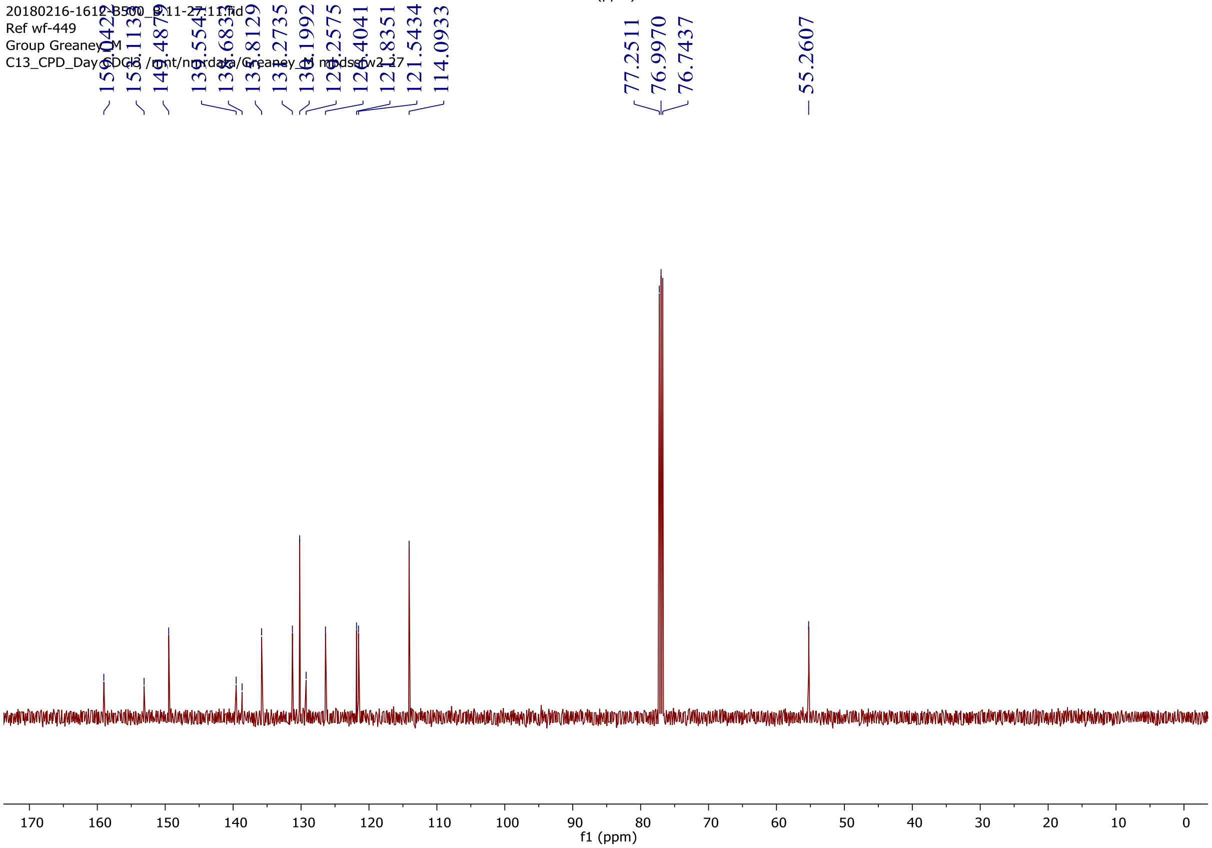
Ref wf-449
Group Greaney_M
121.5434
114.0933
77.2511
76.9970
76.7437
55.2607
f1 (ppm)

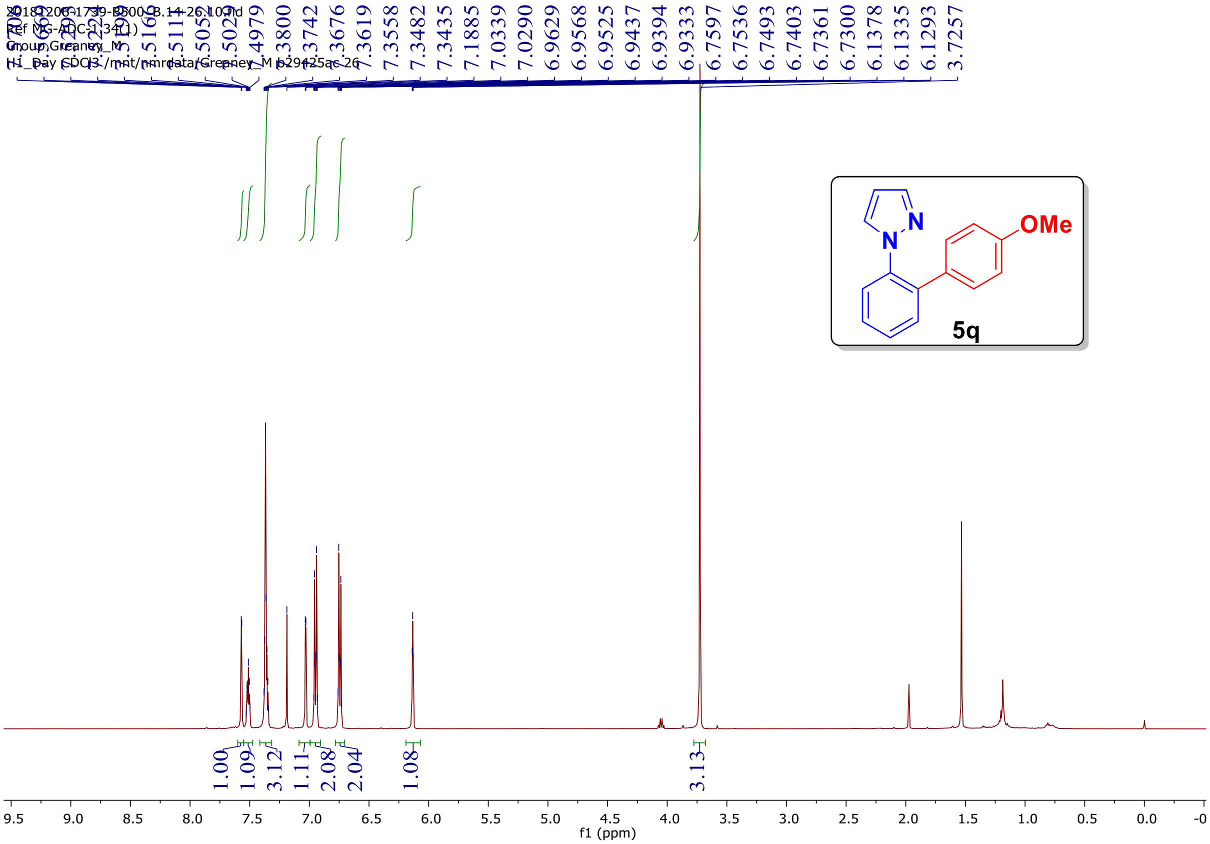

Ref MG-ADC-1.34(1)
7.4979
7.3800
7.3742
7.3676
7.3619
7.3558
7.3482
7.3435
7.1885
7.0339
7.0290
6.9629
6.9568
6.9525
6.9437
6.9394
6.9333
6.7597
6.7536
6.7493
6.7403
6.7361
6.7300
6.1378
6.1335
6.1293
3.7257
N
N
OMe
5q
1.00
1.09
3.12
1.11
2.08
2.04
1.08
3.13
f1 (ppm)

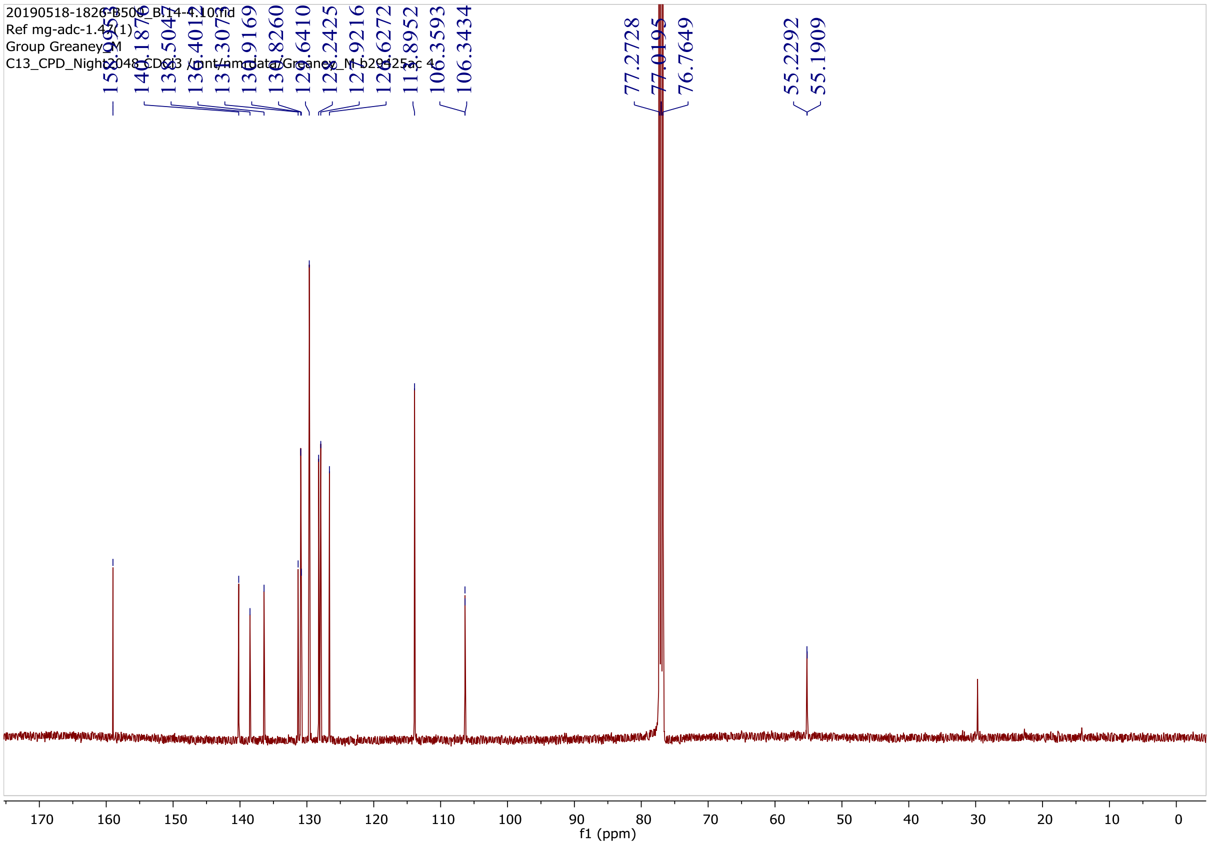

Ref mg-adc-1.47(1)
Group Greaney_M
106.3593
106.3434
77.2728
76.7649
55.2292
55.1909
f1 (ppm)

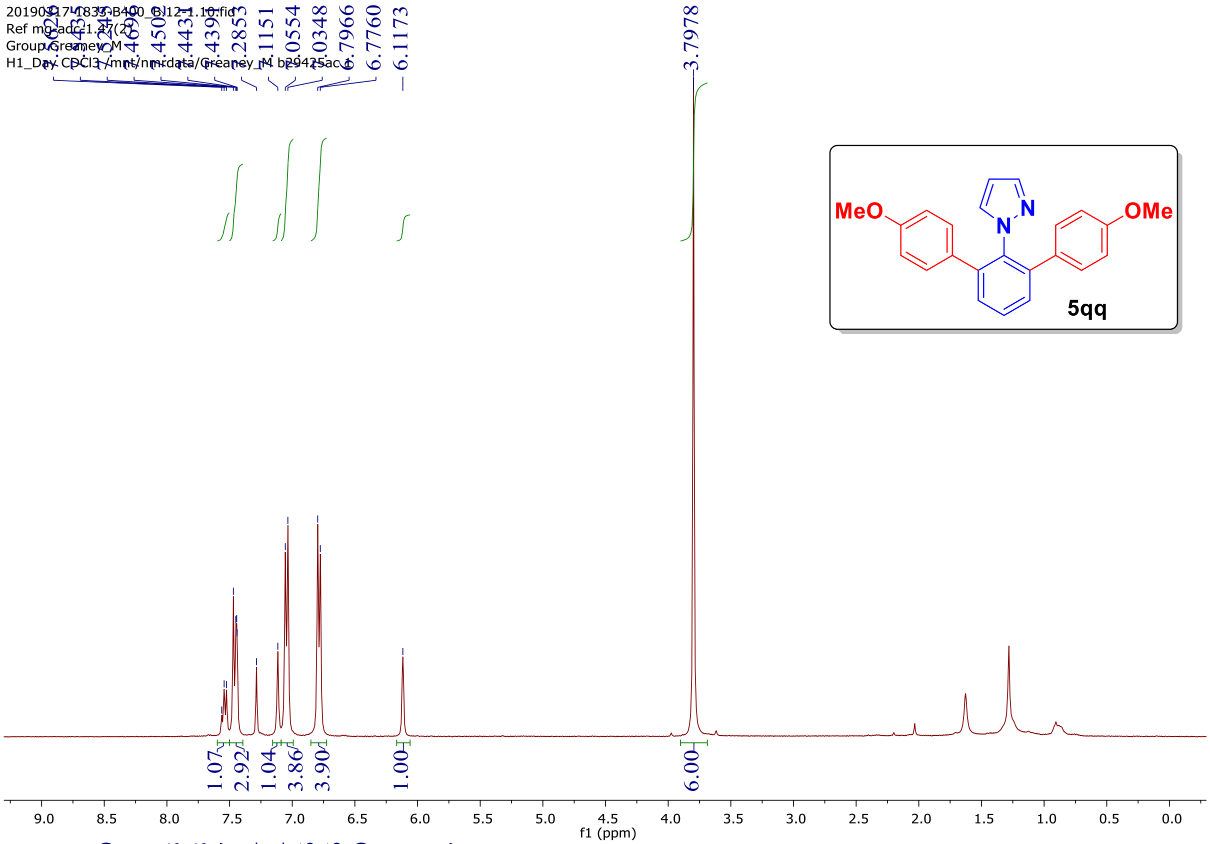
Group Greaney_M
7.1151
7.0554
7.0348
6.7966
6.7760
6.1173
3.7978
MeO
OMe
N
N
5qq
1.07
2.92
1.04
3.86
3.90
1.00
6.00
9.0
8.5
8.0
7.5
7.0
6.5
6.0
5.5
5.0
4.5
4.0
3.5
3.0
2.5
2.0
1.5
1.0
0.5
0.0
f1 (ppm)

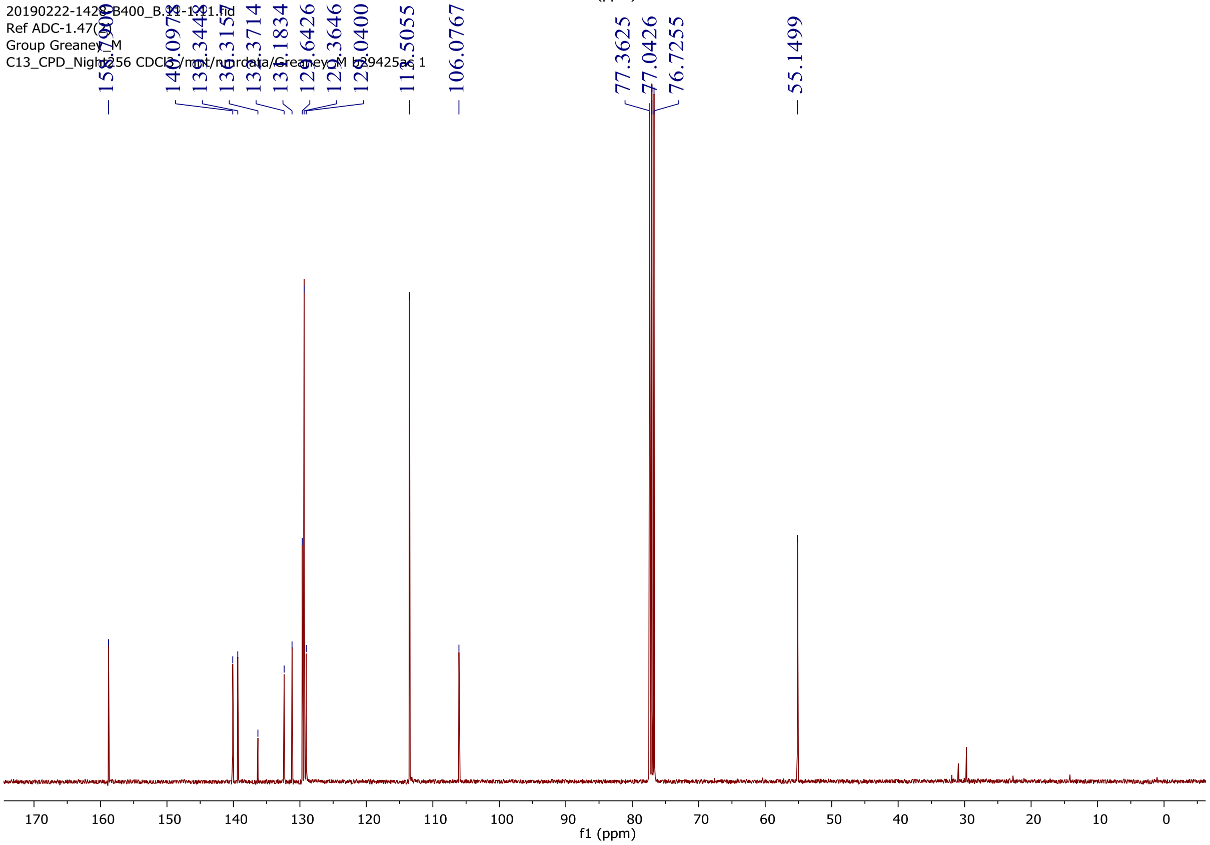
Group Greaney_M
106.0767
77.3625
77.0426
76.7255
55.1499
170
160
150
140
130
120
110
100
90
80
70
60
50
40
30
20
10
0
f1 (ppm)

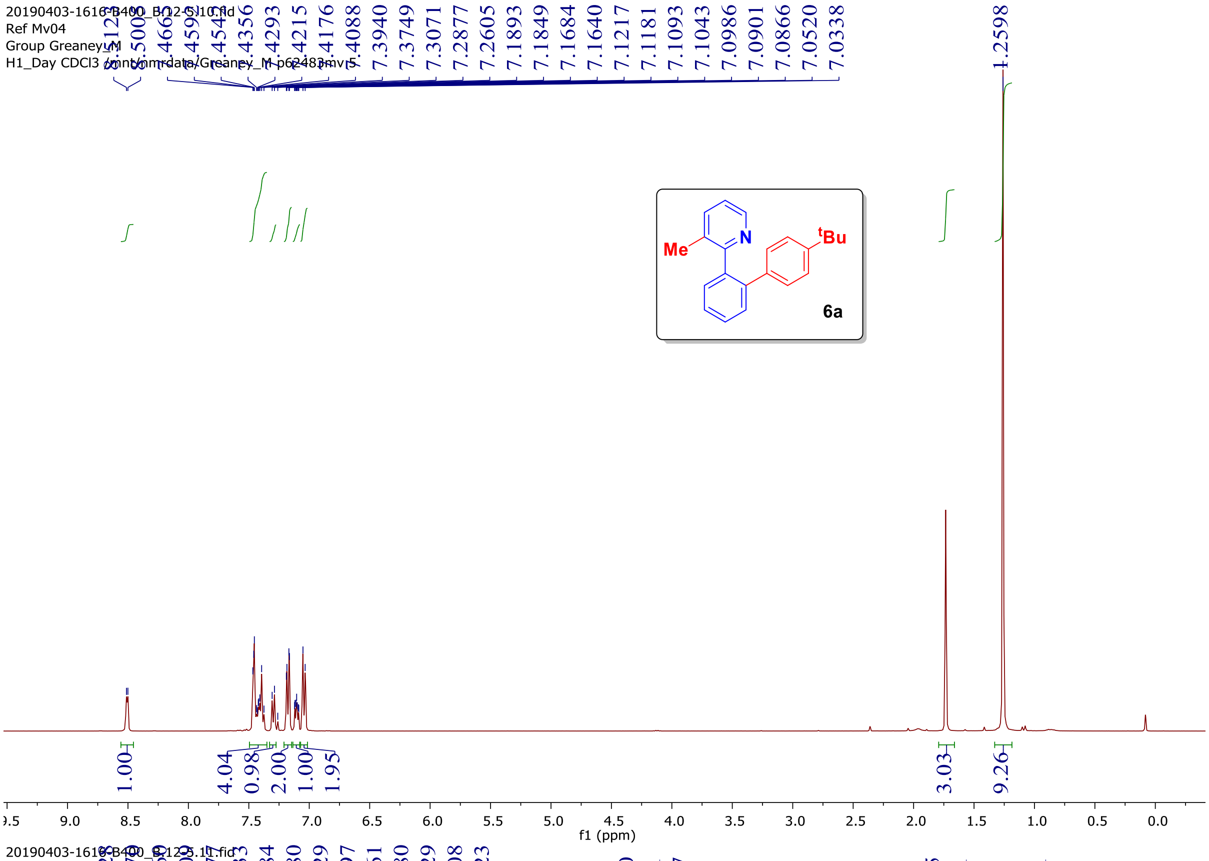

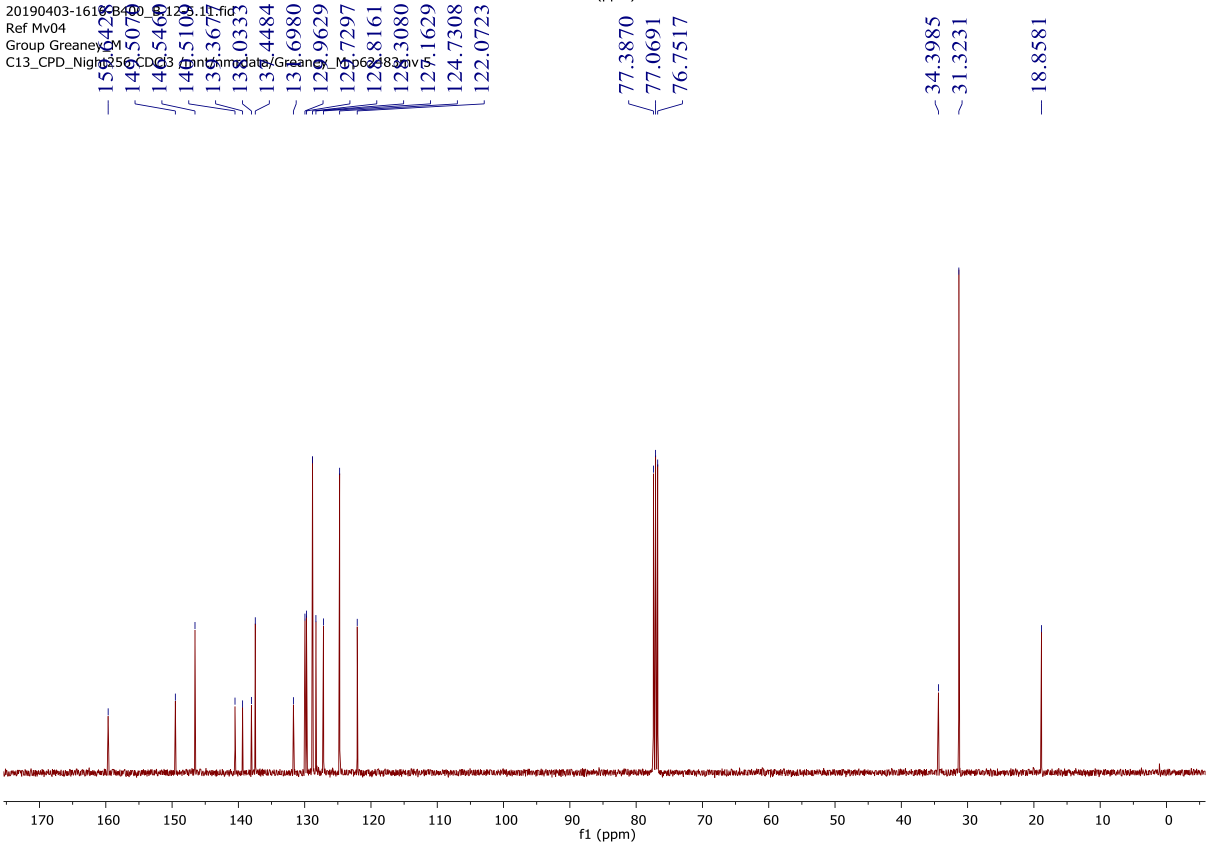

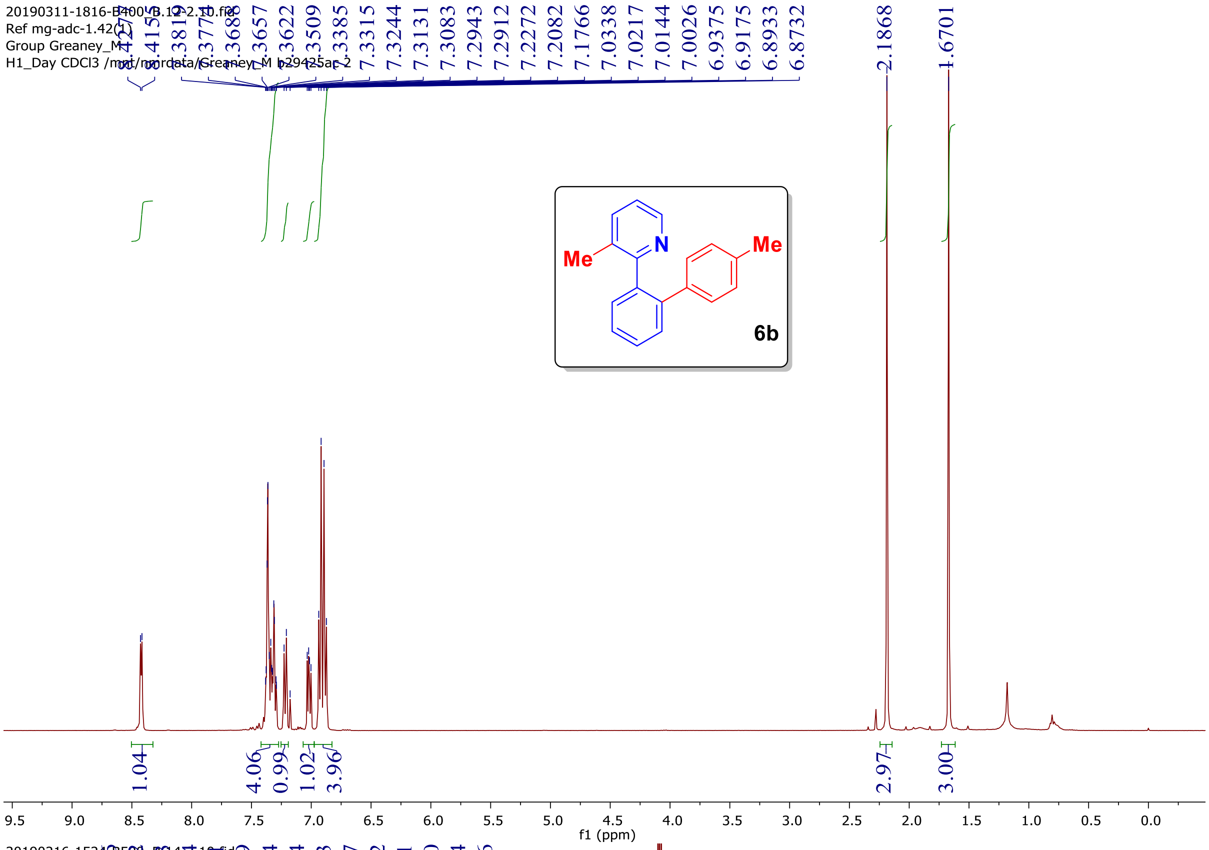
20190311-1816-B400_B.12-2.10.fid
Ref mg-adc-1.42(1)
Group Greaney_M
H1_Day CDCl3 /mnt/nmrdata/Greaney_M b29425ac 2
7.3819
7.3774
7.3688
7.3657
7.3622
7.3509
7.3385
7.3315
7.3244
7.3131
7.3083
7.2943
7.2912
7.2272
7.2082
7.1766
7.0338
7.0217
7.0144
7.0026
6.9375
6.9175
6.8933
6.8732
2.1868
1.6701
Me
N
Me
6b
1.04
4.06
0.99
1.02
3.96
2.97
3.00
f1 (ppm)

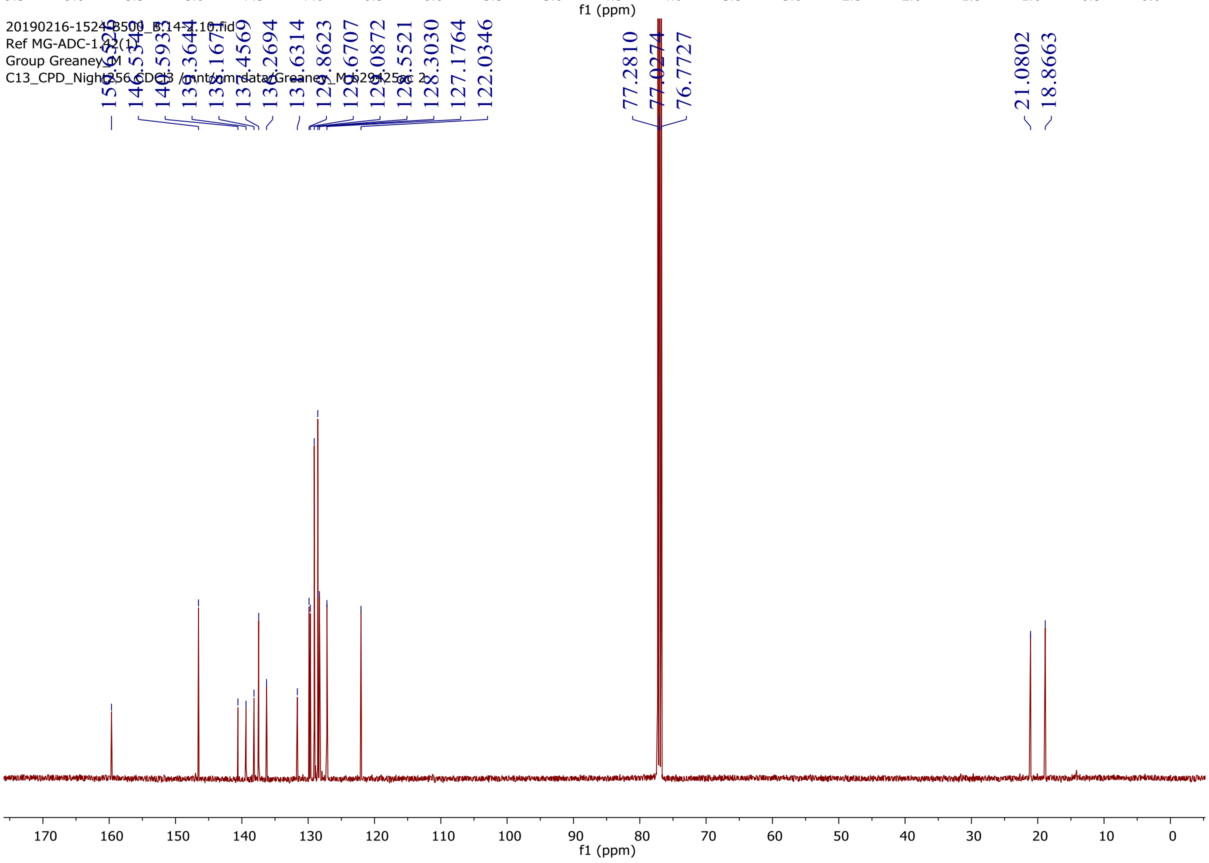
20190216-1524-B500_B.14-1.10.fid
Ref MG-ADC-1.42(1)
Group Greaney_M
C13_CPD_Night256 CDCl3 /mnt/nmrdata/Greaney_M b29425ac 2
127.1764
122.0346
77.2810
77.0274
76.7727
21.0802
18.8663
f1 (ppm)

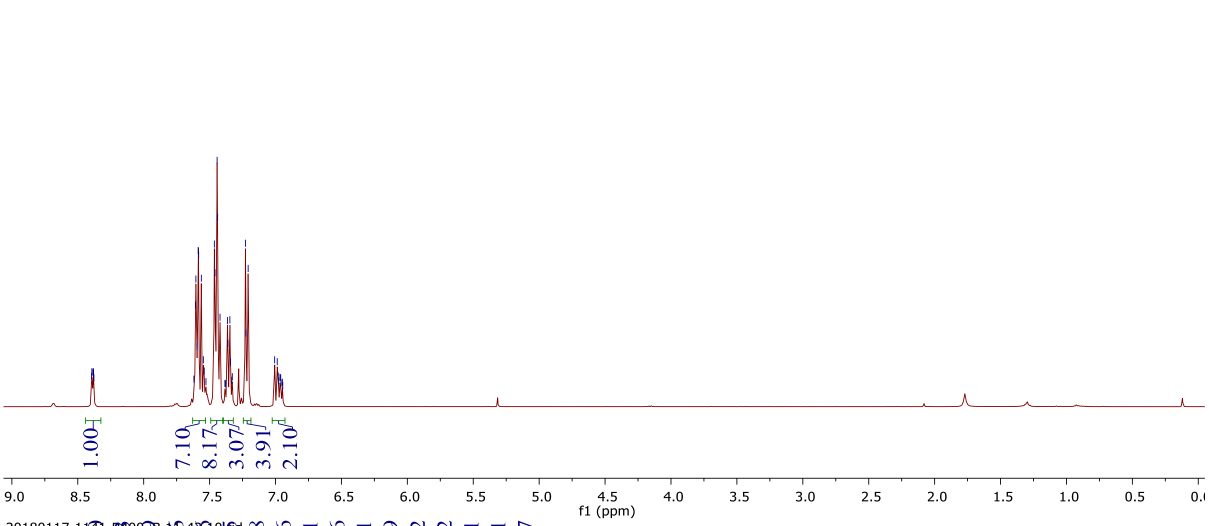

Ref wf-414
Group Greaney_M
7.5864
7.5830
7.5627
7.5473
7.5406
7.5276
7.4640
7.4590
7.4431
7.4391
7.4209
7.3839
7.3795
7.3648
7.3606
7.3462
7.3409
7.3307
7.3277
7.2279
7.2230
7.2075
7.0065
6.9869
6.9792
6.9763
6.9663
6.9605
6.9574
6.9481
6.9451
1.00
7.10
8.17
3.07
3.91
2.10
f1 (ppm)

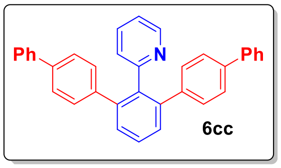

Ph
N
Ph
6cc

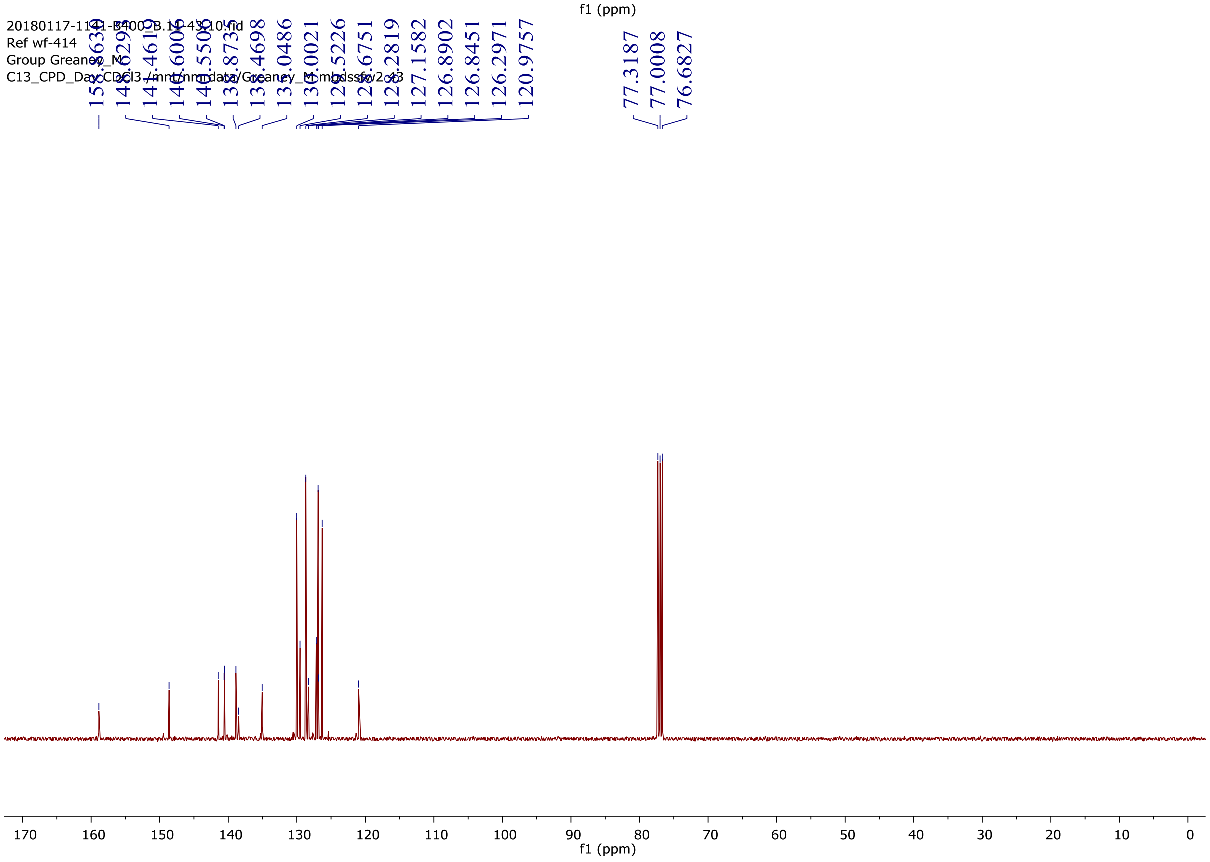

Ref wf-414
130.0021
127.1582
126.8902
126.8451
126.2971
120.9757
77.3187
77.0008
76.6827
f1 (ppm)

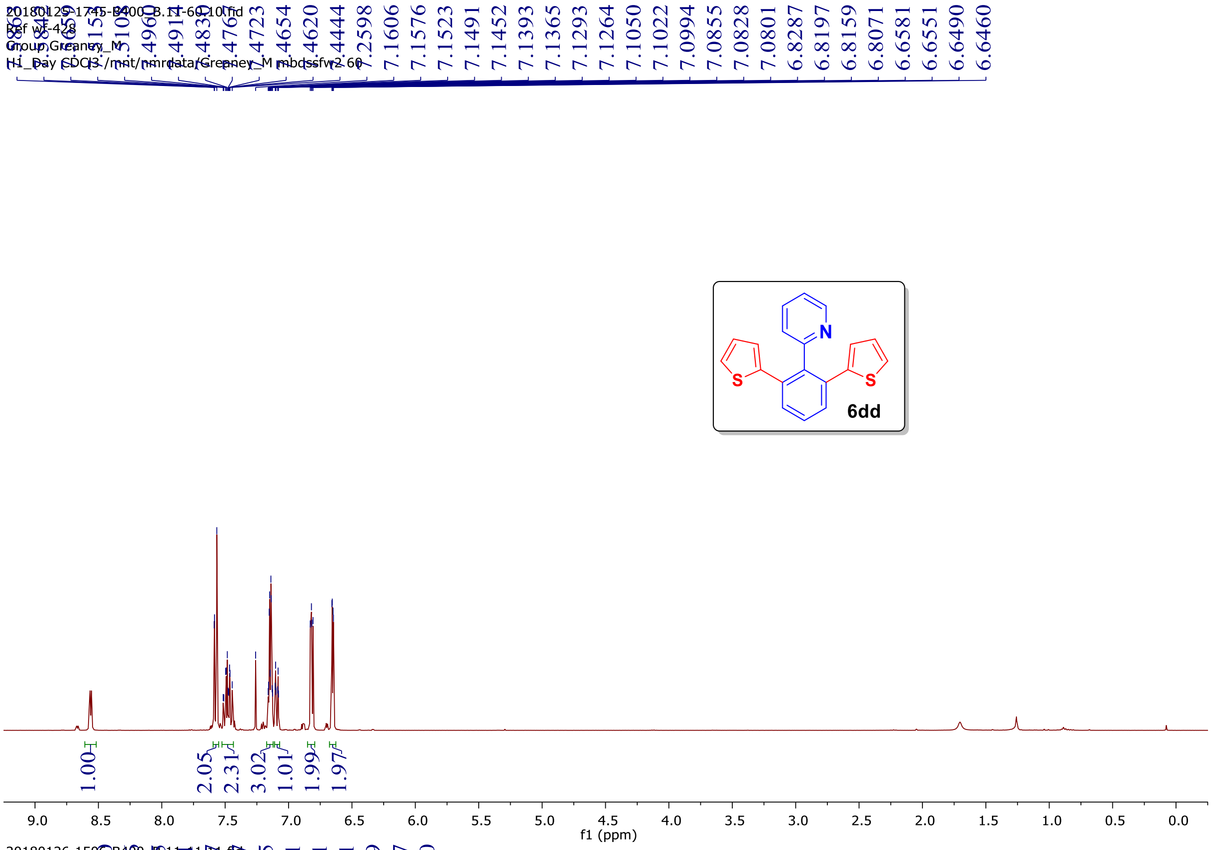

Ref wf-428
Group Greaney_M
7.4723
7.4654
7.4620
7.4444
7.2598
7.1606
7.1576
7.1523
7.1491
7.1452
7.1393
7.1365
7.1293
7.1264
7.1050
7.1022
7.0994
7.0855
7.0828
7.0801
6.8287
6.8197
6.8159
6.8071
6.6581
6.6551
6.6490
6.6460
N
S
S
6dd
1.00
2.05
2.31
3.02
1.01
1.99
1.97
9.0 8.5 8.0 7.5 7.0 6.5 6.0 5.5 5.0 4.5 4.0 3.5 3.0 2.5 2.0 1.5 1.0 0.5 0.0
f1 (ppm)

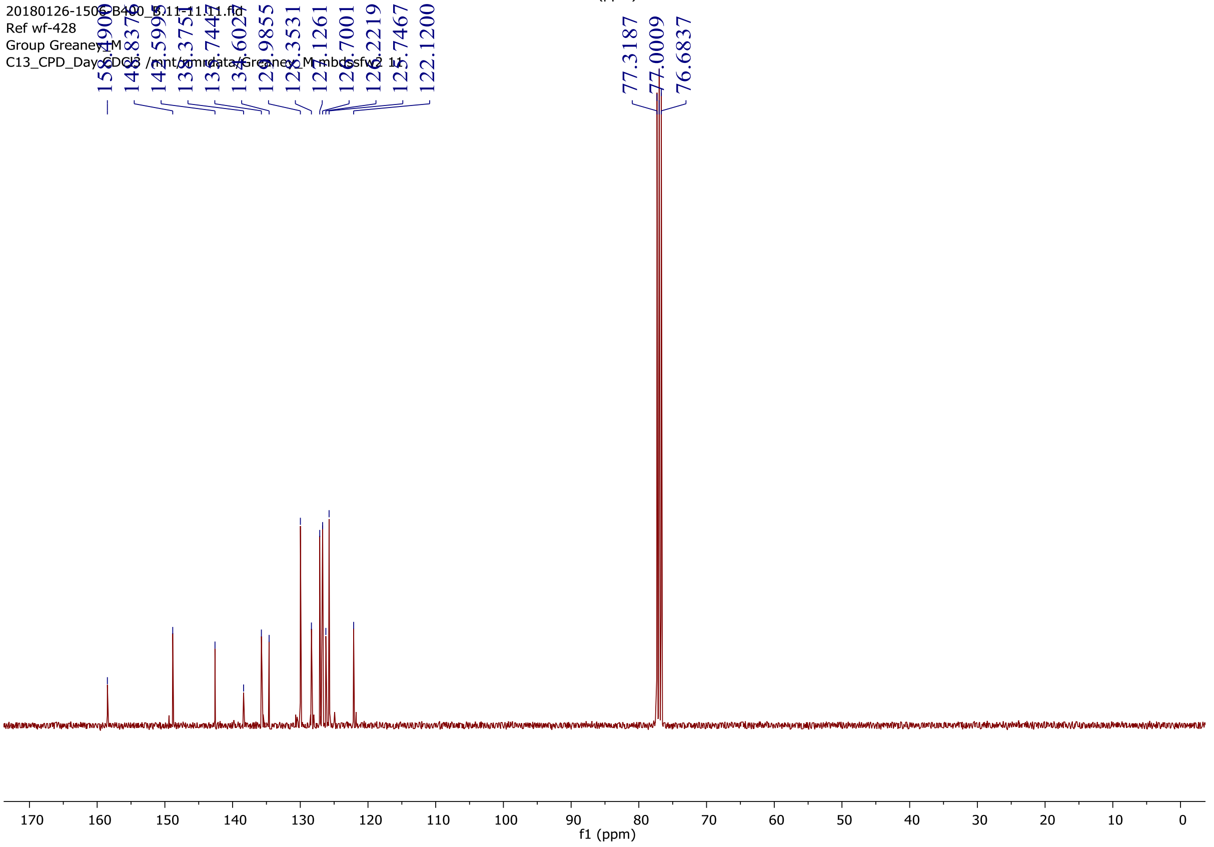

Ref wf-428
129.9855
128.3531
122.1200
77.3187
77.0009
76.6837
170 160 150 140 130 120 110 100 90 80 70 60 50 40 30 20 10 0
f1 (ppm)

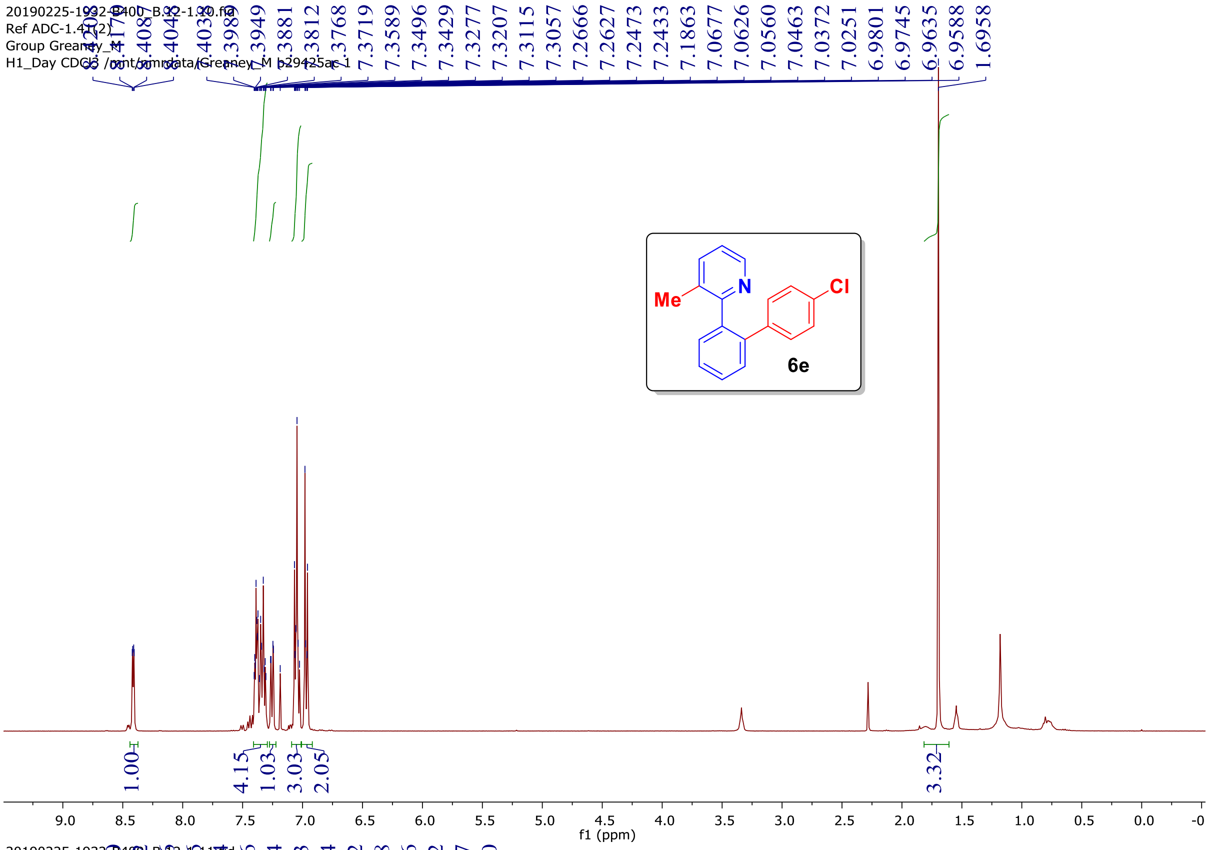

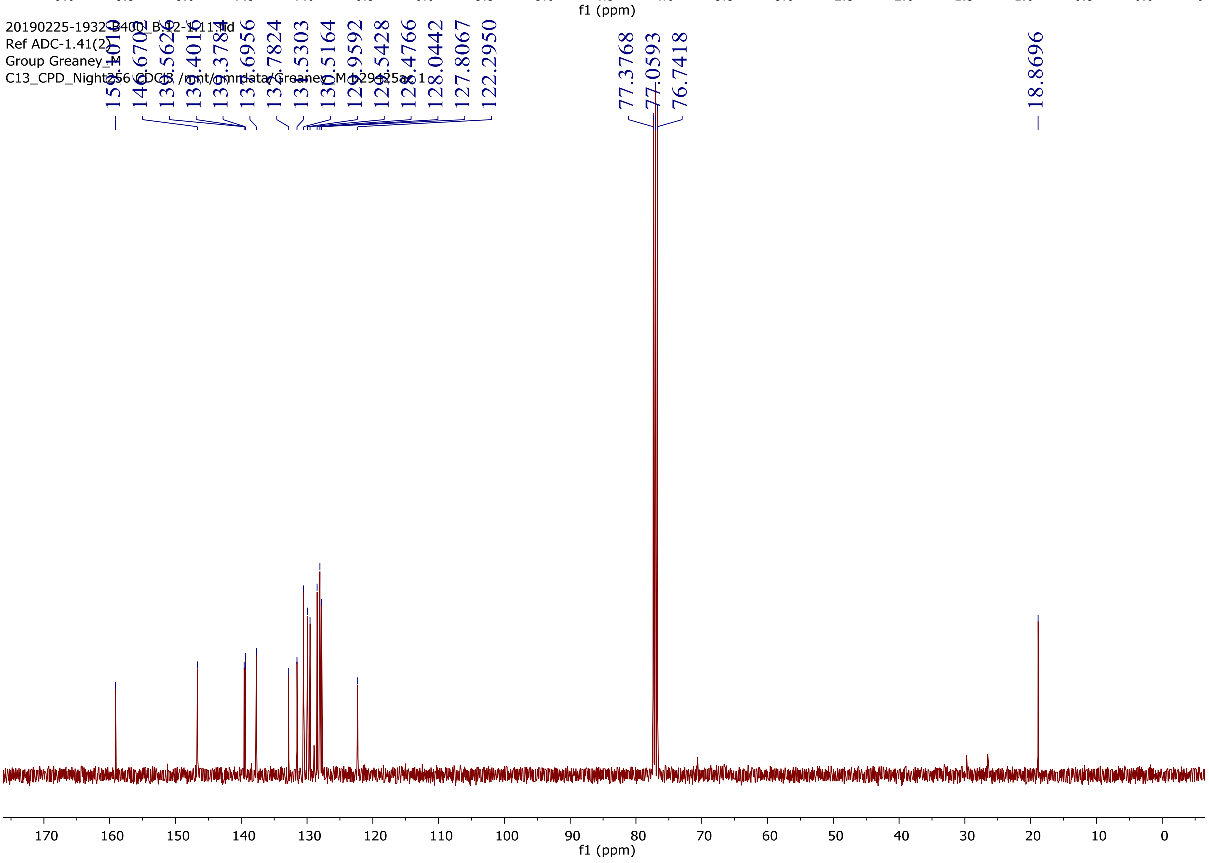

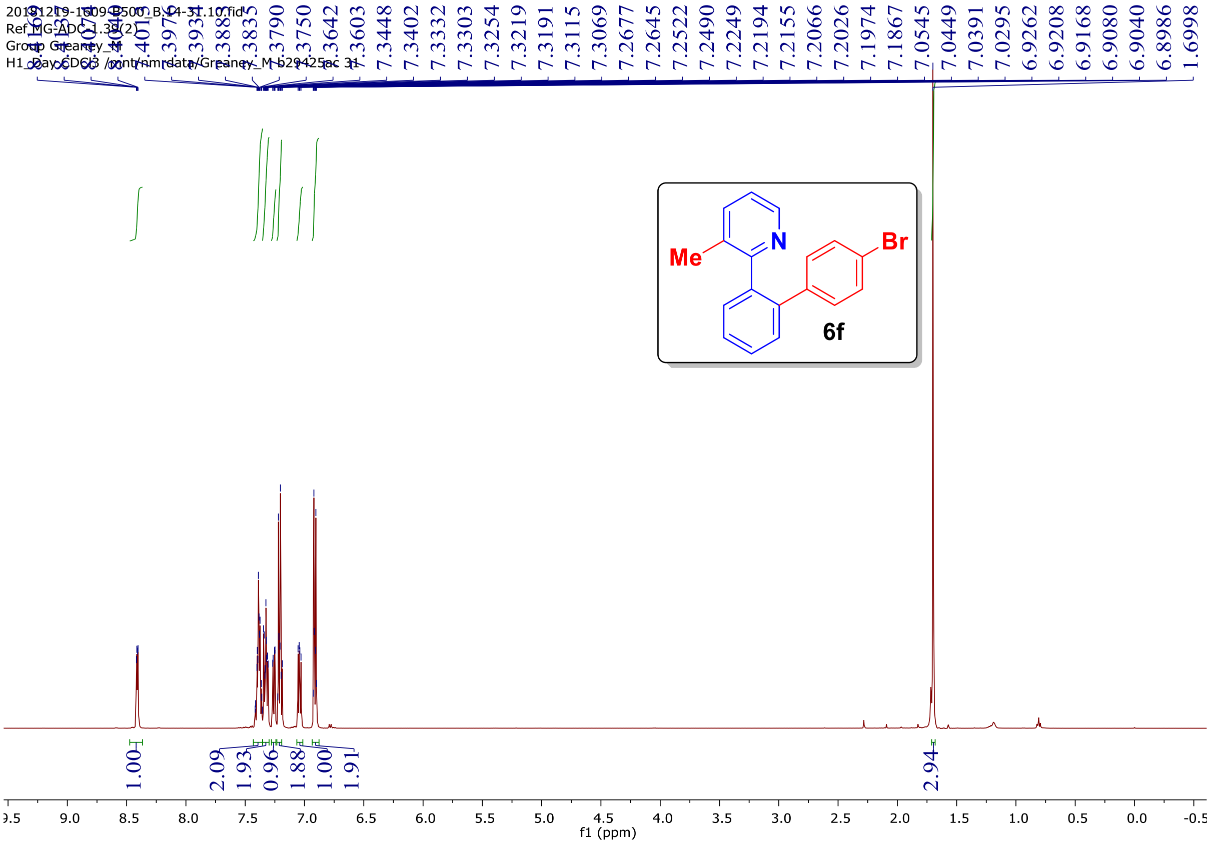

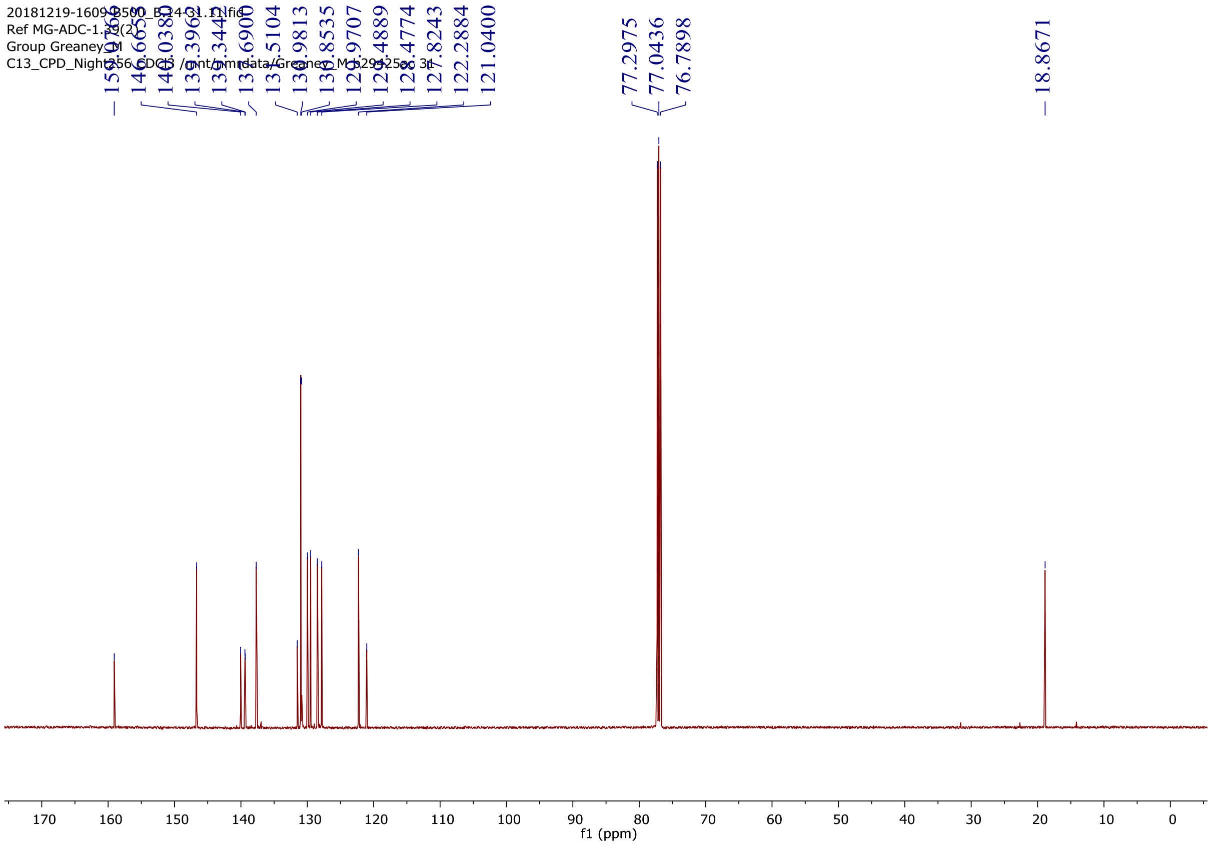

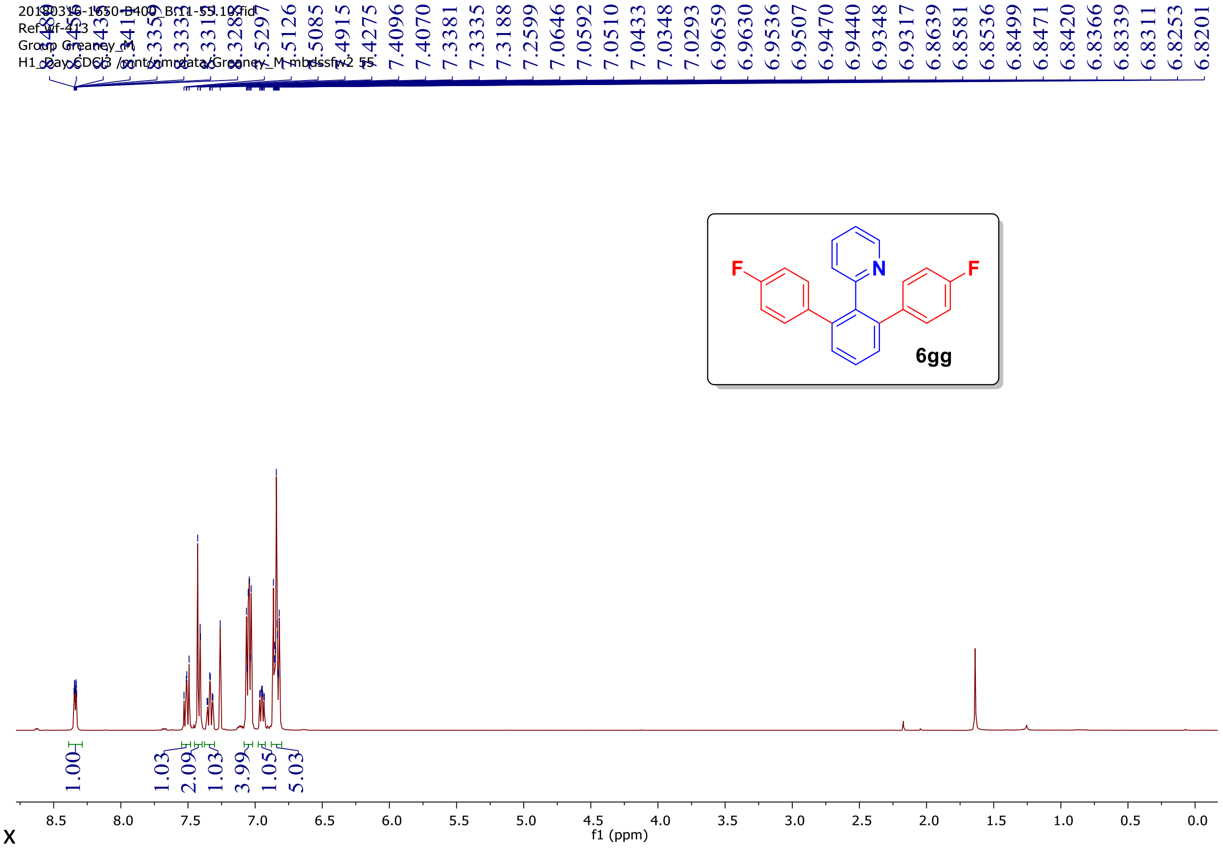
20180316-1650-B400_B11-55.10.fid
Ref wf-413
Group Greaney_M
H1_Day CDCl3 /mnt/nmrdata/Greaney_M mbdssfw2 55
7.5297
7.5126
7.5085
7.4915
7.4275
7.4096
7.4070
7.3381
7.3335
7.3188
7.2599
7.0646
7.0592
7.0510
7.0433
7.0348
7.0293
6.9659
6.9630
6.9536
6.9507
6.9470
6.9440
6.9348
6.9317
6.8639
6.8581
6.8536
6.8499
6.8471
6.8420
6.8366
6.8339
6.8311
6.8253
6.8201
F
N
F
6gg
1.00
1.03
2.09
1.03
3.99
1.05
5.03
f1 (ppm)

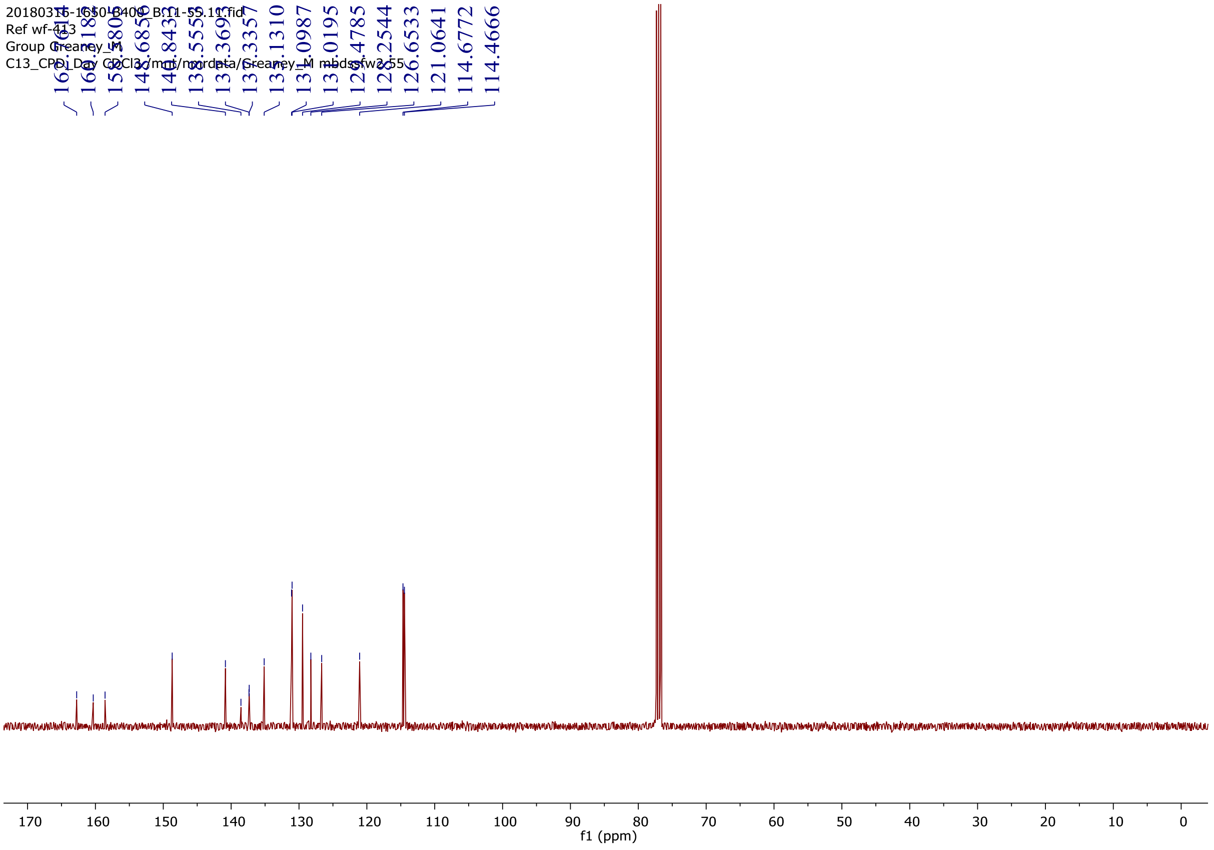
20180316-1650-B400_B11-55.11.fid
Ref wf-413
Group Greaney_M
C13_CPD_Day CDCl3 /mnt/nmrdata/Greaney_M mbdssfw2 55
131.1310
131.0987
131.0195
126.6533
121.0641
114.6772
114.4666
f1 (ppm)

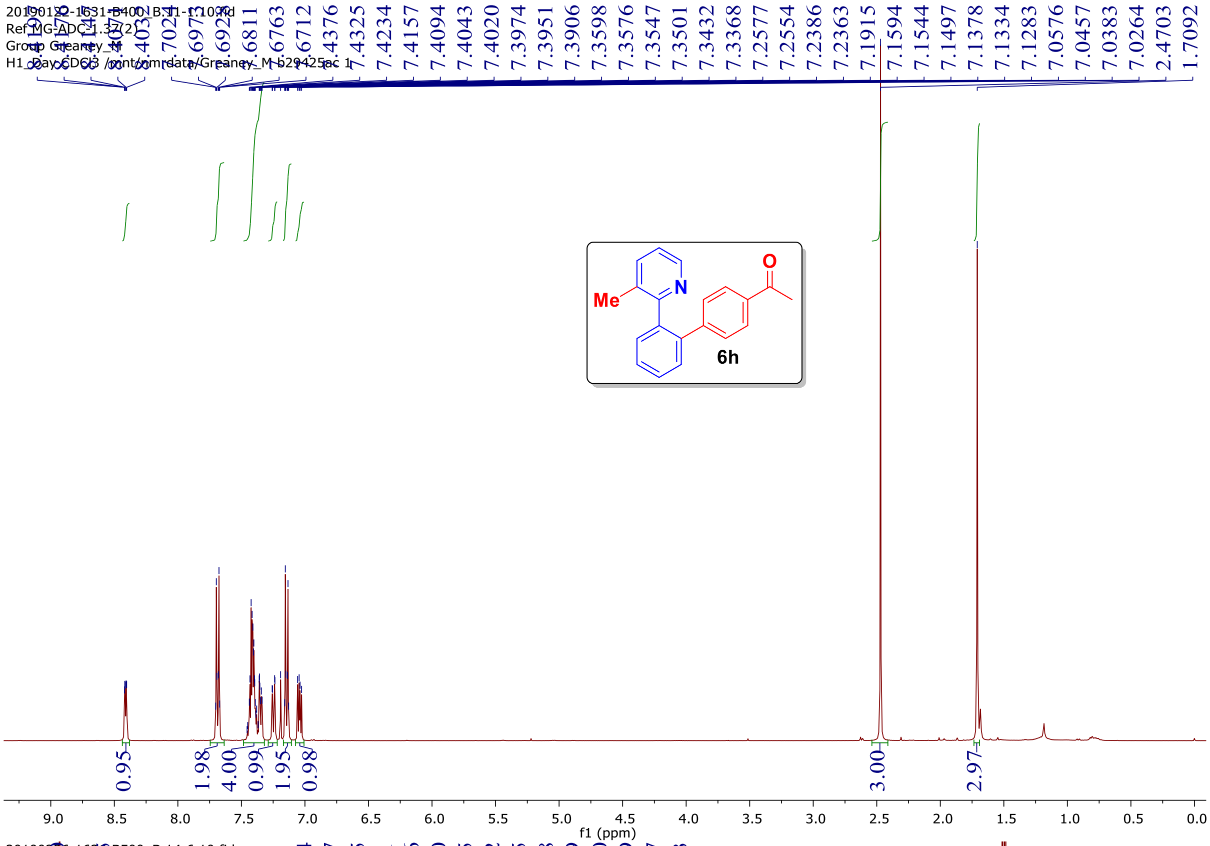

Ref MG-ADC-1.37(2)
Group Greaney_M
7.6811
7.6763
7.6712
7.4376
7.4325
7.4234
7.4157
7.4094
7.4043
7.4020
7.3974
7.3951
7.3906
7.3598
7.3576
7.3547
7.3501
7.3432
7.3368
7.2577
7.2554
7.2386
7.2363
7.1915
7.1594
7.1544
7.1497
7.1378
7.1334
7.1283
7.0576
7.0457
7.0383
7.0264
2.4703
1.7092
Me
N
O
6h
0.95
1.98
4.00
0.99
1.95
0.98
3.00
2.97
9.0 8.5 8.0 7.5 7.0 6.5 6.0 5.5 5.0 4.5 4.0 3.5 3.0 2.5 2.0 1.5 1.0 0.5 0.0
f1 (ppm)

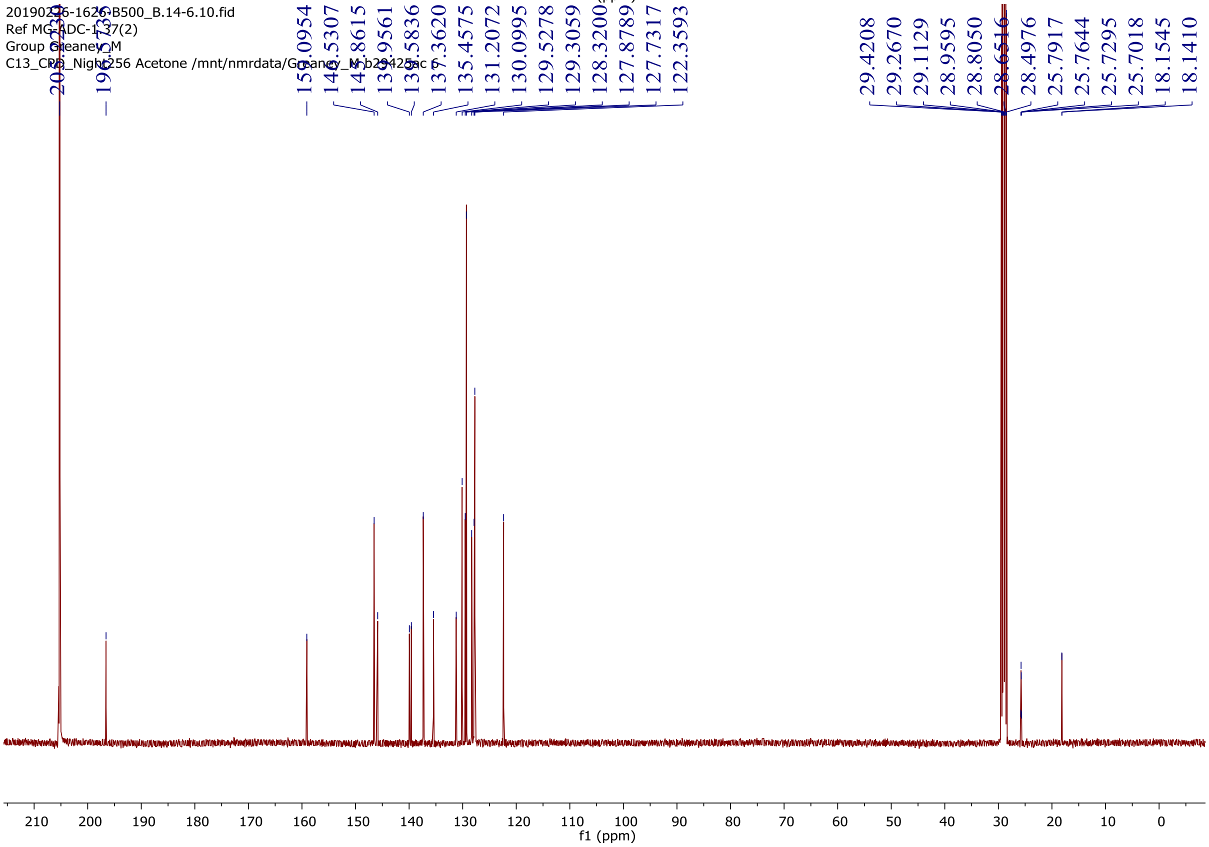

Group Greaney_M
135.4575
131.2072
130.0995
129.5278
129.3059
128.3200
127.8789
127.7317
122.3593
29.4208
29.2670
29.1129
28.9595
28.8050
28.4976
25.7917
25.7644
25.7295
25.7018
18.1545
18.1410
210 200 190 180 170 160 150 140 130 120 110 100 90 80 70 60 50 40 30 20 10 0
f1 (ppm)

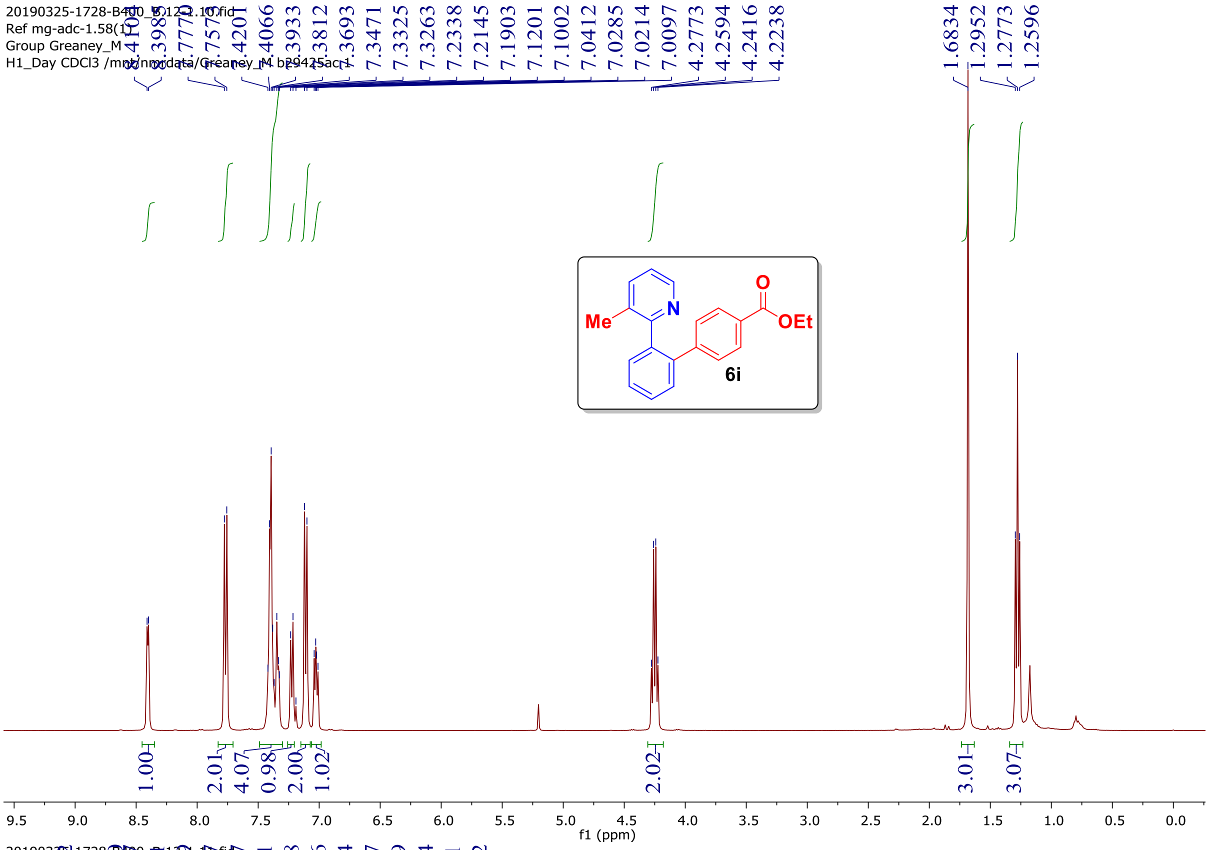
20190325-1728-B400_B:12-1.10.fid
Ref mg-adc-1.58(1)
Group Greaney_M
H1_Day CDCl3 /mnt/nmrdata/Greaney_M b29425ac 1
7.4066
7.3933
7.3812
7.3693
7.3471
7.3325
7.3263
7.2338
7.2145
7.1903
7.1201
7.1002
7.0412
7.0285
7.0214
7.0097
4.2773
4.2594
4.2416
4.2238
1.6834
1.2952
1.2773
1.2596
Me
N
O
OEt
6i
1.00
2.01
4.07
0.98
2.00
1.02
2.02
3.01
3.07
f1 (ppm)

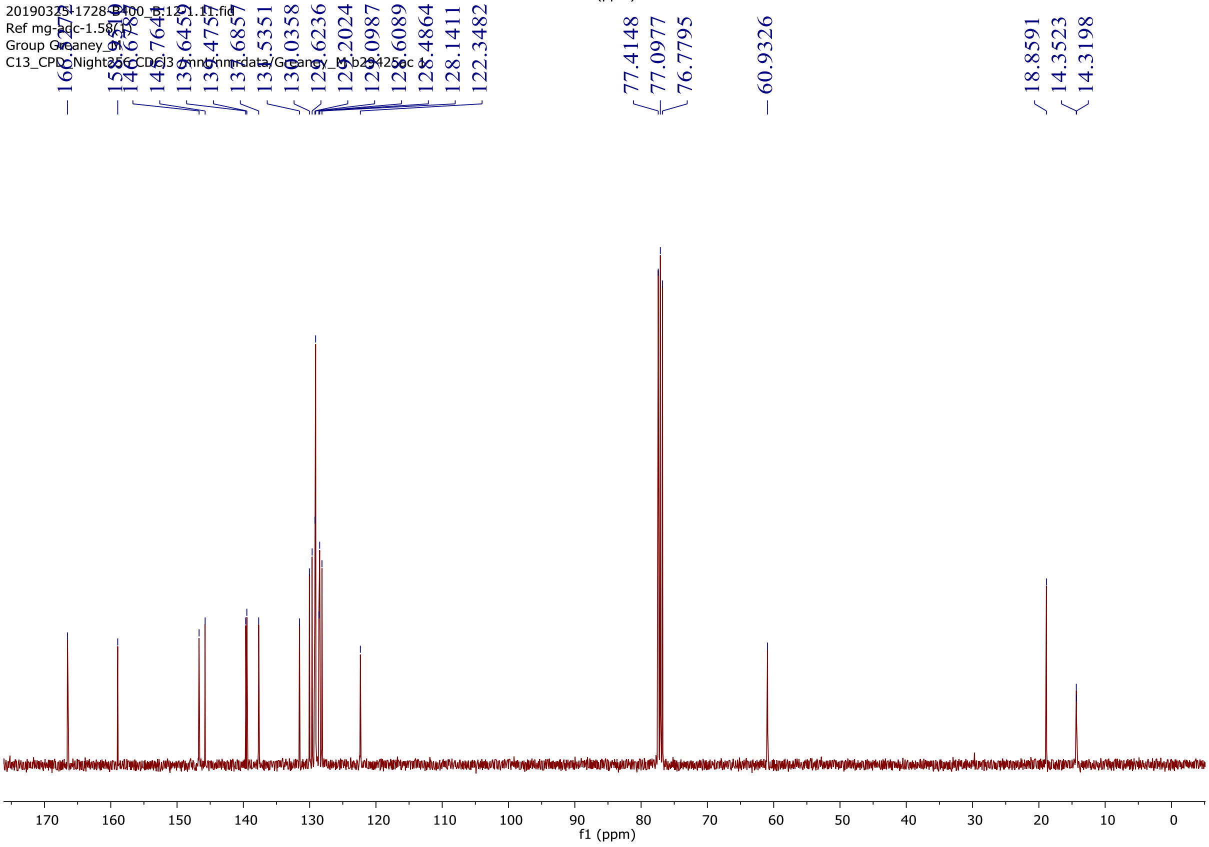
20190325-1728-B400_B:12-1.11.fid
Ref mg-adc-1.58(1)
Group Greaney_M
C13_CPD_Night256 CDCl3 /mnt/nmrdata/Greaney_M b29425ac 1
133.5351
133.0358
128.1411
122.3482
77.4148
77.0977
76.7795
60.9326
18.8591
14.3523
14.3198
f1 (ppm)

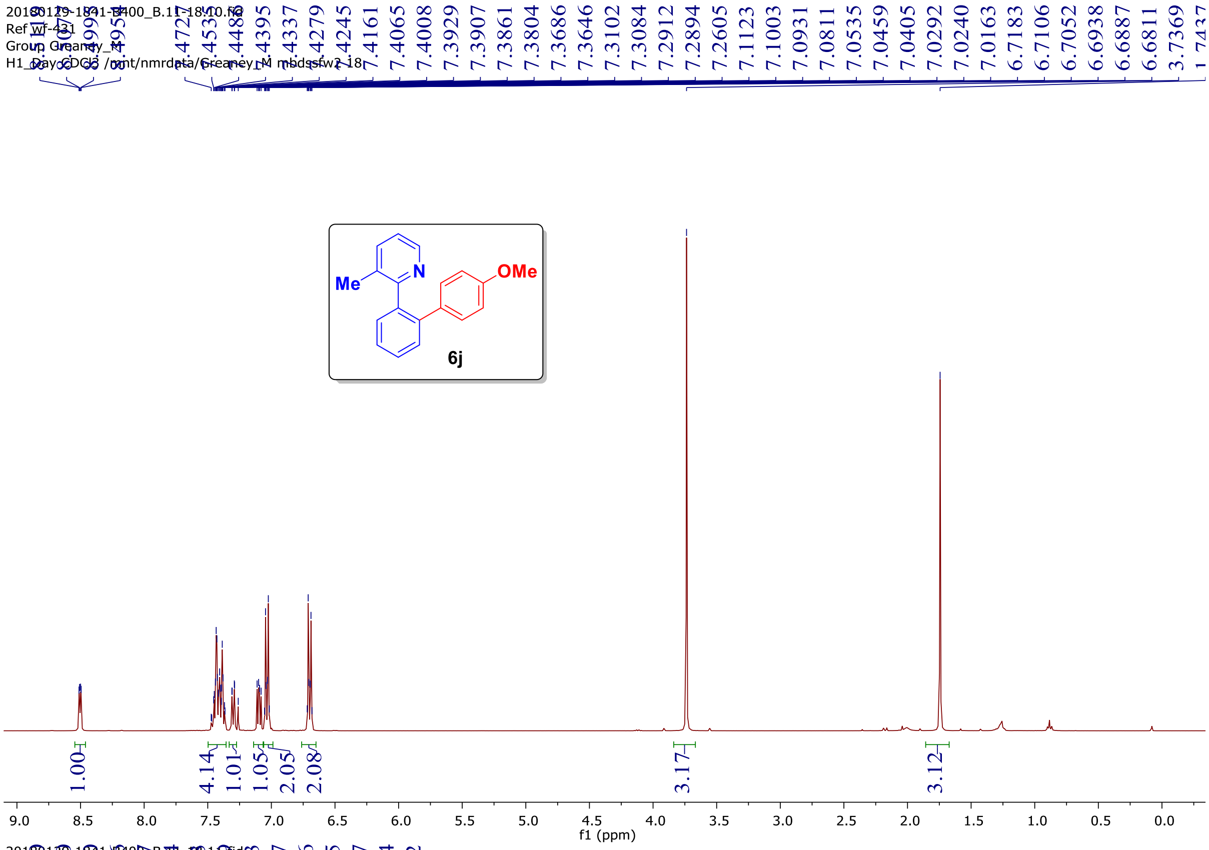

Ref wf-431
Group Greaney_M
7.4395
7.4337
7.4279
7.4245
7.4161
7.4065
7.4008
7.3929
7.3907
7.3861
7.3804
7.3686
7.3646
7.3102
7.3084
7.2912
7.2894
7.2605
7.1123
7.1003
7.0931
7.0811
7.0535
7.0459
7.0405
7.0292
7.0240
7.0163
6.7183
6.7106
6.7052
6.6938
6.6887
6.6811
3.7369
Me
N
OMe
6j
1.00
4.14
1.01
1.05
2.05
2.08
3.17
3.12
f1 (ppm)

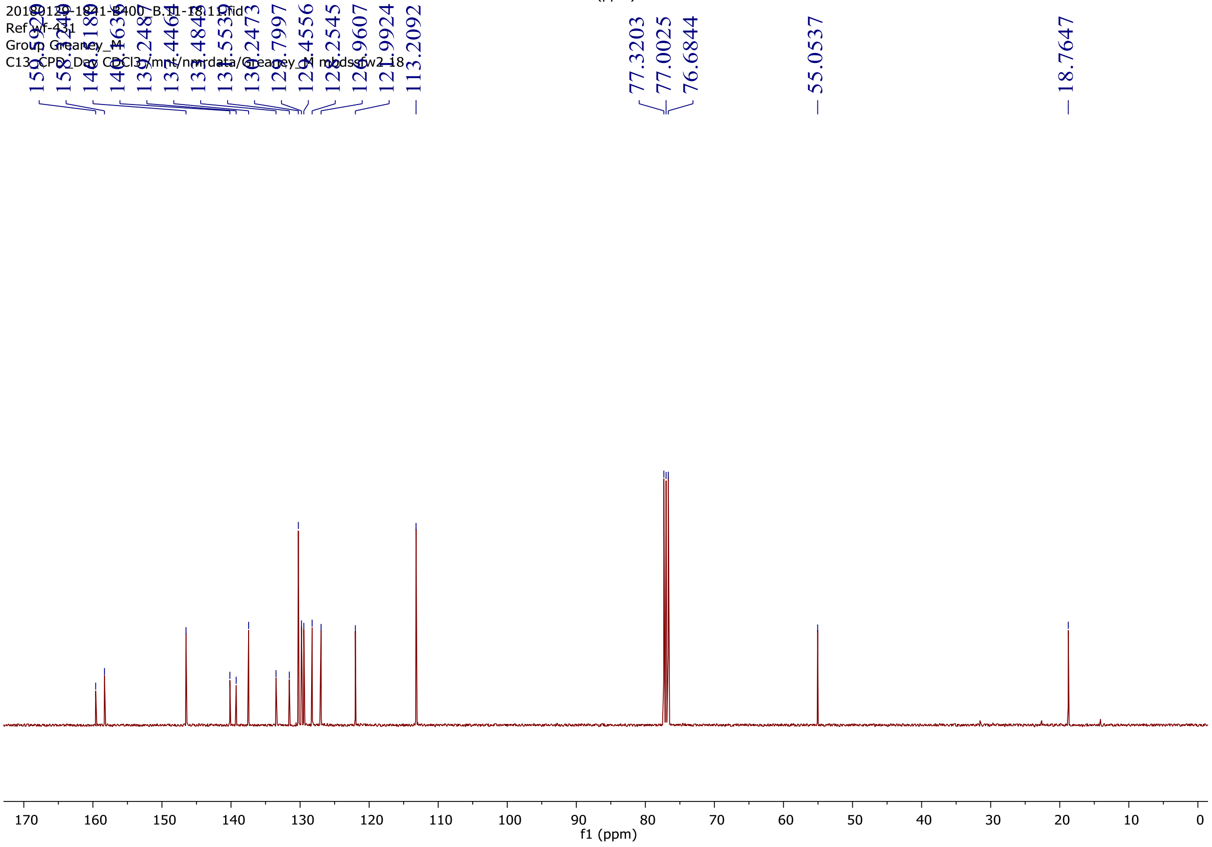

Ref wf-431
Group Greaney_M
113.2092
77.3203
77.0025
76.6844
55.0537
18.7647
f1 (ppm)

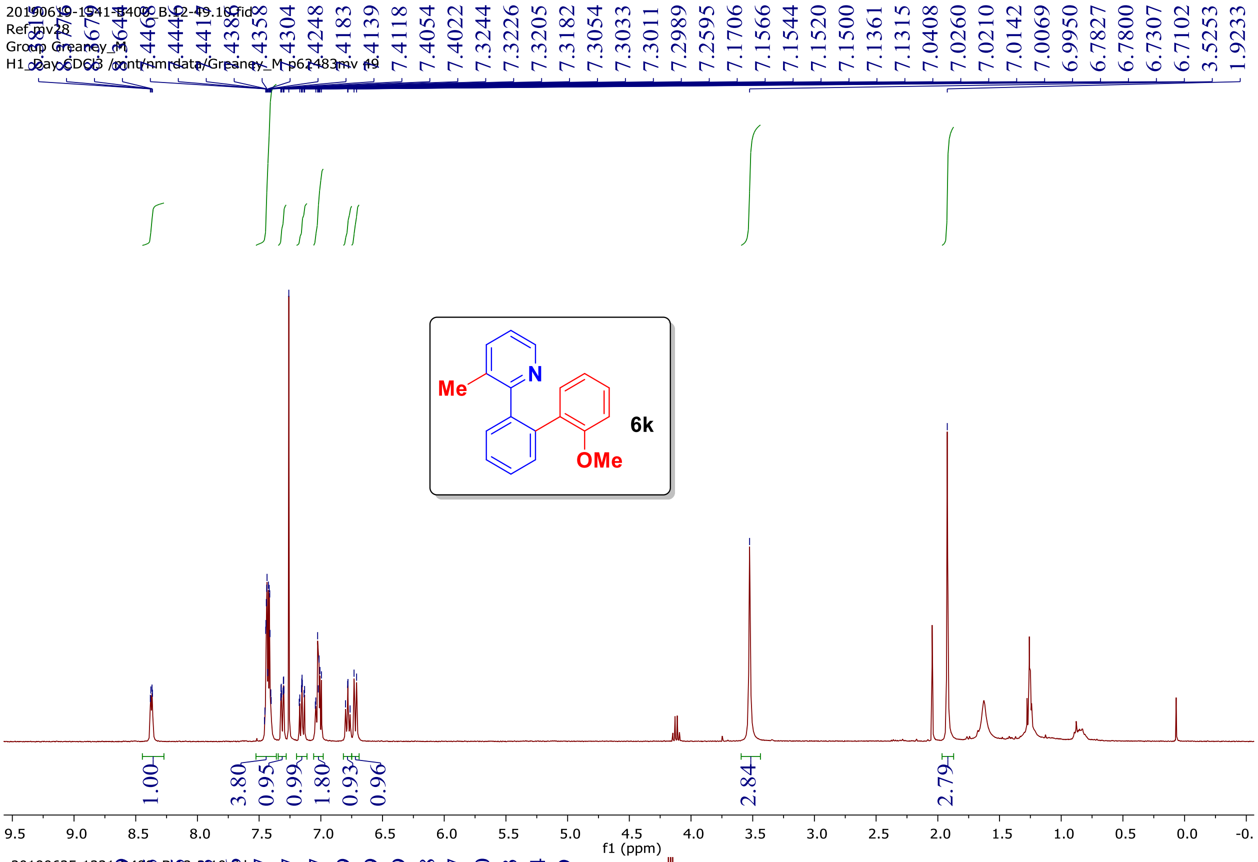
20190619-1541-B400_B-12-49.10.fid
Ref mv28
Group Greaney_M
H1_Day CDCl3 /mnt/nmrdata/Greaney_M p62483mv 49
7.4358
7.4304
7.4248
7.4183
7.4139
7.4118
7.4054
7.4022
7.3244
7.3226
7.3205
7.3182
7.3054
7.3033
7.3011
7.2989
7.2595
7.1706
7.1566
7.1544
7.1520
7.1500
7.1361
7.1315
7.0408
7.0260
7.0210
7.0142
7.0069
6.9950
6.7827
6.7800
6.7307
6.7102
3.5253
1.9233
Me
N
OMe
6k
1.00
3.80
0.95
0.99
1.80
0.93
0.96
2.84
2.79
f1 (ppm)

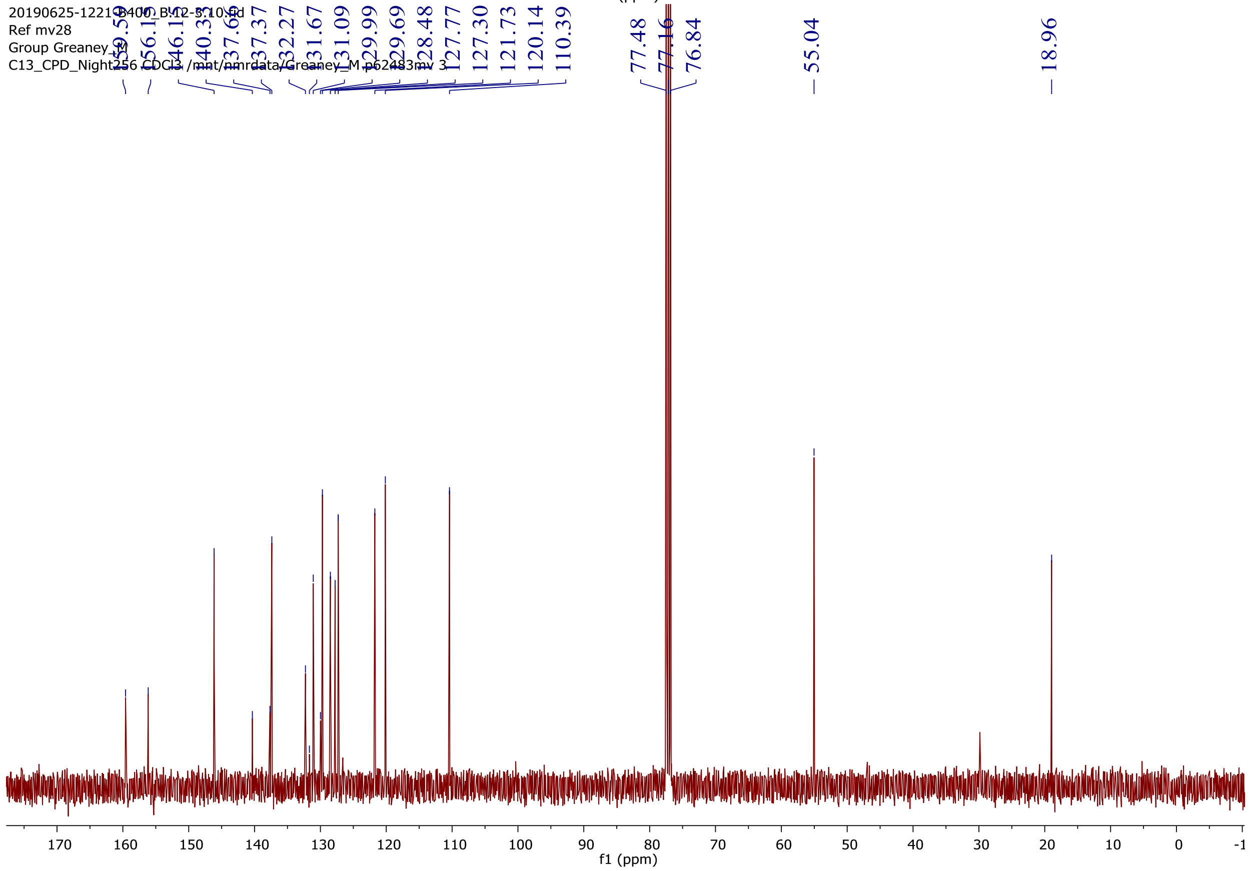
20190625-1221-B400_B-12-3.10.fid
Ref mv28
Group Greaney_M
C13_CPD_Night256 CDCl3 /mnt/nmrdata/Greaney_M p62483mv 3
137.37
132.27
131.67
131.09
129.99
129.69
128.48
127.77
127.30
121.73
120.14
110.39
77.48
77.16
76.84
55.04
18.96
f1 (ppm)
